# Facial mask acute effects on affective/psychological and exercise performance responses during exercise: A meta-analytical review

**DOI:** 10.3389/fphys.2022.994454

**Published:** 2022-11-02

**Authors:** Marcelo Henrique Glänzel, Igor Martins Barbosa, Esthevan Machado, Samuel Klippel Prusch, Ariadine Rodrigues Barbosa, Luiz Fernando Cuozzo Lemos, Felipe Barreto Schuch, Fábio Juner Lanferdini

**Affiliations:** ^1^ Biomechanics Laboratory, Federal University of Santa Maria, Santa Maria, RS, Brazil; ^2^ Biomechanics and Kinesiology Research Group, Federal University of Rio Grande do Sul, Porto Alegre, RS, Brazil; ^3^ Graduate Program in Gerontology, Federal University of Santa Maria, Santa Maria, RS, Brazil; ^4^ Physical Activity and Mental Health Research Laboratory, Santa Maria, RS, Brazil

**Keywords:** mask-wearing, physical exercise (EX), pandemic (COVID-19), respirator, face mask

## Abstract

**Background:** Face masks are widely used during the COVID-19 pandemic as one of the protective measures against the viral infection risk. Some evidence suggests that face mask prolonged use can be uncomfortable, and discomfort can be exacerbated during exercise. However, the acute responses of mask-wearing during exercise on affective/psychological and exercise performance responses is still a topic of debate.

**Purpose:** To perform a systematic review with meta-analysis of the acute effects of mask-wearing during exercise on affective/psychological and exercise performance responses in healthy adults of different/diverse training status.

**Methods:** This review (CRD42021249569) was performed according to Cochrane’s recommendations, with searches performed in electronic (PubMed, Web of Science, Embase, SportDiscus, and PsychInfo) and pre-print databases (MedRxiv, SportRxiv, PsyArXiv, and Preprint.Org). Syntheses of included studies’ data were performed, and the RoB-2 tool was used to assess the studies’ methodological quality. Assessed outcomes were affective/psychological (discomfort, stress and affective responses, fatigue, anxiety, dyspnea, and perceived exertion) and exercise performance time-to-exhaustion (TTE), maximal power output (PO_MAX_), and muscle force production] parameters. Available data were pooled through meta-analyses.

**Results:** Initially 4,587 studies were identified, 36 clinical trials (all crossover designs) were included. A total of 749 (39% women) healthy adults were evaluated across all studies. The face mask types found were clothing (CM), surgical (SM), FFP2/N95, and exhalation valved FFP2/N95, while the most common exercises were treadmill and cycle ergometer incremental tests, beyond outdoor running, resistance exercises and functional tests. Mask-wearing during exercise lead to increased overall discomfort (SMD: 0.87; 95% CI 0.25–1.5; *p* = 0.01; I^2^ = 0%), dyspnea (SMD: 0.40; 95% CI 0.09–0.71; *p* = 0.01; I^2^ = 68%), and perceived exertion (SMD: 0.38; 95% CI 0.18–0.58; *p* < 0.001; I^2^ = 46%); decreases on the TTE (SMD: −0.29; 95% CI −0.10 to −0.48; *p* < 0.001; I^2^ = 0%); without effects on PO_MAX_ and walking/running distance traveled (*p* > 0.05).

**Conclusion:** Face mask wearing during exercise increases discomfort (large effect), dyspnea (moderate effect), and perceived exertion (small effect), and reduces the TTE (small effect), without effects on cycle ergometer PO_MAX_ and distance traveled in walking and running functional tests. However, some aspects may be dependent on the face mask type, such as dyspnea and perceived exertion.

**Systematic Review Registration**: [https://www.crd.york.ac.uk/prospero/display_record.php?ID=CRD42021249569], identifier [CRD42021249569].

## 1 Introduction

The world witnessed the emergence of a new virus in China. The virus was later termed severe acute respiratory syndrome coronavirus 2 (SARS-CoV-2) and defined as the causal agent of the disease known as coronavirus disease 2019—COVID-19 (Ludwig and Zarbock, 2020). From this, health control agencies describe that social distancing is one of the main measures to prevent the spread of the disease, and personal and environmental protection measures and surface hygiene would be essential to avoid contagion (Aquino et al., 2020). Among the protective means, face mask wearing was considered one of the main resources capable of slowing down the advance of the pandemic (Asadi-Pooya and Cross, 2020; Chu et al., 2020). Face mask use is widely discussed during the COVID-19 pandemic and its used is defended by experts as it is a simple, inexpensive, and potentially effective measure to reduce the transmission of respiratory diseases (Asadi et al., 2020; Asadi-Pooya and Cross, 2020). However, the protection offered by the face mask wearing seems to depend on the type of mask, with models that are more or less effective in containing the emission of aerosol particles (Asadi et al., 2020; Fischer et al., 2020; Ramirez-Moreno et al., 2020), such as FFP2/N95 respirators, that provide greater protection but also have greater resistance than surgical masks (SM) (Hopkins et al., 2021).

Beyond the discussions about its effectiveness against coronavirus infection (Asadi et al., 2020; Liang et al., 2020), there are reports of prolonged use of the face mask causing skin lesions, headaches, discomfort and malaise, signs of stress, anxiety, and claustrophobia (Ramirez-Moreno et al., 2020). It may occur because the face mask creates a closed-circuit environment of inspired and expired air (Tornero-Aguilera et al., 2021), increasing ventilation due to carbon dioxide (CO_2_) re-inhalation (Hopkins et al., 2021). Inspiration of CO_2_ appears to be the driving force behind the increased ventilation when breathing through a face mask, since a 1-mm Hg increase in alveolar CO_2_ partial pressure appears to be sufficient to increase ventilation (Hopkins et al., 2021). Face mask use lead to increased subjective stress responses and discomfort levels (Andre et al., 2018; Morris et al., 2020; Tornero-Aguilera et al., 2021), and the adverse effects caused by wearing a face mask seem to be potentiated during exercise due to a reduction in the ability to breathe comfortably (Hopkins et al., 2021; Reychler et al., 2021), for these reasons, its use in closed environments (e.g., gyms and training centers) is still much discussed (Hopkins et al., 2021).

Recent systematic reviews (Engeroff et al., 2021; Shaw et al., 2021) investigated the face mask using during exercise on some psychophysiological responses. Face mask wearing during exercise seems to increase the perceived exertion and dyspnea (Shaw et al., 2021), impair cardiorespiratory parameters (e.g., oxygen uptake and ventilation) (Engeroff et al., 2021), without affecting oxygen saturation, and heart rate (Engeroff et al., 2021; Shaw et al., 2021). Although there is already evidence of the face mask using effects on physiological outcomes, no systematic review or meta-analysis focused on quantifying the magnitude of the effects of face mask wearing during exercise exclusively on affective/psychological outcomes. Furthermore, the effects stratified by type of face mask need to be further explored.

Considering that mask wearing may cause discomfort and this could compromise the exercise performance parameters (Motoyama et al., 2016; Andre et al., 2018; Fikenzer et al., 2020; López-Pérez et al., 2020; Tornero-Aguilera et al., 2021; Boyle et al., 2022), the acute effects of face mask use were investigated through different exercise performance aspects. Previous systematic reviews (Engeroff et al., 2021; Shaw et al., 2021) showed no effects of face mask wearing on exercise performance. However, exercise performance was pooled through different outcomes. The literature shows different physical performance protocols tested comparing with and without face mask use. Decreases in the time-to-exhaustion (TTE) (Öncen and Pinar, 2018; Driver et al., 2021; Boyle et al., 2022), maximal power output (PO_MAX_) in cycle ergometer (Fikenzer et al., 2020; Egger et al., 2021; Zhang et al., 2021), total volume and the maximum number of repetitions during resistance exercises (Rosa et al., 2022), and performance in sprint tests (Dantas et al., 2021; Modena et al., 2021; Tornero-Aguilera et al., 2021) were reported. However, there seems to be no consensus in the literature on the occurrence of these effects. In addition, we have not found systematic reviews so far that have investigated the effects of face mask use exclusively on exercise performance parameters.

Knowledge about the acute effects of face mask wearing during exercise on affective/psychological aspects and their potential adverse effects on exercise performance outcomes is still unclear, especially when considering the different types of face mask. This gap in the literature shows that this systematic review is relevant and can be useful to help physical therapists and trainers understand and make decisions regarding the acute effects of the face mask wearing during exercise. Therefore, we aimed to perform a systematic review with meta-analysis of trials that tested the face mask acute effects on affective/psychological and exercise performance responses during exercise in healthy adults of different/diverse training status.

## 2 Methods

### 2.1 Study reporting and protocol registration

A systematic review with meta-analysis was performed following the recommendations from the Cochrane Collaboration (Higgins et al., 2019) and the PRISMA (Preferred Reporting Items for Systematic Reviews and Meta-Analyzes) reporting guidelines (Page et al., 2021). The review protocol was registered on PROSPERO under the number CRD42021249569. The study selection, data extraction, and the methodological quality assessment of the included studies were conducted by two independent investigators (M.H.G. and E.M.) and when there was some disagreement between the results of the two reviewers, a third reviewer (S.K.P.) was consulted to reach a consensus.

### 2.2 Data sources and searches

In March 2022, searches were performed in five electronic databases (ISI Web Knowledge, MEDLINE/Pubmed, Embase, SportDiscus, and PsychInfo) and preprint databases (MedRxiv, SportRxiv, PsyArXiv, Preprint.Org) without time and language restrictions. Furthermore, reference lists of relevant reviews (Feye and Magallanes, 2019; Andreu, 2021; Haraf et al., 2021; Shaw et al., 2021) and included trials were manually searched. Combinations were used with the descriptors and keywords adapted for each database (adult OR athletes) AND (mask OR “respiratory protection device”) AND (exercise OR sport OR “physical activity” OR athletic performance” OR “aerobic exercise” OR “resistance training”). The searches were combined using the terms MeSH or EMTREE, and using Boolean operators “AND” and “OR.” Full details of the search strategy are presented in the Supplementary Table S2.

### 2.3 Study selection and screening criteria

The present review was composed of a three-stage screening process, which was conducted by two independent reviewers (M.H.G. and I.M.B.). When there was between-reviewers disagreement, a third reviewer (S.K.P.) was consulted to reach a consensus. Initially, were screened titles and excluded irrelevant papers (e.g., *in vitro* studies). In the second stage, the reviewers screened studies by abstracts. In the last stage, the full text of the studies was assessed according to the eligibility criteria.

The inclusion criteria adopted were: 1) randomized and non-randomized controlled trial; 2) healthy adults (described as ages between 19 and 44 years by Medical Subject Headings); 3) face mask wearing during sports practices, aerobic and/or resistance exercise intervention; 4) control condition with a no-wearing face mask during exercise; and 5) assessment of affective/psychological and/or exercise performance parameters. Studies that did not meet all the inclusion criteria or that presented the following exclusion criteria were excluded: 1) measurements of chronic effects; 2) other facial mask types (e.g., elevation training mask; air-laine breathing apparatus; self-contained breathing apparatus, sport protective helmets); and 3) no accessible full-text.

Randomized and non-randomized controlled trials (crossovers or parallel-group studies) with the full and accessible text were included in this review. Primary outcomes considered were as acute effects of facial mask in: 1) affective/psychological parameters (discomfort, stress and affective responses, fatigue, anxiety, dyspnea, and perceived exertion); 2) exercise performance parameters [TTE, PO, bar propulsive velocity (BPV), number of repetitions, muscle force production, and walk/running acceleration, speed, and time]. The secondary outcomes were: 1) face mask type [i.e., clothing (CM), SM, FFP2/N95, and FFP2/N95 with exhalation valve (FFP2/N95 + EV)]; 2) exercise type, volume, and intensity.

### 2.4 Data extraction

Searches on databases were completed by 7 March 2022. The data from each study were extracted individually and exported to a spreadsheet. Study design, sample size, participant characteristics, face mask type, exercise (type, volume, and intensity), measured outcomes (main outcomes and assessments), and results [intervention (face mask wearing) and control (no-face mask wearing) pre-post changes by mean and standard deviation values] were extracted.

Data from both crossover and parallel-group studies were included. When studies did not provide enough data (i.e., incomplete reporting), the corresponding author of the study was contacted by email and asked to provide additional information. Case the database was provided by the authors, the mean and standard deviation values were calculated for quantitative analysis (Epstein et al., 2021; Fukushi et al., 2021; Modena et al., 2021). When authors did not respond or could not provide the required data, the mean and standard deviation values were obtained manually from the plots using the ImageJ tool (version 1.48v, National Institutes of Health, Bethesda, MA, United States), whenever possible. When access to the data was not possible, or in case of incompatible data (e.g., non-groupable exercise), the study was not included in the quantitative analysis.

### 2.5 Data synthesis and statistics

Common outcomes among three or more studies were considered for the meta-analyses using the standardized mean difference (SMD), standard error (SE), and 95% confidence interval (95% CI) as measures of effect, dispersion, and range, respectively. The effect size was classified according to Cohen’s d-values (Higgins et al., 2019), where SMD: <0.40 = small effect; 0.40–0.70 = moderate effect; >0.70 = large effect (Cohen, 1988). Therefore, seven meta-analyses (discomfort, dyspnea, perceived exertion, TTE, absolute and relative PO_MAX_, and distance traveled in walking and running functional tests) were performed based on the face mask wearing acute effects during exercise. We have included over 10 studies for dyspnea and perceived exertion, allowing us to perform subgroup analysis on the different face mask types (i.e., CM, SM, FFP2/N95 + EV, and FFP2/N95). When the study presents more than one mask type, for the meta-analysis, the chosen mask was determined from the criterion of the least restrictive face mask to the most restrictive (ascending order: clothing, SM, FFP2/N95 + EV, and FFP2/N95), considering the least restrictive face masks (i.e., those made from cloth) as it is the most used by the general population. When the study performed tests at different intensities, the intensity that generated the greatest metabolic load was adopted for analysis. In studies that used both face masks with and without an exhalation valve, the face mask without a valve was considered for analysis, since it is more common in the general population and during the COVID-19 pandemic.

In the crossover trials, when a study did not present the correlation coefficient (r) values between the pre-post changes, a sensitivity analysis using different estimate values (*r* = 0.5; *r* = 0.7; and *r* = 0.8) was performed. As none of the values directly affected the results of the meta-analyses, we adopted a conservative estimate of “*r* = 0.7.” For all the meta-analyses, we used the random-effects model. This model was adopted *a piori* due to the expected heterogeneity in the studies’ intervention types, and the evaluation of the common measures, and confirmed by the I^2^ test, which was interpreted according to Higgins et al. (2003) considering that the values above 25% and 50% were classified as moderate and high heterogeneity, respectively. When moderate or high heterogeneity was found (values > 25%), sensitivity analysis was performed and the heterogeneity was explored. All statistical analyses were performed using the Comprehensive Meta-Analysis (version 3.0; Biostat, Englewood, NJ, United States). The level of statistical significance was determined as α ≤ 0.05.

### 2.6 Studies’ quality appraisal

The methodological quality of the included studies was examined by two independent reviewers (E.M. and A.R.B.), with a third reviewer consulted in cases of disagreement (M.H.G.). The assessment tool was the Revised Cochrane Risk-of-Bias Tool for Randomized Trials (ROB 2) for parallel-group and crossover trials (Sterne et al., 2019). This tool was used assignment to intervention (the “intention-to-treat” effect) and has five domains for judgment of risk of bias: randomization process; deviations from intended intervention, missing outcome data; result measurement; selection of the reported result. Each domain was rated as “low risk of bias,” “high risk of bias,” and “some concerns” for the reported outcomes, and the overall risk of bias judgment: low risk of bias—all domains showed a low risk of prejudice; some concerns: in at least one domain for this outcome but not being at high risk of bias for any domain, and high risk of bias—when in at least one domain for this outcome or have some concerns for multiple domains in some way that substantially reduces confidence in the result.

## 3 Results

### 3.1 Search results

**FIGURE 1:**
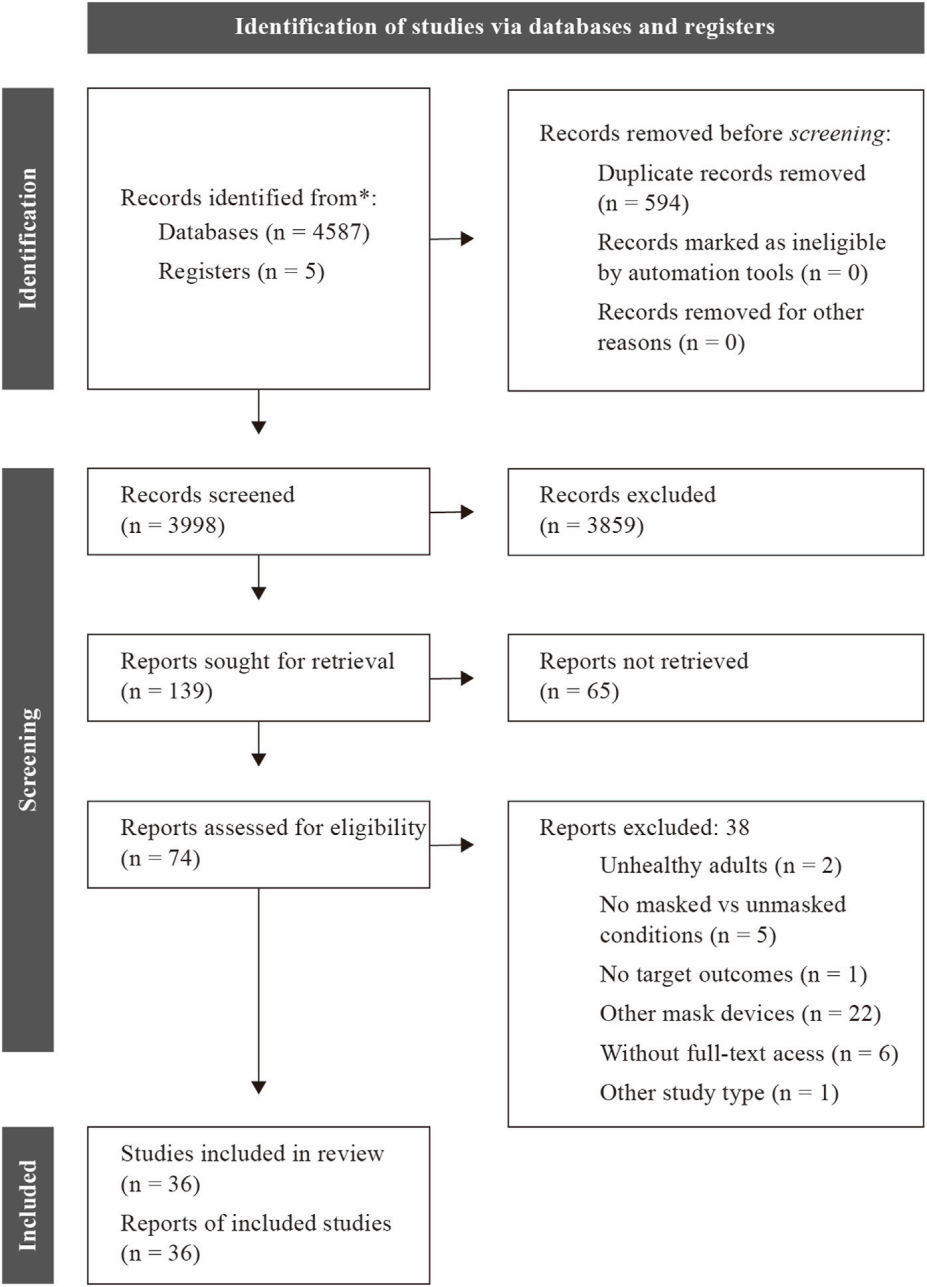
PRISMA chart of the study flow.

A flow diagram of the literature search and screening is displayed below (Figure 1). The initial search identified 4,587 studies in all combined databases. In the full-text stage, 69 studies were eligible, and in the end, 34 studies (Zimmerman et al., 1991; Roberge et al., 2010; Roberge et al., 2012a; Roberge et al., 2012b; Chen et al., 2016; Kim et al., 2016; Person et al., 2018; Fikenzer et al., 2020; Lässing et al., 2020; Morris et al., 2020; Wong et al., 2020; Ade et al., 2021; Bar-On et al., 2021; Dantas et al., 2021; Doherty et al., 2021; Driver et al., 2021; Egger et al., 2021; Epstein et al., 2021; Fukushi et al., 2021; Kampert et al., 2021; Mapelli et al., 2021; Modena et al., 2021; Reychler et al., 2021; Rojo-Tirado et al., 2021; Ryu and Jong-Geun, 2021; Slimani et al., 2021; Tornero-Aguilera et al., 2021; Yoshihara et al., 2021; Zhang et al., 2021; Cabanillas-Barea et al., 2022; Jesus et al., 2022; Ng et al., 2022; Rosa et al., 2022; Steinhilber et al., 2022) met all the eligibility criteria and were included in the review. Two additional studies (Shaw et al., 2020; Otsuka et al., 2022) were found from other sources (e.g., references from included studies, and publications founded in other reviews). Therefore, 36 eligible studies were included in the review.

### 3.2 Characteristics of the included studies

Table 1 summarizes the main information of the included studies. Most studies were randomized controlled trials (*n* = 34), except for two studies (Roberge et al., 2012a; Roberge et al., 2012b) that did not perform randomization. The crossover design was adopted by all the studies. All studies presented a control condition (i.e., no face mask wearing). The samples were composed mostly by healthy (Zimmerman et al., 1991; Roberge et al., 2010; Roberge et al., 2012a; Roberge et al., 2012b; Chen et al., 2016; Kim et al., 2016; Person et al., 2018; Lässing et al., 2020; Morris et al., 2020; Bar-On et al., 2021; Doherty et al., 2021; Driver et al., 2021; Fukushi et al., 2021; Mapelli et al., 2021; Reychler et al., 2021; Ryu and Jong-Geun, 2021; Slimani et al., 2021; Zhang et al., 2021; Cabanillas-Barea et al., 2022; Jesus et al., 2022; Steinhilber et al., 2022), healthy physically active (Fikenzer et al., 2020; Shaw et al., 2020; Wong et al., 2020; Ade et al., 2021; Epstein et al., 2021; Kampert et al., 2021; Yoshihara et al., 2021), healthy trained (Rojo-Tirado et al., 2021; Ng et al., 2022) resistance-trained (Rosa et al., 2022), and healthy sedentary (Otsuka et al., 2022) adults; athletes (Dantas et al., 2021; Egger et al., 2021; Tornero-Aguilera et al., 2021) and amateur soccer players (Modena et al., 2021). The sample size varied from six (Otsuka et al., 2022) to 72 (Tornero-Aguilera et al., 2021) participants per condition. Some studies (*n* = 10) (Person et al., 2018; Shaw et al., 2020; Ade et al., 2021; Bar-On et al., 2021; Driver et al., 2021; Reychler et al., 2021; Rojo-Tirado et al., 2021; Zhang et al., 2021; Ng et al., 2022; Steinhilber et al., 2022) determined the sample size based on sample calculation. A total of 749 participants: 460 men and 289 women; with a mean age of 27 years old, were evaluated across all studies.

**TABLE 1:** Summary of studies on the acute effects of comparison between use of mask versus no-mask wearing during exercise.

Study	Design/Mask type	Participants	Exercise protocol	Outcomes
Ade et al. (2021)	RCT	Healthy and physically active adults.	Incremental cycle ergometer exercise to exhaustion (increases of 20 W/min; until the participant could not maintain the pedal cadence of 60 rpm for 5 consecutive revolutions).	-Affective/psychological responses -Dyspnea: ↑9%
	Crossover design	(*n* = 11; M = 5; W = 6)	
	1: CON	30 ± 11 years		RPE: ns
	2: CM			-Exercise performance responses
	3: SM			PO_MAX_: ns
	4: FFP2/N95			
Bar-On et al. (2021)	RCT	Healthy adults.	Constant load treadmill walking for 5 min: a) slow walk (4 km/h at 0° inclination); b) brisk walk (7 km/h at 0° inclination).	-Affective/psychological responses
	Crossover design	(*n* = 10; M = 5; W = 5)		RPE: ^ab^↑39%
	1: CON	28 ± 5 years		
	2: SM			
Cabanillas-Barea et al. (2022)	RCT	Healthy adults., (*n* = 50; M = 26; W = 24), 21 ± 5 years	6 MWT	-Affective/psychological responses, Dyspnea: ↑61%–129%, -Exercise performance responses, Distance traveled: ns
	Crossover design			
	1: CON			
	2: SM			
	3: FFP2/N95			
Chen et al. (2016)	RCT	Healthy adults., (*n* = 15; M = 15; W = 0), 28 ± 2 years	Constant load treadmill walking for 5 min (treadmill speed of 1.6 m/s and 0° inclination).	-Affective/psychological responses, Dyspnea: ↑369%–431%
	Crossover design			
	1: CON			
	2: FFP2/N95			
	3: FFP2/N95 + EV			
Dantas et al. (2021)	RCT	Track and field athletes., (*n* = 10; M = 7; W = 3), 23 ± 4 years	Outdoor track field running test (five maximal 30 m sprints, with 4 min rest between runs); and vertical jump (countermovement jump).	-Affective/psychological responses, Affect: ↓114%–148%, RPE: ↑46%, -Exercise performance responses, Sprint time: ns, Sprint acceleration: ns, Jump height: ns
	Crossover design			
	1: CON			
	2: CM			
Doherty et al. (2021)	RCT	Healthy adults., (*n* = 12; M = 7; W = 5), 26 ± 3 years	Constant load cycle ergometer submaximal exercise for 8 min (submaximal exercise at 70% HR_MAX_).	-Affective/psychological responses, Dyspnea: ↑42%
	Crossover design			
	1: CON (MP)			
	2: CON			
	3: CM			
	4: SM			
Driver et al. (2021)	RCT	Healthy adults., (*n* = 31; M = 17; W = 14), 23 ± 3 years	Incremental treadmill cardiopulmonary exercise test (Bruce’s standard protocol).	-Affective/psychological responses, RPE: ns, Dyspnea: ↑31%, -Exercise performance responses, TTE: ↓14%
	Crossover design			
	1: CON			
	2: CM			
Egger et al. (2021)	RCT	Well-trained healthy athletes (2 road cyclists; 8 mountain bikers, and 6 triathletes), (*n* = 16; M = 16; W = 0), 27 ± 7 years	Incremental cycle ergometer exercise to exhaustion (starting at 100–150 W; increases of 50 W every 3 min until voluntary exhaustion or when subjects were unable to maintain a pedaling cadence of 50 rpm for more than 10 s).	-Affective/psychological responses, RPE: ns, -Exercise performance responses, PO_MAX_: ↓4%–6%
	Crossover design			
	1: CON			
	2: SM			
	3: FFP2/N95			
Epstein et al. (2021)	RCT	Healthy and physically active adults., (*n* = 10; M = 5; W = 5), 28 ± 5 years	Incremental cycle ergometer exercise to exhaustion (starting at 25 W; increases of 25 W every 3 min until voluntary exhaustion; cadence of 55–65 rpm).	-Affective/psychological responses, RPE: ns, -Exercise performance responses, TTE: ns
	Crossover design			
	1: CON			
	2: SM			
	3: FFP2/N95			
Fikenzer et al. (2020)	RCT	Healthy and physically active adults., (*n* = 12; M = 12; W = 0), 38 ± 6 years	Incremental cycle ergometer exercise to exhaustion (starting at 50 W; increases of 50 W every 3 min until voluntary exhaustion; cadence of 60–70 rpm).	-Affective/psychological responses, Overall discomfort: ↑86%–150%, -Exercise performance responses, PO_MAX_: ↓5%
	Crossover design			
	1: CON			
	2: SM			
	3: FFP2/N95			
Fukushi et al. (2021)	RCT	Healthy adults., (*n* = 24; M = 15; W = 9), 21 ± 1 years	Incremental treadmill cardiopulmonary exercise test (Modified Bruce’s standard protocol).	-Affective/psychological responses, RPE: ↑25%–44%
	Crossover design			
	1: CON			
	2: CM			
	3: SM			
Jesus et al. (2022)	RCT	Healthy adults., (*n* = 32; M = 16; W = 16), 24 ± 3 years	Constant load cycle ergometer exercise [two different intensities: a) moderate exercise at 25% below VT; and b) severe exercise at 25% above VT].	-Exercise performance responses, TTE: ^a^ns ^b^↓10%
	Crossover design			
	1: CON			
	2: SM			
Kampert et al. (2021)	RCT	Healthy and physically active adults., (*n* = 20; M = 11; W = 9), M: 39 ± 11 years, W: 35 ± 11 years	Incremental treadmill cardiopulmonary exercise test (constant speed; treadmill grade increased from 0% to 2% at 2 min; and continuous increases by 1% every minute until voluntary exhaustion).	-Affective/psychological responses, RPE: ns, Overall discomfort: ↑60%, -Exercise performance responses, TTE: ns
	Crossover design			
	1: CON			
	2: CM			
	3: FFP2/N95			
Kim et al. (2016)	RCT	Healthy adults., (*n* = 12; M = 12; W = 0), 24 ± 2 years	Constant load treadmill walking for 1 h (low-moderate work rate at 5.6 km/h and 0° inclination).	-Affective/psychological responses, RPE: ns, Dyspnea: ↑23%–41%, Thermal sensation: ns
	Crossover design			
	1: CON			
	2: FFP2/N95			
	3: FFP2/N95 + EV			
Lässing et al. (2020)	RCT	Healthy adults., (*n* = 14; M = 14; W = 0), 26 ± 4 years	Constant load cycle ergometer exercise for 30 min (50% of PO_MAX;_ cadence of 60–70 rpm).	-Affective/psychological responses, RPE: ns
	Crossover design			
	1: CON			
	2: SM			
Mapelli et al. (2021)	RCT	Healthy adults., (*n* = 12; M = 6; W = 6), 41 ± 12 years	Incremental cycle ergometer exercise (aimed to achieving peak exercise in ∼10 min).	-Affective/psychological responses, Dyspnea: ud, -Exercise performance responses, PO_MAX_: ↓4%–5%
	Crossover design			
	1: CON			
	2: SM			
	3: FFP2/N95			
Modena et al. (2021)	RCT	Amateur soccer players., (*n* = 21; M = 21; W = 0), 25 ± 5 years	Running protocol [4 min running at: a) 8 km/h; and b) 10 km/h; 8 bouts of 90 m intermittent running; and YoYo-Intermittent Recovery Test Level-1].	-Affective/psychological responses, RPE: ns, Dyspnea: ns, -Exercise performance responses, Distance traveled: ↓11%–13%
	Crossover design			
	1: CON			
	2: CM			
	3: SM			
Morris et al. (2020)	RCT	Healthy adults., (*n* = 8; M = 8; W = 0), 35 ± 7 years	Light exercise simulating work in healthcare and related settings for 45 min (100 W; equivalent to ∼5 METs).	-Affective/psychological responses, Dyspnea: ↑140%, Facial thermal discomfort: ↑22%
	Crossover design			
	1: CON			
	2: FFP2/N95			
Ng et al. (2022)	RCT	Healthy and trained adults., (*n* = 8; M = 4; W = 4), 25 ± 3 years	Incremental cycle ergometer exercise to exhaustion (starting at 50 W; increases of 25 W every 3 min until voluntary exhaustion).	-Affective/psychological responses, Dyspnea: ns, -Exercise performance responses, TTE: ↓6%, PO_MAX_: ns
	Crossover design			
	1: CON			
	2: SM			
Otsuka et al. (2022)	RCT	Healthy and sedentary adults., (*n* = 6; M = 6; W = 0), 24 ± 2 years	Incremental cycle ergometer exercise to exhaustion (increases of 20 W every minute until voluntary exhaustion).	-Affective/psychological responses, RPE: ns, Dyspnea: ↑30%
	Crossover design			
	1: CON			
	2: SM			
Person et al. (2018)	RCT	Healthy adults., (*n* = 44; M = 18; W = 26), 22 ± 3 years	6 MWT	-Affective/psychological responses, Dyspnea: ↑22%, -Exercise performance responses, Distance traveled: ns
	Crossover design			
	1: CON			
	2: SM			
Reychler et al. (2021)	RCT	Healthy adults., (*n* = 20; M = 11; W = 9), 22 ± 2 years	STS^1min^	-Affective/psychological responses, Dyspnea: ↑100%, Overall discomfort: ↑3%–4%, -Exercise performance responses, T-REPS: ns
	Crossover design			
	1: CON			
	2: CM			
	3: SM			
Roberge et al. (2010)	RCT	Healthy adults., (*n* = 10; M = 3; W = 7), 25 (20–45) years	Constant load treadmill walking for 1 h: a) 1.7 mph; b) 2.5 mph.	-Affective/psychological responses, Overall discomfort: ^ab^ns, RPE: ^ab^ns
	Crossover design			
	1: CON			
	2: FFP2/N95			
	3: FFP2/N95 + EV			
Roberge et al. (2012b)	CT	Healthy adults., (*n* = 20; M = 13; W = 7), 23 ± 3 years	Constant load treadmill walking for 1 h (low-moderate work rate at 5.6 km/h and 0° inclination)	-Affective/psychological responses, RPE: ns
	1: CON			
	2: SM			
Roberge et al. (2012a)	CT	Healthy adults., (*n* = 20; M = 13; W = 7), 23 ± 3 years	Constant load treadmill walking for 1 h (low-moderate work rate at 5.6 km/h and 0° inclination)	-Affective/psychological responses, RPE: ↑8%
	1: CON			
	2: FFP2/N95			
	3: FFP2/N95 + EV			
	4: FFP2/N95			
	5: FFP2/N95 + EV			
Rojo-Tirado et al. (2021)	RCT	Healthy sportswomen., (*n* = 13; M = 0; W = 13), 22 ± 2 years	Incremental treadmill cardiopulmonary exercise test [treadmill grade of 1%; warm-up of 6 km/h for 3 min; starting the incremental phase with 8 km/h of running, with increases of 0.2 km/h every 12 s (1 km/h/min) until it was possible to maintain the treadmill speed].	-Affective/psychological responses, RPE: ns, Dyspnea: ns, -Exercise performance responses, TTE: ns
	Crossover design			
	1: CON			
	2: CM			
	3: FFP2/N95			
Rosa et al. (2022)	RCT	Healthy and resistance-trained adults., (*n* = 17; M = 17; W = 0), 28 ± 4 years	Resistance training with BP exercise [4 sets to failure with resting interval of 2 min between sets, performed at different intensities: a) moderate—50% 1RM; and b) high—70% 1RM; with a wash-out of 72 h between sessions].	-Affective/psychological responses, RPE^a^: ns, RPE^b^: ns, -Exercise performance responses, Maximal BPV_BP_: ^a^ns ^b^↓10%, Mean BPV_BP_: ^a^ns ^b^↓14%, T-REPS_BP_: ^ab^ns, T-VOL_BP_: ^ab^ns
	Crossover design			
	1: CON^a^			
	2: CON^b^			
	3: FFP2/N95^a^			
	4: FFP2/N95^b^			
Ryu and Jong-Geun , (2021)	RCT	Healthy adults., (*n* = 11; M = 11; W = 0), 23 ± 3 years	Incremental treadmill cardiopulmonary exercise test (Bruce’s standard protocol).	-Exercise performance responses, TTE: ns
	Crossover design			
	1: CON			
	2: SM			
	3: FFP2/N95			
Shaw et al. (2020)	RCT	Healthy and physically active adults., (*n* = 14; M = 7; W = 7), 28 ± 9 years	Incremental cycle ergometer exercise to exhaustion (starting at 35–100 W; increases of 35 W every 2 min until voluntary exhaustion; cadence of 70–75 rpm).	-Affective/psychological responses, RPE: ns, -Exercise performance responses, TTE: ns, PO_MAX_: ns
	Crossover design			
	1: CON			
	2: CM			
	3: SM			
Slimani et al. (2021)	RCT	Healthy adults., (*n* = 17; M = 9; W = 8), 18 years	Warm-up exercises for 15 min [light runs (4 min); arm circles, jumping jacks, high knees jog, and back kicking (2.5 min); stretching exercises (1.5 min); and 4 sets of each exercise—push-up, sit-up, and squat (30 s of work per 30 s of rest)].	-Affective/psychological responses, RPE: ↑27%
	Crossover design			
	1: CON			
	2: CM			
Steinhilber et al. (2022)	RCT	Healthy adults., (*n* = 39; M = 20; W = 19), 38 ± 14 years	Constant load cycle ergometer exercise until exhaustion [two different intensities according to HR: a) 130 bpm; and b) 150 bpm].	-Affective/psychological responses, RPE: ^ab^ns, Dyspnea: ^a^↑22%–35% ^b^↑29–35%, -Exercise performance responses, PO_MAX_: ^a^↓9% ^b^ns
	Crossover design			
	1: CON			
	2: CM			
	3: SM			
	4: FFP2/N95 + EV			
Tornero-Aguilera et al. (2021)	RCT	Recreational athletes., (*n* = 72; M = 45; W = 27), 28 ± 6 years	Outdoor track field running tests: a) 50 m; b) 400 m.	-Affective/psychological responses, RPE: ^a^ns ^b^↑7%, Subjective stress responses: ^a^ns ^b^↑18%, -Exercise performance responses, Sprint time: ^a^↑13% ^b^↑19%
	Crossover design			
	1: CON			
	2: SM			
Wong et al. (2020)	RCT	Healthy and physically active adults., (*n* = 23; M = 10; W = 13), 34 ± 11 years	Constant load treadmill walking for 6 min (4 km/h at 10° incline).	-Affective/psychological responses, RPE: ↑13%–20%
	Crossover design			
	1: CON			
	2: SM			
Yoshihara et al. (2021)	RCT	Healthy and physically active adults., (*n* = 12; M = 8; W = 4), 24 ± 3 years	Constant load treadmill walking/jogging for 1 h (speed increases of 0.5–1.0 mph every 2 min until voluntary exhaustion).	-Affective/psychological responses, RPE: ns, Dyspnea: ↑379%–761%, Thermal sensation: ns, Fatigue level: ns
	Crossover design			
	1: CON			
	2: CM			
	3: SM			
	4: FFP2/N95			
Zhang et al. (2021)	RCT	Healthy adults., (*n* = 71; M = 35; W = 36), 28 ± 8 years	Incremental cycle ergometer exercise to exhaustion (incremental power of 15–25 W/min until exhaustion calculated to let subjects finish the exercise load test between 8 and 12 min; cadence of 60 rpm).	-Affective/psychological responses, RPE: ↑19%, Dyspnea: ↑19, -Exercise performance responses, TTE: ns, PO_MAX_: ↓5%
	Crossover design			
	1: CON			
	2: SM			
Zimmerman et al. (1991)	RCT	Healthy adults., (*n* = 12; M = 12; W = 0), 21 (18–24) years	Constant load cycle ergometer exercise (280–350 W; constant rate).	-Functional responses, Hand grip strength: ns
	Crossover design			
	1: CON			
	2: SM			

RCT, randomized controlled trial; CON, control condition (non-fascial mask wearing); CM, clothing mask; SM, surgical mask; FFP2/N95, facepiece respirator type 2; MP, mouthpiece; EV, exhalation valve; a: lower exercise intensity condition; b: higher exercise intensity condition; n: sample size; M, men; W, women; ± standard deviation values; min, minute; rpm, revolutions per minute; mph, miles per hour; h, hour; km/h, kilometers per hour; m, meters; RM, maximum repetitions; sec, seconds; BP, bench press exercise; VO_2_max, maximal oxygen uptake; METs, metabolic equivalent of task; PO_MAX_, maximal power output; HR, heart rate; HR_MAX_, predicted maximum heart rate; VT, ventilatory threshold; 6 MWT, six-minute walk test; STS^1min^, one-min sit-to-stand test; ns, statistically non-significant; ud, unavailable data; ↓ statistically significant lesser compared to the condition without mask; ↑ statistically significant greater compared to the condition without mask; RPE, rate of perceived exertion; T-REP, total number of repetitions; T-VOL, total volume of repetitions; TTE, time-to-exhaustion; BPV, bar propulsive velocity.

Among the types of exercise, the participants performed on cycle ergometer (*n* = 14) (Zimmerman et al., 1991; Fikenzer et al., 2020; Lässing et al., 2020; Shaw et al., 2020; Ade et al., 2021; Doherty et al., 2021; Egger et al., 2021; Epstein et al., 2021; Mapelli et al., 2021; Zhang et al., 2021; Jesus et al., 2022; Ng et al., 2022; Otsuka et al., 2022; Steinhilber et al., 2022), walking in treadmill (*n* = 13) (Roberge et al., 2010; Roberge et al., 2012a; Roberge et al., 2012b; Chen et al., 2016; Kim et al., 2016; Wong et al., 2020; Bar-On et al., 2021; Driver et al., 2021; Fukushi et al., 2021; Kampert et al., 2021; Rojo-Tirado et al., 2021; Ryu and Jong-Geun, 2021; Yoshihara et al., 2021), outdoor track field running (*n* = 2) (Dantas et al., 2021; Tornero-Aguilera et al., 2021), high intensity interval training (*n* = 2) (Modena et al., 2021; Slimani et al., 2021), resistance exercise (*n* = 1) (Rosa et al., 2022), and other functional tasks (*n* = 5), such as vertical jump (*n* = 1) (Dantas et al., 2021), six-minute walk test (6 MWT) (Person et al., 2018; Cabanillas-Barea et al., 2022) and one-min sit-to-stand test (STS^1min^) (Reychler et al., 2021), and healthcare work tasks (Morris et al., 2020). Regarding the types of face mask, studies used CM (*n* = 13) (Shaw et al., 2020; Ade et al., 2021; Dantas et al., 2021; Doherty et al., 2021; Driver et al., 2021; Fukushi et al., 2021; Kampert et al., 2021; Modena et al., 2021; Reychler et al., 2021; Rojo-Tirado et al., 2021; Slimani et al., 2021; Yoshihara et al., 2021; Steinhilber et al., 2022), SM (*n* = 27) (Zimmerman et al., 1991; Roberge et al., 2012a; Roberge et al., 2012b; Person et al., 2018; Fikenzer et al., 2020; Lässing et al., 2020; Shaw et al., 2020; Wong et al., 2020; Ade et al., 2021; Bar-On et al., 2021; Doherty et al., 2021; Egger et al., 2021; Epstein et al., 2021; Fukushi et al., 2021; Mapelli et al., 2021; Modena et al., 2021; Reychler et al., 2021; Ryu and Jong-Geun, 2021; Tornero-Aguilera et al., 2021; Yoshihara et al., 2021; Zhang et al., 2021; Cabanillas-Barea et al., 2022; Jesus et al., 2022; Ng et al., 2022; Otsuka et al., 2022; Steinhilber et al., 2022), FFP2/N95 (*n* = 14) (Roberge et al., 2010; Chen et al., 2016; Kim et al., 2016; Fikenzer et al., 2020; Morris et al., 2020; Ade et al., 2021; Egger et al., 2021; Epstein et al., 2021; Kampert et al., 2021; Mapelli et al., 2021; Rojo-Tirado et al., 2021; Ryu and Jong-Geun, 2021; Yoshihara et al., 2021; Cabanillas-Barea et al., 2022; Rosa et al., 2022), and FFP2/N95 + EV (*n* = 5) (Roberge et al., 2010; Roberge et al., 2012a; Chen et al., 2016; Kim et al., 2016; Steinhilber et al., 2022). The effects of face mask wearing during exercise on affective/psychological parameters were assessed by some studies (*n* = 33) (Roberge et al., 2010; Roberge et al., 2012a; Roberge et al., 2012b; Chen et al., 2016; Kim et al., 2016; Person et al., 2018; Fikenzer et al., 2020; Lässing et al., 2020; Morris et al., 2020; Shaw et al., 2020; Wong et al., 2020; Ade et al., 2021; Bar-On et al., 2021; Dantas et al., 2021; Doherty et al., 2021; Driver et al., 2021; Egger et al., 2021; Epstein et al., 2021; Fukushi et al., 2021; Kampert et al., 2021; Mapelli et al., 2021; Modena et al., 2021; Reychler et al., 2021; Rojo-Tirado et al., 2021; Slimani et al., 2021; Tornero-Aguilera et al., 2021; Yoshihara et al., 2021; Zhang et al., 2021; Cabanillas-Barea et al., 2022; Ng et al., 2022; Otsuka et al., 2022; Rosa et al., 2022; Steinhilber et al., 2022), while others (*n* = 21) (Zimmerman et al., 1991; Person et al., 2018; Fikenzer et al., 2020; Ade et al., 2021; Dantas et al., 2021; Driver et al., 2021; Egger et al., 2021; Epstein et al., 2021; Kampert et al., 2021; Mapelli et al., 2021; Modena et al., 2021; Reychler et al., 2021; Rojo-Tirado et al., 2021; Ryu and Jong-Geun, 2021; Tornero-Aguilera et al., 2021; Zhang et al., 2021; Cabanillas-Barea et al., 2022; Jesus et al., 2022; Ng et al., 2022; Rosa et al., 2022; Steinhilber et al., 2022) assessed its effects on exercise performance responses.

### 3.3 Face mask wearing effects on affective/psychological responses

#### 3.3.1 Discomfort

**FIGURE 2:**
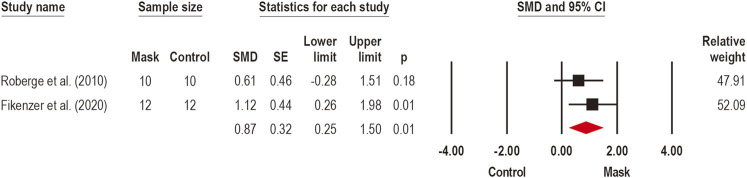
Acute effects of face mask vs. control (unmasked condition) on discomfort. Forest plots with pooled standardized mean difference (SMD), standard errors (SE), and 95% confidence intervals (CI) are displayed: (SMD: 0.87; 95% CI 0.25–1.5; *p* = 0.01; I^2^ = 0%).

Overall discomfort was assessed by four studies (Roberge et al., 2010; Fikenzer et al., 2020; Kampert et al., 2021; Reychler et al., 2021), during incremental cycle ergometer (Fikenzer et al., 2020) and treadmill (Kampert et al., 2021) exercises, constant walking on the treadmill (Roberge et al., 2010), and STS^1min^ (Reychler et al., 2021), using CM (Kampert et al., 2021; Reychler et al., 2021), SM (Fikenzer et al., 2020; Reychler et al., 2021), FFP2/N95 (Roberge et al., 2010; Fikenzer et al., 2020; Kampert et al., 2021), and FFP2/N95 + EV (Roberge et al., 2010) mask types. A meta-analysis was performed to estimate the effects for mask overall discomfort (*n* = 2) (Roberge et al., 2010; Fikenzer et al., 2020) (Figure 2). A large effect was observed for increased discomfort (SMD: 0.87; 95% CI 0.25 to 1.5; *p* = 0.01; I^2^ = 0%) with the use of face mask in exercise.

Thermal sensations and facial thermal discomfort during exercise were investigated by two (Kim et al., 2016; Yoshihara et al., 2021) and a single study (Morris et al., 2020), respectively. Thermal sensations were assessed during constant load treadmill walking/jogging for 1 h, using CM and SM (Yoshihara et al., 2021), FFP2/N95 (Kim et al., 2016; Yoshihara et al., 2021), and FFP2/N95 + EV (Kim et al., 2016). No effect was identified by the use of the face mask on thermal sensations. On the other hand, Morris et al. (2020) observed increased (22%) facial thermal discomfort with the use of FFP2/N95 during 45 min of light exercise simulating work in healthcare and related settings.

#### 3.3.2 Subjective stress responses

Subjective stress responses were investigated by a single study (Tornero-Aguilera et al., 2021) through a subjective perceived stress scale of 0–100 points to assess the degree to which situations in individual life are perceived as stressful (Cohen and Janicki-Deverts, 2012), measured after 50 m and 400 m outdoor track field running tests using SM. While the use of the face mask produced no stress responses in the 50 m test, the perceived stress was 18% higher compared to the control condition during the 400 m test. Therefore, although the evidence is limited, it is possible that the face mask may increase stress responses, although this effect may be dependent on the duration and intensity of the exercise.

#### 3.3.3 Affective responses

Affective responses (i.e., psychological manifestations selected for their ability to promote health, well-being and to solve recurrent adaptive problems) (Ekkekakis et al., 2005) were also investigated during exercise wearing a face mask by a single study (Dantas et al., 2021). Dantas et al. (2021) assessed affective responses by a feeling scale (11-point bipolar scale, composed of negative valences including: −5 = very bad, −3 = bad, −1 = reasonably bad; positive valences including: +5 = very good; +3 = good and +1 = reasonably good; and 0 being neutral) and showed lower affective responses (114%–148%) during outdoor track field running tests and countermovement jump using a CM by track and field athletes in comparison to non-masked condition. Although a single study reported lower affective responses with face mask wearing than a non-masked condition, the low number of studies limits further conclusion.

#### 3.3.4 Fatigue

Fatigue was investigated during exercise wearing a face mask by a single study (Yoshihara et al., 2021). Yoshihara et al. (2021) used CM, SM, and FFP2 masks during constant load treadmill walking or jogging for 1 h, and fatigue was measured through 10-point fatigue level scale (ranging from no fatigue at all to completely fatigued). Despite the lack of studies, no effect of face mask wearing was reported, suggesting that face mask use does not increase fatigue levels during exercise.

#### 3.3.5 Dyspnea

**FIGURE 3:**
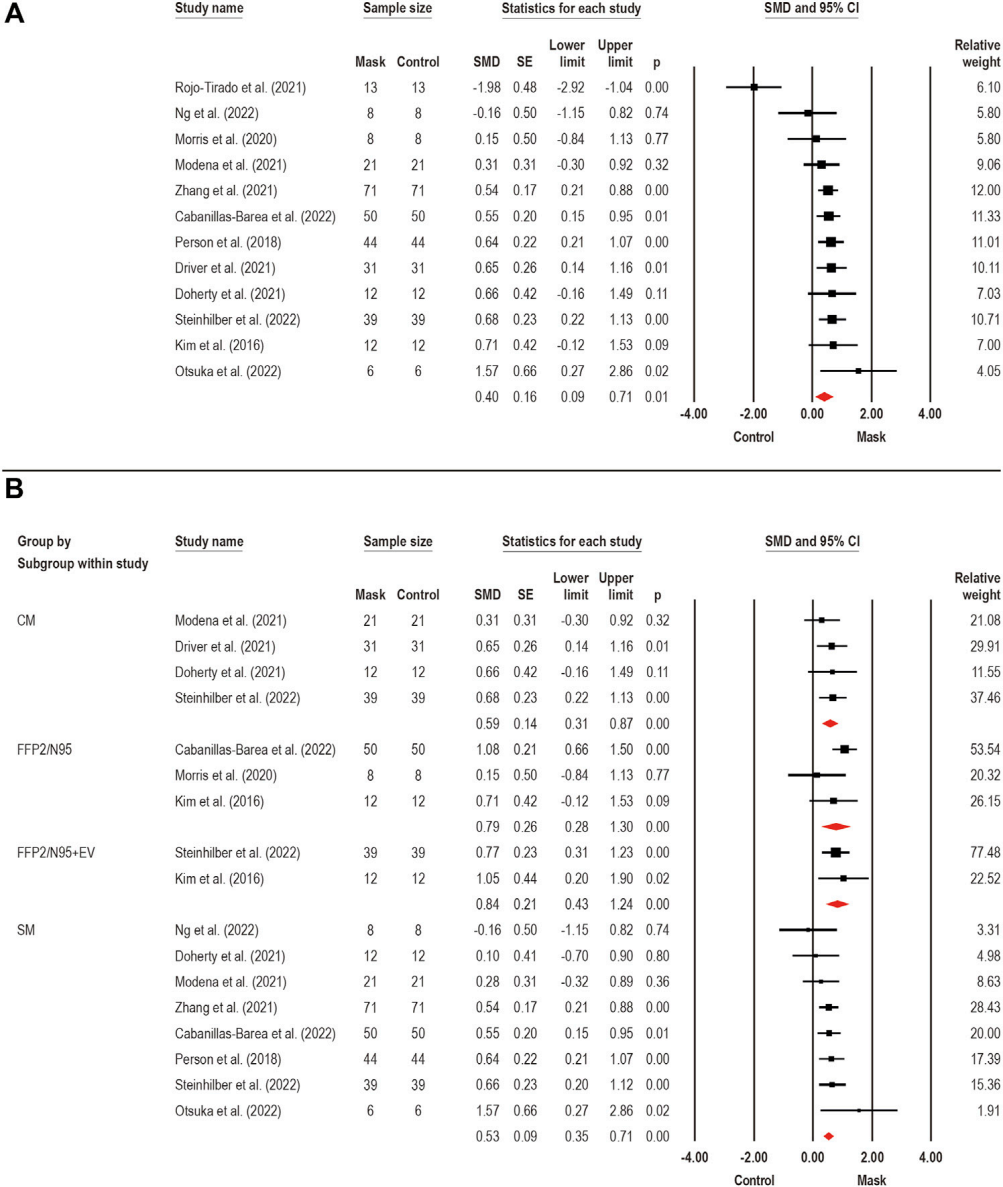
Acute effects of face mask vs. control (unmasked condition) on dyspnea. Forest plots with pooled standardized mean difference (SMD), standard errors (SE), and 95% confidence intervals (CI) are displayed: **(A)** analysis of face mask wearing effects on dyspnea (SMD: 0.40; 95% CI 0.09–0.71; *p* = 0.01; I^2^ = 68%); **(B)** subgroup analysis of face mask wearing effects by face mask type [CM: clothing mask (SMD: 0.59; 95% CI 0.31–0.87; *p* < 0.001; I^2^ = 0%); SM: surgical mask (SMD: 0.53; 95% CI 0.35–0.71; *p* < 0.001; I^2^ = 0%); FFP2/N95: facepiece respirator type 2 (SMD: 0.79; 95% CI 0.28–1.30; *p* < 0.001; I^2^ = 37%); and FFP2/N95 + EV: facepiece respirator type 2 with exhalation valve (SMD: 0.84; 95% CI 0.43–1.24; *p* < 0.001; I^2^ = 0%)].

Dyspnea (i.e., subjective experience of breathing discomfort and shortness of breath) (Reychler et al., 2021) was assessed by 16 studies. Twelve studies (Kim et al., 2016; Person et al., 2018; Morris et al., 2020; Doherty et al., 2021; Driver et al., 2021; Modena et al., 2021; Rojo-Tirado et al., 2021; Zhang et al., 2021; Cabanillas-Barea et al., 2022; Ng et al., 2022; Otsuka et al., 2022; Steinhilber et al., 2022) were pooled in a meta-analysis to estimate face mask wearing effects on dyspnea (Figure 3A). A moderate overall effect was detected by face mask on the dyspnea (SMD: 0.40; 95% CI 0.09 to 0.71; *p* = 0.01; I^2^ = 68%). Considering the existence of high heterogeneity, subgroup analyzes were performed for face mask type effects on dyspnea (Figure 3B): SM (*n* = 8) (Person et al., 2018; Doherty et al., 2021; Modena et al., 2021; Zhang et al., 2021; Cabanillas-Barea et al., 2022; Ng et al., 2022; Otsuka et al., 2022; Steinhilber et al., 2022), CM (*n* = 4) (Doherty et al., 2021; Driver et al., 2021; Modena et al., 2021; Steinhilber et al., 2022), FFP2/N95 (*n* = 3) (Kim et al., 2016; Morris et al., 2020; Cabanillas-Barea et al., 2022), and FFP2/N95 + EV (*n* = 2) (Kim et al., 2016; Steinhilber et al., 2022). The subgroup analysis showed a large effect for FFP2/N95 + EV (SMD: 0.84; 95% CI 0.43–1.24; *p* < 0.001; I^2^ = 0%) and FFP2/N95 (SMD: 0.79; 95% CI 0.28–1.30; *p* < 0.001; I^2^ = 37%), and moderate effect for CM (SMD: 0.59; 95% CI 0.31–0.87; *p* < 0.001; I^2^ = 0%) and SM (SMD: 0.53; 95% CI 0.35–0.71; *p* < 0.001; I^2^ = 0%). These findings suggest that face mask wearing can increase dyspnea sensations during exercise and the magnitude of this effect seems to depend on the type of face mask. In summary, mask type FFP2/N95 have a large effect size, while the CM and SM masks have a moderate effect size.

#### 3.3.6 Perceived exertion

**FIGURE 4:**
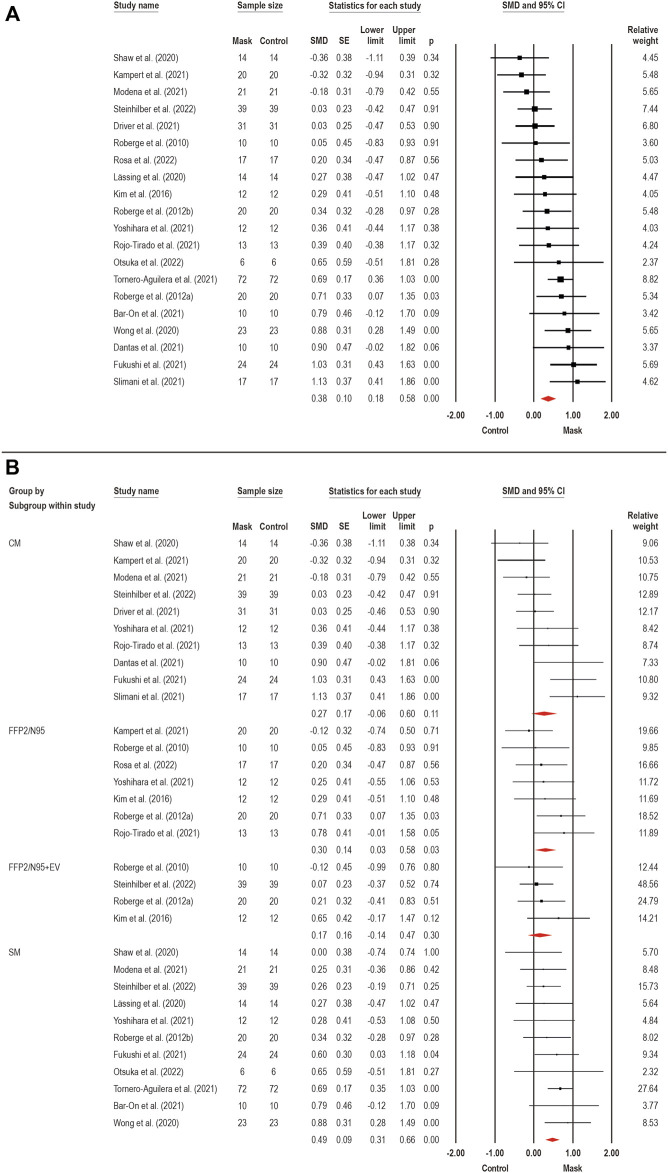
Acute effects of face mask vs. control (unmasked condition) on perceived exertion. Forest plots with pooled standardized mean difference (SMD), standard errors (SE), and 95% confidence intervals (CI) are displayed: **(A)** analysis of face mask wearing effects on perceived exertion (SMD: 0.38; 95% CI 0.18–0.58; *p* < 0.001; I^2^ = 46%); **(B)** subgroup analysis of face mask wearing effects by face mask type [CM: clothing mask (SMD: 0.27; 95% CI −0.06 to 0.60; *p* = 0.11; I^2^ = 62%); SM: surgical mask (SMD: 0.49; 95% CI 0.31–0.66; *p* < 0.001; I^2^ = 0%); FFP2/N95: facepiece respirator type 2 (SMD: 0.30; 95% CI 0.03–0.58; *p* = 0.03; I^2^ = 0%); and FFP2/N95 + EV: facepiece respirator type 2 with exhalation valve (SMD: 0.17; 95% CI −0.14 to 0.47; *p* = 0.30; I^2^ = 0%)].

Perceived exertion (i.e., an appropriate measure of internal training load) (Dantas et al., 2021) was assessed by 25 studies. A meta-analysis was performed to estimate the face mask wearing effects on perceived exertion (Figure 4A). A total of 20 studies pooled for an overall analysis, in which a small effect was observed for mask wearing on perceived exertion (SMD: 0.38; 95% CI 0.18–0.58; *p* < 0.001; I^2^ = 46%). Considering the existence of moderate heterogeneity, subgroup analyzes were performed for face mask type effects on perceived exertion (Figure 4B): SM (*n* = 11) (Roberge et al., 2012b; Lässing et al., 2020; Shaw et al., 2020; Wong et al., 2020; Bar-On et al., 2021; Fukushi et al., 2021; Modena et al., 2021; Tornero-Aguilera et al., 2021; Yoshihara et al., 2021; Otsuka et al., 2022; Steinhilber et al., 2022), CM (*n* = 10) (Shaw et al., 2020; Dantas et al., 2021; Driver et al., 2021; Fukushi et al., 2021; Kampert et al., 2021; Modena et al., 2021; Rojo-Tirado et al., 2021; Slimani et al., 2021; Yoshihara et al., 2021; Steinhilber et al., 2022), FFP2/N95 (*n* = 7) (Roberge et al., 2010; Roberge et al., 2012a; Kim et al., 2016; Kampert et al., 2021; Rojo-Tirado et al., 2021; Yoshihara et al., 2021; Rosa et al., 2022), and FFP2/N95 + EV (*n* = 4) (Roberge et al., 2010; Roberge et al., 2012a; Kim et al., 2016; Steinhilber et al., 2022). When analyzing the effects by the subgroups, we observed a moderate effect for SM (SMD: 0.49; 95% CI 0.31–0.66; *p* < 0.001; I^2^ = 0%), and small effect for FFP2/N95 (SMD: 0.30; 95% CI 0.03–0.58; *p* = 0.03; I^2^ = 0%), while no effects were found for CM (SMD: 0.27; 95% CI −0.06 to 0.60; *p* = 0.11; I^2^ = 62%) and FFP2/N95 + EV (SMD: 0.17; 95% CI −0.14 to 0.47; *p* = 0.30; I^2^ = 0%). Therefore, the face mask wearing effects on perceived exertion seems to depend on the type of face mask used, in which only the SM and FFP2/N95 mask types seem to increase perceived exertion during exercise.

### 3.4 Face mask wearing effects on exercise performance responses

#### 3.4.1 Time-to-exhaustion performance

**FIGURE 5:**
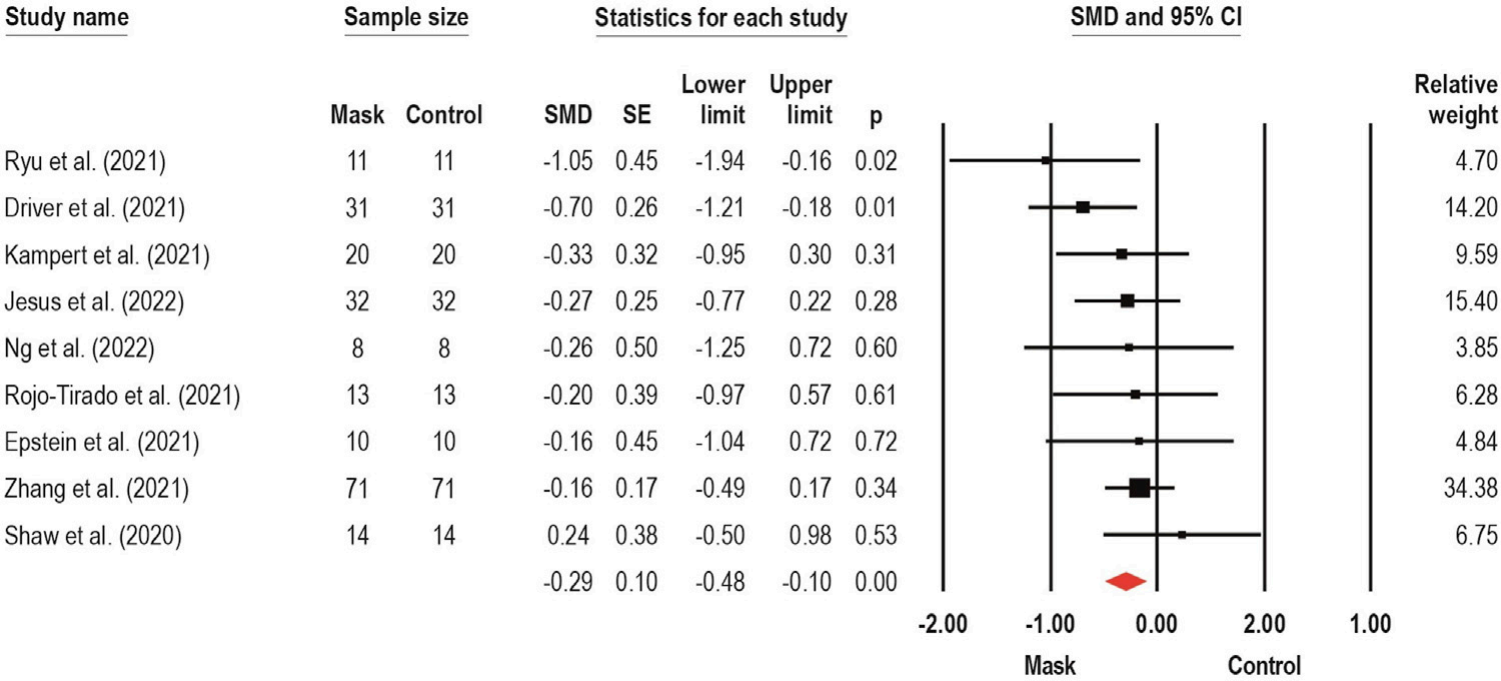
Acute effects of face mask vs. control (unmasked condition) on time-to-exhaustion performance. Forest plots with pooled standardized mean difference (SMD), standard errors (SE), and 95% confidence intervals (CI) are displayed: (SMD: −0.29; 95% CI −0.10 to −0.48; *p* < 0.001; I^2^ = 0%).

The TTE were assessed by nine trials (Shaw et al., 2020; Driver et al., 2021; Epstein et al., 2021; Kampert et al., 2021; Rojo-Tirado et al., 2021; Ryu and Jong-Geun, 2021; Zhang et al., 2021; Jesus et al., 2022; Ng et al., 2022) through submaximal (*n* = 1) (Jesus et al., 2022) and incremental (*n* = 4) (Shaw et al., 2020; Epstein et al., 2021; Zhang et al., 2021; Ng et al., 2022) exercise on cycle ergometer, and incremental exercise on treadmill (*n* = 4) (Driver et al., 2021; Kampert et al., 2021; Rojo-Tirado et al., 2021; Ryu and Jong-Geun, 2021), with CM (*n* = 4) (Shaw et al., 2020; Driver et al., 2021; Kampert et al., 2021; Rojo-Tirado et al., 2021), SM (*n* = 6) (Shaw et al., 2020; Epstein et al., 2021; Ryu and Jong-Geun, 2021; Zhang et al., 2021; Jesus et al., 2022; Ng et al., 2022), and FFP2/N95 (*n* = 4) (Epstein et al., 2021; Kampert et al., 2021; Rojo-Tirado et al., 2021; Ryu and Jong-Geun, 2021) face masks. A meta-analysis pooled nine studies (Shaw et al., 2020; Driver et al., 2021; Epstein et al., 2021; Kampert et al., 2021; Rojo-Tirado et al., 2021; Ryu and Jong-Geun, 2021; Zhang et al., 2021; Jesus et al., 2022; Ng et al., 2022) to estimate the overall effect of face mask wearing on TTE, which is displayed in Figure 5. We found a harmful effect of small magnitude on TTE with face mask use (SMD: −0.29; 95% CI −0.10 to −0.48; *p* < 0.001; I^2^ = 0%), suggesting that wear a face mask may reduce the exercise duration due to a shorter time to reach exhaustion.

#### 3.4.2 Power output performance

**FIGURE 6:**
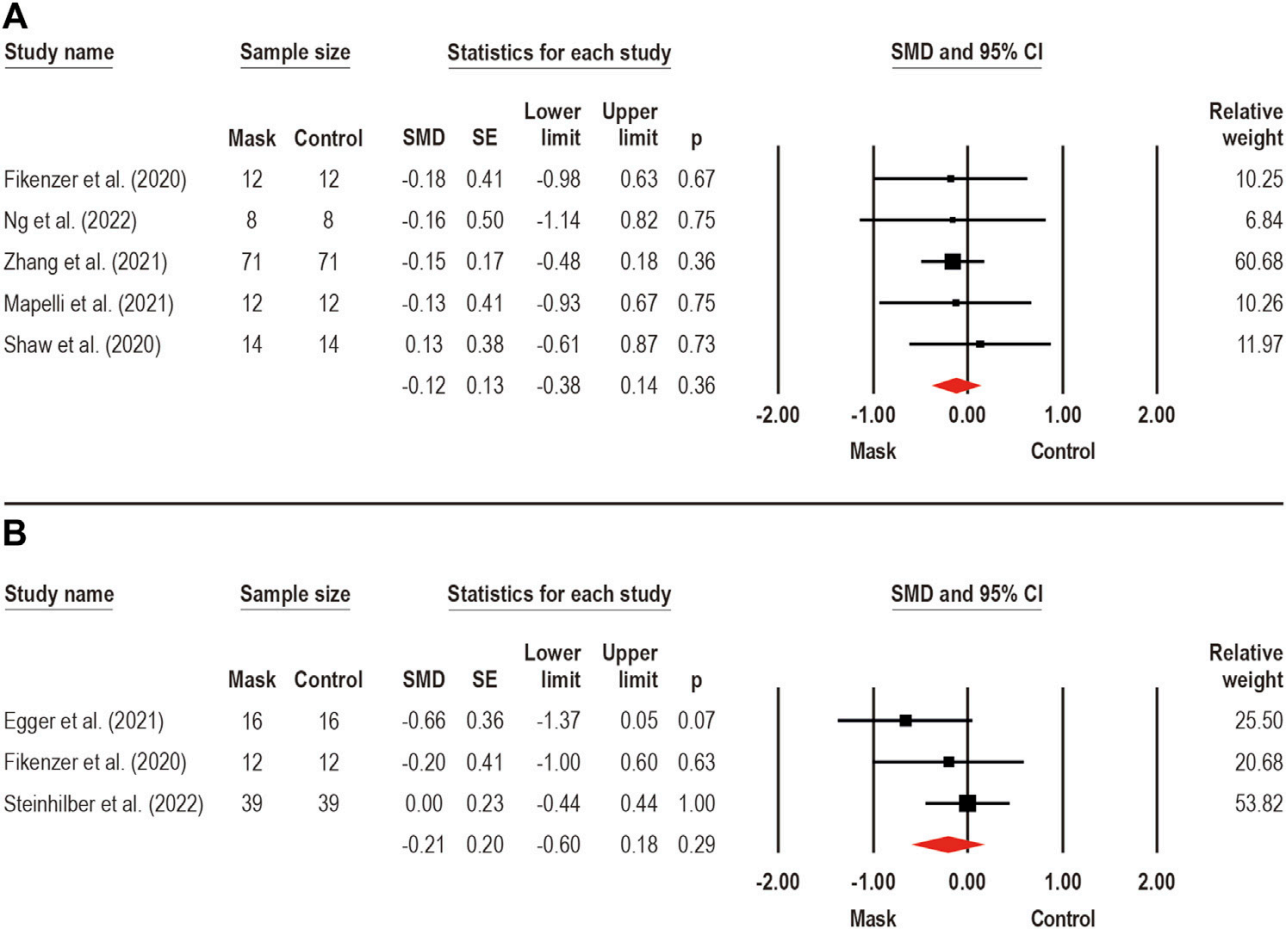
Acute effects of face mask vs. control (unmasked condition) on maximal power output (PO_MAX_) performance. Forest plots with pooled standardized mean difference (SMD), standard errors (SE), and 95% confidence intervals (CI) are displayed: **(A)** analysis of face mask wearing effects on PO_MAX_ by absolute values (W) (SMD: −0.12; 95% CI −0.38 to 0.14; *p* = 0.36; I^2^ = 0%); **(B)** analysis of face mask wearing effects on PO_MAX_ by body mass relative values (W/kg) (SMD: −0.21; 95% CI −0.60 to 0.18; *p* = 0.29; I^2^ = 17%).

The PO_MAX_ (i.e., highest power output achieved during the cycle ergometric test) were assessed by eight trials (Fikenzer et al., 2020; Shaw et al., 2020; Ade et al., 2021; Egger et al., 2021; Mapelli et al., 2021; Zhang et al., 2021; Ng et al., 2022; Steinhilber et al., 2022), respectively, through submaximal (*n* = 1) (Steinhilber et al., 2022) and incremental (*n* = 7) (Fikenzer et al., 2020; Shaw et al., 2020; Ade et al., 2021; Egger et al., 2021; Mapelli et al., 2021; Zhang et al., 2021; Ng et al., 2022) exercise on cycle ergometer, with CM (*n* = 3) (Shaw et al., 2020; Ade et al., 2021; Steinhilber et al., 2022), SM (*n* = 8) (Fikenzer et al., 2020; Shaw et al., 2020; Ade et al., 2021; Egger et al., 2021; Mapelli et al., 2021; Zhang et al., 2021; Ng et al., 2022; Steinhilber et al., 2022), and FFP2/N95 (*n* = 5) (Fikenzer et al., 2020; Ade et al., 2021; Egger et al., 2021; Mapelli et al., 2021; Steinhilber et al., 2022) face masks. Two meta-analyses estimate the face mask wearing effects on absolute (*n* = 5) (Fikenzer et al., 2020; Shaw et al., 2020; Mapelli et al., 2021; Zhang et al., 2021; Ng et al., 2022) and relative (*n* = 3) (Fikenzer et al., 2020; Egger et al., 2021; Steinhilber et al., 2022) PO_MAX_ (Figures 6A,B, respectively). No effects of face mask wearing were observed on both absolute (SMD: −0.12; 95% CI −0.38 to 0.14; *p* = 0.36; I^2^ = 0%) and relative (SMD: −0.21; 95% CI −0.60 to 0.18; *p* = 0.29; I^2^ = 17%) PO_MAX_.

Maximum and mean BPV were measured during the bench press exercise during face mask wearing (Rosa et al., 2022). After four sets of bench press at 50% and 70% of one-maximum repetition (RM) wearing an FFP2/N95, Rosa et al. (2022) observed lower maximum (−10%) and mean (−14%) BPV only during high-intensity exercise (i.e., 70% 1RM). Although limited to a single study, the negative effects of face mask on maximum and mean BPV appear to be intensity-dependent, suggesting that harmful effects caused by the use of the face mask only occur at higher intensities.

A single study (Dantas et al., 2021) evaluated jump performance through the maximum height reached in the countermovement jump using a CM. However, no effects were found on jump performance with the use of face mask.

#### 3.4.3 Muscle force and exercise performance of the total volume and maximum number of repetitions

The effects of face mask use on muscle force were also investigated (*n* = 1) (Zimmerman et al., 1991). Zimmerman et al. (1991) assessed muscle force through hand grip strength after constant load cycle ergometer exercise wearing SM, and no effects of face mask use were detected.

The exercise total volume (*n* = 1) and the maximum number of repetitions (*n* = 2) were also assessed. Rosa et al. (2022) assessed total volume and the maximum number of repetitions during four sets of bench press exercise, at intensities of 50% and 70% of 1RM, wearing FFP2/N95, and found no effects of face mask use. Reychler et al. (2021) assessed the performance of the STS^1min^ with the use of CM and SM and reported no effects of face mask use. Thus, the use of a face mask does not seem to influence the performance of the total volume and the maximum number of repetitions.

#### 3.4.4 Walking and running tests’ performance

**FIGURE 7:**
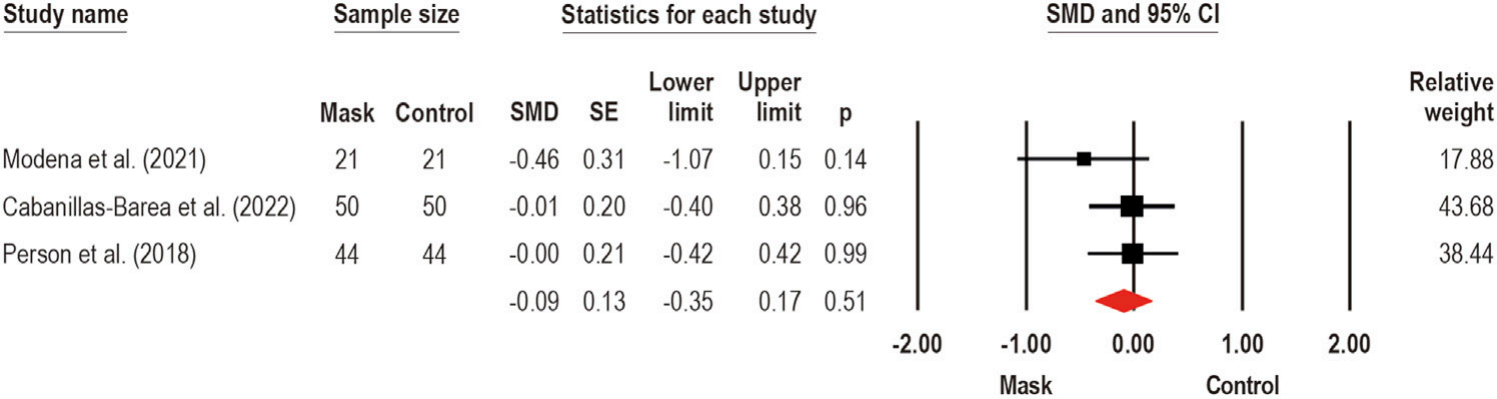
Acute effects of face mask vs. control (unmasked condition) on the distance traveled performance during walking and running functional tests. Forest plots with pooled standardized mean difference (SMD), standard errors (SE), and 95% confidence intervals (CI) are displayed: (SMD: −0.09; 95% CI −0.35 to 0.17; *p* = 0.51; I^2^ = 0%).

Three studies (Person et al., 2018; Modena et al., 2021; Cabanillas-Barea et al., 2022) investigated the face mask wearing effects on distance traveled (i.e., total distance achieved during testing) in walking and running tests, such as 6 MWT (Person et al., 2018; Cabanillas-Barea et al., 2022) and YoYo-Intermittent Recovery Test (YYIRT) (Modena et al., 2021), using CM (Modena et al., 2021), SM (Person et al., 2018; Modena et al., 2021; Cabanillas-Barea et al., 2022), and FFP2/N95 (Cabanillas-Barea et al., 2022). A meta-analysis pooling three studies (Person et al., 2018; Modena et al., 2021; Cabanillas-Barea et al., 2022) to estimate the face mask wearing effects on distance traveled during walking and running tests (SMD: −0.09; 95% CI −0.35 to 0.17; *p* = 0.51; I^2^ = 0%), as can be seen in Figure 7. No significant adverse effects were detected for face mask wearing on distance traveled assessed during walking and running tests.

Two studies (Dantas et al., 2021; Tornero-Aguilera et al., 2021) investigated the impact of face mask use on sprint time at 30 m (Dantas et al., 2021), 50 m (Tornero-Aguilera et al., 2021), and 400 m (Tornero-Aguilera et al., 2021), with the use of CM (Dantas et al., 2021) and SM (Tornero-Aguilera et al., 2021) mask types. While Dantas et al. (2021) found no effects caused by the face mask wearing, Tornero-Aguilera et al. (2021) observed greater sprint times in 50 m (13%) and 400 m (18%) compared to unmasked condition.

Acceleration was also measured during the sprint. A single study (Dantas et al., 2021) evaluated sprint acceleration during five maximum 30 m sprints using a CM. However, no negative effects on sprint acceleration were found with the use of a face mask.

### 3.5 Studies’ quality appraisal

In most studies (*n* = 36), a visual analysis showed an overall risk of “some concerns.” Most studies did not describe the randomization process (e.g., randomization, software, and others), allocation concealment (use of envelopes), and there was no pre-specified analysis plan available, so it was unclear whether the reported analyzes were pre-specified. Only few studies (*n* = 6) had a protocol record in clinical trials, however, it did not describe the pre-analyses. In general, a single study was judged as “low risk of bias” (Shaw et al., 2020). For the affective/psychological outcomes such as overall discomfort (*n* = 4), thermal sensations (*n* = 2), facial thermal discomfort (*n* = 1), stress (*n* = 1) and affective responses (*n* = 1), fatigue (*n* = 1), dyspnea (*n* = 16), and perceived exertion (*n* = 24) the overall risk was judged as “some concerns.” In exercise performance outcomes, TTE (*n* = 9), PO_MAX_ (*n* = 8), BPV (*n* = 1), jump height (*n* = 1), the maximum number of repetitions (*n* = 2), muscle strength (*n* = 1), distance traveled (*n* = 3), sprint time (*n* = 2) and acceleration (*n* = 1) were assessed and all the studies’ overall risk were judged as “some concern.” Full details of the risk of bias assessment are presented in the Supplementary Material.

## 4 Discussion

To the best of our knowledge, this study presents the first synthesis of available evidence on the acute effects of face mask use during exercise on affective/psychological and exercise performance responses exclusively in healthy adults of different/diverse training status. We found that the face mask wearing during exercise increases discomfort, dyspnea, and perceived exertion (especially wearing SM and FFP2/N95 mask types). Furthermore, face mask wearing can reduce the TTE during treadmill and cycle ergometer tests, without affecting PO_MAX_ and distance traveled in functional tests.

Previously, studies have found adverse effects of face mask wearing on overall (3%–150%) (Fikenzer et al., 2020; Kampert et al., 2021; Reychler et al., 2021) and facial thermal (22%) (Morris et al., 2020) discomforts during exercise. Our results showed a large negative effect of face mask use on discomfort levels, compared to no-mask condition. Adverse effects were also observed on subjective stress responses (18%) (Tornero-Aguilera et al., 2021) and affective responses (114%–148%) (Dantas et al., 2021). Regarding the stress and affective responses, although there seems to be an effect of the mask on these parameters, the number of studies is still small. Discomfort caused by face mask wearing can be explained by some factors such as inadequate respiratory gas exchange (Tornero-Aguilera et al., 2021), reduced ventilatory capacity (Engeroff et al., 2021), and changes in the mask’s microenvironment caused by sweat and water vapor retention, which causes even more breathing resistance and discomfort sensations (Yoshihara et al., 2021), and can lead to a stressful event in individuals (Andre et al., 2018) by symptoms such as tightness, suffocation, and claustrophobia in those who use it (Driver et al., 2021).

Dyspnea represents a subjective experience of breathing discomfort which consists of qualitatively distinct sensations that vary according to the exercise intensity (Reychler et al., 2021). Through our meta-analysis, we found a moderate adverse effect of exercise with a face mask on dyspnea. However, high heterogeneity values were observed and explored. A subgroup analysis showed that effects induced by the face mask use during exercise seems to depend on the type of mask used, since FFP2/N95 type (with and without exhalation valve) have greater negative effects on dyspnea than CM and SM types. Our findings confirm and strengthen the existing evidence and the reported moderate effect by a previous meta-analytical review (Shaw et al., 2021), indicating that face mask wearing during exercise causes an increase in dyspnea. The respiratory resistance caused by the mask seems to cause increased dyspnea level at the clinically relevant threshold (Reychler et al., 2021).

The perceived exertion has been constantly assessed during face mask wearing on different exercises (Ade et al., 2021; Driver et al., 2021; Slimani et al., 2021; Tornero-Aguilera et al., 2021; Rosa et al., 2022). Although the literature shows contradictory results, a recent meta-analysis (Shaw et al., 2021) showed increased perceived exertion of moderate and small effects for SM and FFP2/N95 face masks, respectively. In our study, we showed a small effect of face mask use on perceived exertion. However, a moderate magnitude heterogeneity was identified and explored. A subgroup analysis indicated that the increased perceived exertion only occurs during SM and FFP2/N95 mask-wearing, which demonstrate increases with moderate and small effect sizes, respectively. Airflow resistance is a key element of the face mask function (Hopkins et al., 2021), and although face masks offer different levels of breathing restriction, the observed effect on perceived exertion may be more related to the covered no-mask condition, as several studies (Lässing et al., 2020; Driver et al., 2021; Egger et al., 2021) found no effects of face mask wearing during cardiopulmonary exercise testing associated with spirometry equipment (i.e., mouthpiece and tubing), which can promote increased breathing resistance similar to FFP2/N95 respirators (Hopkins et al., 2021). Therefore, the simple fact of using the cardiopulmonary test equipment may have been enough to cause discomfort and perceived exertion in both masked and unmasked conditions in a similar response, and so it is possible that the face mask exclusive effects can be mitigated. Many studies (Motoyama et al., 2016; Andre et al., 2018; Dantas et al., 2021; Slimani et al., 2021; Tornero-Aguilera et al., 2021) that found greater perceived exertion wearing a face mask have been performed exercises at higher intensity levels associated with activities that involved multiple muscle groups, and in its control condition, the face was fully uncovered, as in non-laboratory tests. Therefore, the non-spirometry tests, such as outdoor track field running tests (Dantas et al., 2021; Tornero-Aguilera et al., 2021), may be more likely to show the exclusive effects of face mask use on perceived exertion.

As the exercise perceived exertion seems to increase when using a face mask, it would be expected that the TTE could be reduced with its use. Although the literature shows contradictory results, one of the studies (Jesus et al., 2022) that showed a decreased (-10%) TTE with the face mask use observed this effect during severe exercise intensity (i.e., 25% above ventilatory threshold) but not at moderate exercise intensity (i.e., 25% below ventilatory threshold), which suggests that the face mask use effect on TTE seem to be dependent on the exertion intensity, once this effect may be perceptible only during high-intensity exercise. In our study, we reveal decreased TTE with face mask use, showing that face mask wearing can produce a small negative effect on the performance of TTE. Psychophysiological factors may explain these detrimental effects on TTE. As mentioned previously, discomfort, stress responses, and perceived exertion are some of the main reasons that justify the observed impairments (Tornero-Aguilera et al., 2021). Previously, a large inverse correlation (*r* = −0.73, *p* = 0.020) between dyspnea and TTE (Boyle et al., 2022), added to that, studies have already reported lesser ventilation with face mask wearing (Fikenzer et al., 2020; Egger et al., 2021; Umutlu et al., 2021), once higher-intensity exercise (e.g., TTE) necessitates higher ventilation (Hopkins et al., 2021), and therefore, the greater the exertion intensity, greater are the impact of the face mask wearing (Engeroff et al., 2021). Face mask wearing negative effects were also observed in other physiological parameters, such as arterial oxygen saturation (Kampert et al., 2021; Romero-Arenas et al., 2021; Tornero-Aguilera et al., 2021) and oxygen uptake (Fikenzer et al., 2020; Driver et al., 2021; Egger et al., 2021), that also been reported and confirmed by recent meta-analyses (Engeroff et al., 2021). Thus, together, the negative responses of psychophysiological parameters could justify the reduction of TTE performance with face mask wearing.

Exercise performance parameters such as PO could also be affected by face mask wearing. The PO_MAX_ depends on energy consumption and the maximum oxygen uptake (Fikenzer et al., 2020), and as face mask wearing appears to decrease oxygen uptake (Engeroff et al., 2021), the reduction in PO_MAX_ seems to be mainly related to negative effects on respiratory function (Fikenzer et al., 2020). Increases in the respiratory muscles’ work and competition for blood supply between these respiratory muscles and exercising muscles can also help to explain the observed decreases (Romero-Arenas et al., 2021). However, the decreased PO_MAX_ (4%–9%) (Fikenzer et al., 2020; Egger et al., 2021; Zhang et al., 2021; Steinhilber et al., 2022) may be minimal effects that may be negligible, once our meta-analyses have shown that these detrimental effects are not significant when considering both absolute (Watts) and relative (Watts/kg) PO_MAX_. Nevertheless, these findings should be analyzed with caution due to the number of studies that are still small and do not allow analysis by different mask types.

Maximum and mean BPV collected during the ascending portion of a given movement are widely used to assess sports performance due to the force-velocity relationship (Loturco et al., 2015). A single study found detrimental effects on maximum and mean (Rosa et al., 2022) BPV during high-intensity bench press exercise using a face mask. However, authors observed no effects during low-intensity bench press exercise. The lower BPV observed only during the high-intensity exercise suggests greater fatigue induced by the face mask compared to low-intensity exercise (Rosa et al., 2022). Previously, Jagim et al. (2018) reported decreased BVP (2%–4%) during bench press exercise with the use of elevation training mask. Deleterious effects such as the fatigue (Rosa et al., 2022) associated to the reduced use of fast-twitch muscle fibers (Jagim et al., 2018) could explain the lower BPV. Fast-twitch muscle fibers produce the greatest response to lactate due to their greater dependence on the anaerobic glycolytic system (Jagim et al., 2018). Therefore, decreased blood lactate levels as observed by Jagim et al. (2018) and Motoyama et al. (2016), could indicate the reduced levels of recruitment of fast-twitch fibers, causing slower movements. Although some evidence points to a possible reduction in BPV with the use of the face mask, these effects must be interpreted with caution due to the low number of studies. High methodological quality clinical trials should be developed to better understand the acute effects of face mask use on BPV, which is an important parameter of muscle performance (Loturco et al., 2015).

The vertical jump, considered as another muscle performance parameter, was assessed by a single study (Dantas et al., 2021), which found no effects of face mask use on countermovement jump maximum height. Although there seem to be no negative effects of face mask use on jumping performance, the lack of studies does not allow us to fully understand the effects of face mask use on jumping performance. Future studies should investigate the effects of facemask use in consecutive jump protocols, which may be more susceptible to showing possible face mask harmful effects.

Beyond the force-velocity relationship, face mask use could also impair the muscle force production. Previously, studies showed impaired knee extensors isometric strength (da Silva et al., 2022) and the maximum number of repetitions in the squat (Andre et al., 2018), leg press (Andre et al., 2018), and bench press (Motoyama et al., 2016) exercises, when using another kind of face mask (i.e., elevation training mask). Even though the literature shows small evidence of adverse effects produced by the use of elevation training mask, limited evidence suggests that face mask use does not affect these parameters. A single study (Zimmerman et al., 1991) tested the handgrip strength after a constant load cycle ergometer exercise wearing SM and found no effects. However, it is possible that the face mask wearing effects on muscle force are not perceptible due to the lack of specificity between the way in which the muscle force was assessed and the type of performed exercise. Furthermore, we observed no harmful effects of face mask use on the maximum number of repetitions in the bench press exercise (Rosa et al., 2022) and STS^1min^ (Reychler et al., 2021), using CM and SM (Reychler et al., 2021) and FFP2/N95 (Rosa et al., 2022). Despite this, the available evidence does not allow us to state that the use of a face mask does not affect the muscle force and the maximum number of repetitions, and thus, more studies are needed to understand the face mask wearing effects on muscle force production performance.

The performance of the walking and running tests such as 6 MWT (Person et al., 2018; Cabanillas-Barea et al., 2022), YYIRT (Modena et al., 2021), and outdoor track field running (Dantas et al., 2021; Tornero-Aguilera et al., 2021) were also investigated. The distance traveled was assessed during the 6 MWT (Person et al., 2018; Cabanillas-Barea et al., 2022) and YYIRT (Modena et al., 2021). While no effects of face mask use were identified during the 6 MWT (Person et al., 2018; Cabanillas-Barea et al., 2022), shorter distances (11%–13%) were achieved in YYIRT (Modena et al., 2021). However, our meta-analysis showed that these effects are not significant, suggesting that face mask use does not negatively affect the performance of both tests. Despite the absence of negative effects, our findings must be interpreted with caution, since the distance covered was evaluated by tests with different characteristics, which could respond differently to mask use due to different exercise intensities. The 6 MWT is a test in which participants had to walk as fast as possible without running for 6 min (Cabanillas-Barea et al., 2022), on the other hand, YYIRT is a running incremental exercise used both to simulate high-intensity exercise and to assess specific aerobic fitness related to team sports performance (Modena et al., 2021).

For the effects of the face mask on sprint performance, studies investigated the face mask wearing effects on sprint time (Dantas et al., 2021; Tornero-Aguilera et al., 2021) and acceleration (Dantas et al., 2021). Sprint time was assessed during 30 m (Dantas et al., 2021), 50 m (Tornero-Aguilera et al., 2021), and 400 m (Tornero-Aguilera et al., 2021), using CM (Dantas et al., 2021) and SM (Tornero-Aguilera et al., 2021). While Dantas et al. (2021) found no face mask wearing negative effects in 30 m sprints, Tornero-Aguilera et al. (2021) observed greater sprint times (13%–18%) at distances from 50 m compared to control condition. Increased sprint time could be explained by potential metabolic changes and decrease in muscle efficiency (consequence of impaired autonomic stability), lower cardiac fitness, and decreased muscle blood supplies (Tornero-Aguilera et al., 2021). Although the available evidence is limited to two studies, the use of the face mask seems to be able to produce negative effects on the sprint time at distances from 50 m, however, the evidence does not allow us to understand if these effects are caused by the different sprint distances or by the different face mask used between the studies. Regarding acceleration, (Dantas et al., 2021), found no effects of face mask use during five maximum 30 m sprints wearing a CM. Nonetheless, this evidence is insufficient to state that face mask use does not affect sprint acceleration. Therefore, high methodological quality clinical trials are needed to understand the real effects of face mask use on sprint performance.

Our study has some limitations. First, we focused on investigating the effects of a face mask wearing on apparently healthy adults. Therefore, we cannot assert that our findings can be applied to other populations, such as children and the elderly or people with clinical conditions. Studies showed some variability in face mask type, exercise modality, and exertion intensity. However, the low number of studies did not allow us to explore these data further to better understand the real effects of face mask use in each condition. Some studies (Lässing et al., 2020; Driver et al., 2021; Egger et al., 2021) have assessed psychophysiological responses from cardiopulmonary tests using spirometry equipment over the face mask. Covering the face even without the combination with the face mask can promote adverse effects and cause discomfort and greater perceived exertion. Therefore, one way to avoid this bias would be to perform high-intensity exercises (e.g., outdoor track field running tests) with a control condition without any kind of device that covers the face. In this same perspective, most studies that assessed the impact of the face mask are based on laboratory protocols, whose practical applicability is questionable (Dantas et al., 2021). Nonetheless, functional tests can provide clearer information about the real effects of the face mask during exercise. Fourth, most studies (*n* = 26) did not determine the sample size through a sample size calculation and our analysis is on the risk of type I error. Further high methodological quality studies should explore the influence of each mask type in different intensities and types of exercise, in addition to exploring the long-term effects of face mask use. Studies found in pre-print databases have not usually been peer-reviewed. Although we included preprint databases in our search strategy, no non-peer-reviewed studies were found and included in our review. Lastly, due to the small number of studies, we could not perform publication bias tests.

Our study has several implications for exercise practice. In summary, we indicate that face mask using negatively affects affective/psychological responses such as discomfort, dyspnea, and perceived exertion, and can reduce exercise time at high intensities, but does not produce harmful effects on distance traveled in walking and running functional tests. Nevertheless, it is well documented that exercise works against several chronic diseases, and is strongly associated with reduced risk for severe COVID-19 outcomes (Sallis et al., 2021), and thus, although face mask causes impairments in some affective/psychological and exercise performance aspects during exercise, they are well-established protective measures against airborne infectious diseases (e.g., COVID-19), especially when combined with other preventive measures such as ventilation and distancing, which together can reduce viral concentrations in the environment and increase the protective effectiveness of face masks to contain viral transmission (Liang et al., 2020; Cheng et al., 2021). Apparently, the face masks that offer the greater protection levels (e.g., SM or FFP2/N95) may be the same ones that produce the greatest negative impacts on exercise (Engeroff et al., 2021). However, they may be recommended for indoor and/or group activities, especially when the environment is poorly ventilated (Cheng et al., 2021) or adequate physical distancing cannot be maintained, such as in gyms or training centers (Shurlock et al., 2021). Based on the study data, we do not recommend the abandonment of masks when exercising, but we recommend that affective/psychological and exercise performance responses may be influenced by the face mask use and some adaptations to the intensity of exercise may be required for those exercising for health.

## 5 Conclusion

Based on the available evidence, face mask wearing during exercise increases discomfort (large effect), dyspnea (moderate effect), and perceived exertion (small effect). Moreover, face mask use can reduce the TTE performance (small effect), without effects on cycle ergometer PO_MAX_ and distance traveled in walking and running functional tests. However, some aspects may be dependent on the face mask type, as the increased dyspnea (large effect for FFP2/N95 + EV and FFP2/N95; moderate effect for CM and SM) and perceived exertion (moderate and small effects for SM and FFP2/N95, respectively).

## Data Availability

The datasets presented in this study can be found in online repositories. The names of the repository/repositories and accession number(s) can be found in the article/Supplementary Material.

## Supplementary material

SUPPLEMENTARY FIGURE S1 Risk of bias rating for discomfort, stress and affective responses, and fatigue outcomes, displayed as traffic light plots. The colours indicate high (red), unclear (yellow) or low (green) risk for the respective bias domain/item. D1: randomization process; DS: bias arising from period and carryover effects; D2: deviations from the intended interventions; D3: missing outcome data; D4: measurement of the outcome; D5: selection of the reported result.

SUPPLEMENTARY FIGURE S2 Risk of bias rating for dyspnea outcome, displayed as traffic light plots. The colours indicate high (red), unclear (yellow) or low (green) risk for the respective bias domain/item. D1: randomization process; DS: bias arising from period and carryover effects; D2: deviations from the intended interventions; D3: missing outcome data; D4: measurement of the outcome; D5: selection of the reported result.

SUPPLEMENTARY FIGURE S3 Risk of bias rating for perceived exertion outcome, displayed as traffic light plots. The colours indicate high (red), unclear (yellow) or low (green) risk for the respective bias domain/item. D1: randomization process; DS: bias arising from period and carryover effects; D2: deviations from the intended interventions; D3: missing outcome data; D4: measurement of the outcome; D5: selection of the reported result.

SUPPLEMENTARY FIGURE S4 Risk of bias rating for time-to-exhaustion (TTE) performance outcome, displayed as traffic light plots. The colours indicate high (red), unclear (yellow) or low (green) risk for the respective bias domain/item. D1: randomization process; DS: bias arising from period and carryover effects; D2: deviations from the intended interventions; D3: missing outcome data; D4: measurement of the outcome; D5: selection of the reported result.

SUPPLEMENTARY FIGURE S5 Risk of bias rating for maximal power output (POMAX) performance outcome, displayed as traffic light plots. The colours indicate high (red), unclear (yellow) or low (green) risk for the respective bias domain/item. D1: randomization process; DS: bias arising from period and carryover effects; D2: deviations from the intended interventions; D3: missing outcome data; D4: measurement of the outcome; D5: selection of the reported result.

SUPPLEMENTARY FIGURE S6 Risk of bias rating for performance of the maximum number of repetitions, muscle strength, bar propulsive velocity, and jump height outcomes, displayed as traffic light plots. The colours indicate high (red), unclear (yellow) or low (green) risk for the respective bias domain/item. D1: randomization process; DS: bias arising from period and carryover effects; D2: deviations from the intended interventions; D3: missing outcome data; D4: measurement of the outcome; D5: selection of the reported result.

SUPPLEMENTARY FIGURE S7 Risk of bias rating for distance traveled performance, sprint acceleration, and sprint time outcomes, displayed as traffic light plots. The colours indicate high (red), unclear (yellow) or low (green) risk for the respective bias domain/item. D1: randomization process; DS: bias arising from period and carryover effects; D2: deviations from the intended interventions; D3: missing outcome data; D4: measurement of the outcome; D5: selection of the reported result.

## References

[B1] Ade C. J. Turpin V. R. G. Parr S. K. Hammond S. T. White Z. Weber R. E. (2021). Does wearing a facemask decrease arterial blood oxygenation and impair exercise tolerance? Respir. Physiol. Neurobiol. 294, 103765. 10.1016/j.resp.2021.103765 34352384 PMC9715989

[B2] Andre T. L. Gann J. J. Hwang P. S. Ziperman E. Magnussen M. J. Willoughby D. S. (2018). Restrictive breathing mask reduces repetitions to failure during a session of lower-body resistance exercise. J. Strength Cond. Res. 32, 2103–2108. 10.1519/JSC.0000000000002648 29847532

[B3] Andreu J. P. (2021). El uso de mascarilla en el deporte: Una revisión integradora durante la COVID-19. Cultura. Cienc. Deporte 16.

[B4] Aquino E. M. L. Silveira I. H. Pescarini J. M. Aquino R. Souza-Filho J. A. D. Rocha A. D. S. (2020). Social distancing measures to control the COVID-19 pandemic: Potential impacts and challenges in Brazil. Cien. Saude Colet. 25, 2423–2446. 10.1590/1413-81232020256.1.10502020 32520287

[B5] Asadi S. Cappa C. D. Barreda S. Wexler A. S. Bouvier N. M. Ristenpart W. D. (2020). Efficacy of masks and face coverings in controlling outward aerosol particle emission from expiratory activities. Sci. Rep. 10, 15665. 10.1038/s41598-020-72798-7 32973285 PMC7518250

[B6] Asadi-Pooya A. A. Cross J. H. (2020). Is wearing a face mask safe for people with epilepsy? Acta Neurol. Scand. 142, 314–316. 10.1111/ane.13316 32654134 PMC7405123

[B7] Bar-On O. Gendler Y. Stafler P. Levine H. Steuer G. Shmueli E. (2021). Effects of wearing facemasks during brisk walks: A COVID-19 dilemma. J. Am. Board Fam. Med. 34, 798–801. 10.3122/jabfm.2021.04.200559 34312270

[B8] Boyle K. G. Napoleone G. Ramsook A. H. Mitchell R. A. Guenette J. A. (2022). Effects of the Elevation Training Mask® 2.0 on dyspnea and respiratory muscle mechanics, electromyography, and fatigue during exhaustive cycling in healthy humans. J. Sci. Med. Sport 25, 167–172. 10.1016/j.jsams.2021.08.022 34538564

[B9] Cabanillas-Barea S. Rodríguez-Sanz J. Carrasco-Uribarren A. López-de-Celis C. González-Rueda V. Zegarra-Chávez D. (2022). Effects of using the surgical mask and FFP2 during the 6-min walking test. A randomized controlled trial. Int. J. Environ. Res. Public Health 18, 12420. 10.3390/ijerph182312420 PMC8656790 34886145

[B10] Chen Y. Yang Z. Wang J. Gong H. (2016). Physiological and subjective responses to breathing resistance of N95 filtering facepiece respirators in still-sitting and walking. Int. J. Ind. Ergon. 53, 93–101. 10.1016/j.ergon.2015.11.002

[B11] Cheng Y. Ma N. Witt C. Rapp S. Wild P. S. Andreae M. O. (2021). Face masks effectively limit the probability of SARS-CoV-2 transmission. Science 372, 1439–1443. Online ahead of print. 10.1126/science.abg6296 PMC8168616 34016743

[B12] Chu D. K. Akl E. A. Duda S. Solo K. Yaacoub S. Schünemann H. J. (2020). Physical distancing, face masks, and eye protection to prevent person-to-person transmission of SARS-CoV-2 and COVID-19: A systematic review and meta-analysis. Lancet 395, 1973–1987. 10.1016/S0140-6736(20)31142-9 32497510 PMC7263814

[B13] Cohen J. (1988). Statistical power analysis for the behavioral sciences. Lawrence Erlbaum Associates.

[B14] Cohen S. Janicki-Deverts D. (2012). Who's stressed? Distributions of psychological stress in the United States in probability samples from 1983, 2006, and 20091. J. Appl. Soc. Psychol. 42, 1320–1334. 10.1111/j.1559-1816.2012.00900.x

[B15] da Silva K. J. da Silva L. C. Felippe L. C. Silva-Cavalcante M. D. Franco-Alvarenga P. E. Learsi S. (2022). Airflow restriction mask induces greater central fatigue after a non-exhaustive high-intensity interval exercise. Scand. J. Med. Sci. Sports 32, 487–497. 10.1111/sms.14099 34787931

[B16] Dantas M. Barboza-Neto R. Guardieiro N. M. Pinto A. L. D. S. Gualano B. Saunders B. (2021). A cloth facemask increased ratings of perceived exertion and reduced affect, without affecting sprint or muscular performance. Res. Sports Med., 1–6. Online ahead of print. 10.1080/15438627.2021.2010202 34844490

[B17] Doherty C. J. Mann L. M. Angus S. A. Chan J. S. Molgat-Seon Y. Dominelli P. B. (2021). Impact of wearing a surgical and cloth mask during cycle exercise. Appl. Physiol. Nutr. Metab. 46, 753–762. 10.1139/apnm-2021-0190 33960846

[B18] Driver S. Reynolds M. Brown K. Vingren J. L. Hill D. W. Bennett M. (2021). Effects of wearing a cloth face mask on performance, physiological and perceptual responses during a graded treadmill running exercise test. Br. J. Sports Med. 56, 107–113. 10.1136/bjsports-2020-103758 33849908

[B19] Egger F. Blumenauer D. Fischer P. Venhorst A. Kulenthiran S. Bewarder Y. (2021). Effects of face masks on performance and cardiorespiratory response in well-trained athletes. Clin. Res. Cardiol. 111, 264–271. Online ahead of print. 10.1007/s00392-021-01877-0 34091726 PMC8179953

[B20] Ekkekakis P. Hall E. E. Petruzzello S. J. (2005). Variation and homogeneity in affective responses to physical activity of varying intensities: An alternative perspective on dose-response based on evolutionary considerations. J. Sports Sci. 23, 477–500. 10.1080/02640410400021492 16194996

[B21] Engeroff T. Groneberg D. A. Niederer D. (2021). The impact of ubiquitous face masks and filtering face piece application during rest, work and exercise on gas exchange, pulmonary function and physical performance: A systematic review with meta-analysis. Sports Med. Open 7, 92. 10.1186/s40798-021-00388-6 34897560 PMC8665851

[B22] Epstein D. Korytny A. Isenberg Y. Marcusohn E. Zukermann R. Bishop B. (2021). Return to training in the COVID-19 era: The physiological effects of face masks during exercise. Scand. J. Med. Sci. Sports 31, 70–75. 10.1111/sms.13832 32969531 PMC7646657

[B23] Feye A. S. P. Magallanes C. (2019). Efectos agudos y crónicos del uso de máscaras de entrenamiento en altura durante el ejercicio: Una revisión. Rev. Univ. De. La Educ. Física Y El Deporte 12, 53–65. 10.28997/ruefd.v0i12.6

[B24] Fikenzer S. Uhe T. Lavall D. Rudolph U. Falz R. Busse M. (2020). Effects of surgical and FFP2/N95 face masks on cardiopulmonary exercise capacity. Clin. Res. Cardiol. 109, 1522–1530. 10.1007/s00392-020-01704-y 32632523 PMC7338098

[B25] Fischer E. P. Fischer M. C. Grass D. Henrion I. Warren W. S. Westman E. (2020). Low-cost measurement of face mask efficacy for filtering expelled droplets during speech. Sci. Adv. 6, eabd3083. 10.1126/sciadv.abd3083 32917603 PMC7467698

[B26] Fukushi I. Nakamura M. Kuwana S. I. (2021). Effects of wearing facemasks on the sensation of exertional dyspnea and exercise capacity in healthy subjects. PLoS One 16, e0258104. 10.1371/journal.pone.0258104 34591935 PMC8483295

[B27] Haraf R. H. Faghy M. A. Carlin B. Josephson R. A. (2021). The physiological impact of masking is insignificant and should not preclude routine use during daily activities, exercise, and rehabilitation. J. Cardiopulm. Rehabil. Prev. 41, 1–5. 10.1097/HCR.0000000000000577 33351538 PMC7769052

[B28] Higgins J. P. Thomas J. Chandler J. Cumpston M. Li T. Page M. J. (2019). Cochrane handbook for systematic reviews of interventions. John Wiley & Sons. 10.1002/14651858.ED000142 PMC10284251 31643080

[B29] Higgins J. P. Thompson S. G. Deeks J. J. Altman D. G. (2003). Measuring inconsistency in meta-analyses. BMJ 327, 557–560. 10.1136/bmj.327.7414.557 12958120 PMC192859

[B30] Hopkins S. R. Dominelli P. B. Davis C. K. Guenette J. A. Luks A. M. Molgat-Seon Y. (2021). Face masks and the cardiorespiratory response to physical activity in health and disease. Ann. Am. Thorac. Soc. 18, 399–407. 10.1513/AnnalsATS.202008-990CME 33196294 PMC7919154

[B31] Jagim A. R. Dominy T. A. Camic C. L. Wright G. Doberstein S. Jones M. T. (2018). Acute effects of the elevation training mask on strength performance in recreational weight lifters. J. Strength Cond. Res. 32, 482–489. 10.1519/JSC.0000000000002308 29084093

[B32] Jesus J. P. Gomes M. Dias-Gonçalves A. Correia J. M. Pezarat-Correia P. Mendonca G. V. (2022). Effects of surgical masks on the responses to constant work-rate cycling performed at different intensity domains. Clin. Physiol. Funct. Imaging 42, 43–52. 10.1111/cpf.12734 34753208 PMC8646879

[B33] Kampert M. Singh T. Sahoo D. Han X. Van Iterson E. H. (2021). Effects of wearing an N95 respirator or cloth mask among adults at peak exercise: A randomized crossover trial. JAMA Netw. Open 4, e2115219. 10.1001/jamanetworkopen.2021.15219 34190998 PMC8246308

[B34] Kim J. H. Wu T. Powell J. B. Roberge R. J. (2016). Physiologic and fit factor profiles of N95 and P100 filtering facepiece respirators for use in hot, humid environments. Am. J. Infect. Control 44, 194–198. 10.1016/j.ajic.2015.08.027 26476496 PMC7115280

[B35] Lässing J. Falz R. Pökel C. Fikenzer S. Laufs U. Schulze A. (2020). Effects of surgical face masks on cardiopulmonary parameters during steady state exercise. Sci. Rep. 10, 22363. 10.1038/s41598-020-78643-1 33349641 PMC7752911

[B36] Liang M. Gao L. Cheng C. Zhou Q. Uy J. P. Heiner K. (2020). Efficacy of face mask in preventing respiratory virus transmission: A systematic review and meta-analysis. Travel Med. Infect. Dis. 36, 101751. 10.1016/j.tmaid.2020.101751 32473312 PMC7253999

[B37] López-Pérez M. E. Romero-Arenas S. Colomer-Poveda D. Keller M. Márquez G. (2020). Psychophysiological responses during a cycling test to exhaustion while wearing the elevation training mask. J. Strength Cond. Res. 36, 1282–1289. Online ahead of print. 10.1519/JSC.0000000000003626 846 32379243

[B38] Loturco I. Nakamura F. Y. Tricoli V. Kobal R. Cal Abad C. C. Kitamura K. (2015). Determining the optimum power load in jump squat using the mean propulsive velocity. PLoS One 10, e0140102. 10.1371/journal.pone.0140102 26444293 PMC4596801

[B39] Ludwig S. Zarbock A. (2020). Coronaviruses and SARS-CoV-2: A brief overview. Anesth. Analg. 131, 93–96. 10.1213/ANE.0000000000004845 32243297 PMC7173023

[B40] Mapelli M. Salvioni E. De Martino F. Mattavelli I. Gugliandolo P. Vignati C. (2021). You can leave your mask on": Effects on cardiopulmonary parameters of different airway protective masks at rest and during maximal exercise. Eur. Respir. J. 58, 2004473. 10.1183/13993003.04473-2020 33678608

[B41] Modena R. Fornasiero A. Callovini A. Savoldelli A. Pellegrini B. Schena F. (2021). Exercising at the time of the COVID-19 pandemic: Acute physiological, perceptual and performance responses of wearing face masks during sports activity. J. Sports Med. Phys. Fit. 62, 1329–1337. 10.23736/S0022-4707.21.12668-4 34913625

[B42] Morris N. B. Piil J. F. Christiansen L. Flouris A. D. Nybo L. (2020). Prolonged facemask use in the heat worsens dyspnea without compromising motor-cognitive performance. Temperature 8, 160–165. 10.1080/23328940.2020.1826840 PMC8098073 33997114

[B43] Motoyama Y. L. Joel G. B. Pereira P. E. A. Esteves G. J. Azevedo P. (2016). Airflow-restricting mask reduces acute performance in resistance exercise. Sports 4, 46. 10.3390/sports4040046 PMC5968897 29910294

[B44] Ng H. L. Trefz J. Schönfelder M. Wackerhage H. (2022). Effects of a taped filter mask on peak power, perceived breathlessness, heart rate, blood lactate and oxygen saturation during a graded exercise test in young healthy adults: A randomized controlled trial. BMC Sports Sci. Med. Rehabil. 14, 19. 10.1186/s13102-022-00410-8 35130956 PMC8819930

[B45] Öncen S. Pinar S. (2018). Effects of training mask on heart rate and anxiety during the graded exercise test and recovery. Eur. J. Phys. Educ. Sport Sci. 4. 10.5281/zenodo.1164390

[B46] Otsuka A. Komagata J. Sakamoto Y. (2022). Wearing a surgical mask does not affect the anaerobic threshold during pedaling exercise. jhse. 17 (1), 22–28. 10.14198/jhse.2022.171.03

[B47] Page M. J. Mckenzie J. E. Bossuyt P. M. Boutron I. Hoffmann T. C. Mulrow C. D. (2021). The PRISMA 2020 statement: An updated guideline for reporting systematic reviews. BMJ 372, n71. 10.1136/bmj.n71 33782057 PMC8005924

[B48] Person E. Lemercier C. Royer A. Reychler G. (2018). [Effect of a surgical mask on six minute walking distance]. Rev. Mal. Respir. 35, 264–268. 10.1016/j.rmr.2017.01.010 29395560

[B49] Ramirez-Moreno J. M. Ceberino D. Gonzalez Plata A. Rebollo B. Macias Sedas P. Hariramani R. (2020). Mask-associated 'de novo' headache in healthcare workers during the COVID-19 pandemic. Occup. Environ. Med. 78, 548–554. 10.1136/oemed-2020-106956 33380516

[B50] Reychler G. Straeten C. V. Schalkwijk A. Poncin W. (2021). Effects of surgical and cloth facemasks during a submaximal exercise test in healthy adults. Respir. Med. 186, 106530. 10.1016/j.rmed.2021.106530 34273733 PMC8452602

[B51] Roberge R. J. Benson S. Kim J. H. (2012a). Thermal burden of N95 filtering facepiece respirators. Ann. Occup. Hyg. 56, 808–814. 10.1093/annhyg/mes001 22294505

[B52] Roberge R. J. Coca A. Williams W. J. Powell J. B. Palmiero A. J. (2010). Physiological impact of the N95 filtering facepiece respirator on healthcare workers. Respir. Care 55, 569–577. 20420727

[B53] Roberge R. J. Kim J. H. Benson S. M. (2012b). Absence of consequential changes in physiological, thermal and subjective responses from wearing a surgical mask. Respir. Physiol. Neurobiol. 181, 29–35. 10.1016/j.resp.2012.01.010 22326638

[B54] Rojo-Tirado M. A. Benítez-Muñoz J. A. Alcocer-Ayuga M. Alfaro-Magallanes V. M. Romero-Parra N. Peinado A. B. (2021). Effect of different types of face masks on the ventilatory and cardiovascular response to maximal-intensity exercise. Biology 10, 969. 10.3390/biology10100969 34681068 PMC8533493

[B55] Romero-Arenas S. López-Pérez E. Colomer-Poveda D. Márquez G. (2021). Oxygenation responses while wearing the elevation training mask during an incremental cycling test. J. Strength Cond. Res. 35, 1897–1904. 10.1519/JSC.0000000000003038 30789572

[B56] Rosa B. V. Rossi F. E. Moura H. Santos A. Véras-Silva A. S. Ribeiro S. L. G. (2022). Effects of FFP2/N95 face mask on low- and high-load resistance exercise performance in recreational weight lifters. Eur. J. Sport Sci. 22 (9), 1326–1334. 10.1080/17461391.2021.1953613 34365900

[B57] Ryu J. S. Jong-Geun K. (2021). Effects of wearing face masks due to COVID-19 pandemic on cardiopulmonary exercise performance. J. Korean Phys. Educ. Assoc. 60, 355–365.

[B58] Sallis R. Young D. R. Tartof S. Y. Sallis J. F. Sall J. Li Q. (2021). Physical inactivity is associated with a higher risk for severe COVID-19 outcomes: A study in 48 440 adult patients. Br. J. Sports Med. 55, 1099–1105. 10.1136/bjsports-2021-104080 33849909

[B59] Shaw K. A. Zello G. A. Butcher S. J. Ko J. B. Bertrand L. Chilibeck P. D. (2021). The impact of face masks on performance and physiological outcomes during exercise: A systematic review and meta-analysis. Appl. Physiol. Nutr. Metab. 46, 693–703. 10.1139/apnm-2021-0143 33901405

[B60] Shaw K. Butcher S. Ko J. Zello G. A. Chilibeck P. D. (2020). Wearing of cloth or disposable surgical face masks has no effect on vigorous exercise performance in healthy individuals. Int. J. Environ. Res. Public Health 17, 8110. 10.3390/ijerph17218110 PMC7662944 33153145

[B61] Shurlock J. Muniz-Pardos B. Tucker R. Bachl N. Papadopoulou T. Holloway G. (2021). Recommendations for face coverings while exercising during the COVID-19 pandemic. Sports Med. Open 7, 19. 10.1186/s40798-021-00309-7 33721127 PMC7957452

[B62] Slimani M. Miarka B. Znazen H. Moalla W. Hammami A. Paravlic A. (2021). Effect of a warm-up protocol with and without facemask-use against COVID-19 on cognitive function: A pilot, randomized counterbalanced, cross-sectional study. Int. J. Environ. Res. Public Health 18, 5885. 10.3390/ijerph18115885 34070866 PMC8197822

[B63] Steinhilber B. Seibt R. Gabriel J. Brountsou J. Muljono M. Downar T. (2022). Effects of face masks on physical performance and physiological response during a submaximal bicycle ergometer test. Int. J. Environ. Res. Public Health 19, 1063. 10.3390/ijerph19031063 35162087 PMC8834111

[B64] Sterne J. A. C. Savović J. Page M. J. Elbers R. G. Blencowe N. S. Boutron I. (2019). RoB 2: A revised tool for assessing risk of bias in randomised trials. BMJ 366, l4898. 10.1136/bmj.l4898 31462531

[B65] Tornero-Aguilera J. F. Rubio-Zarapuz A. Bustamante-Sánchez A. Clemente-Suárez V. J. (2021). The effect of surgical mask use in anaerobic running performance. Appl. Sci. 11, 6555. 10.3390/app11146555

[B66] Umutlu G. Acar N. E. Sinar D. S. Akarsu G. Güven E. Yildirim İ. (2021). COVID-19 and physical activity in sedentary individuals: Differences in metabolic, cardiovascular, and respiratory responses during aerobic exercise performed with and without a surgical face masks. J. Sports Med. Phys. Fit. 62, 851–858. 10.23736/S0022-4707.21.12313-8 33885256

[B67] Wong A. Y. Ling S. K. Louie L. H. Law G. Y. So R. C. Lee D. C. (2020). Impact of the COVID-19 pandemic on sports and exercise. Asia. Pac. J. Sports Med. Arthrosc. Rehabil. Technol. 22, 39–44. 10.1016/j.asmart.2020.07.006 32821648 PMC7386263

[B68] Yoshihara A. Dierickx E. E. Brewer G. J. Sekiguchi Y. Stearns R. L. Casa D. J. (2021). Effects of face mask use on objective and subjective measures of thermoregulation during exercise in the heat. Sports Health 13, 463–470. 10.1177/19417381211028212 34196240 PMC8404762

[B69] Zhang G. Li M. Zheng M. Cai X. Yang J. Zhang S. (2021). Effect of surgical masks on cardiopulmonary function in healthy young subjects: A crossover study. Front. Physiol. 12, 710573. 10.3389/fphys.2021.710573 34566679 PMC8461071

[B70] Zimmerman N. J. Eberts C. Salvendy G. McCabe G. (1991). Effects of respirators on performance of physical, psychomotor and cognitive tasks. Ergonomics 34, 321–334. 10.1080/00140139108967316 2055218

