# Supplementary figures and images for: Facial mask acute effects on affective/psychological and exercise performance responses during exercise: A meta-analytical review
Source: Front Physiol. 2022 Nov 2;13:994454. doi: 10.3389/fphys.2022.994454 (PMC9667098; doi:10.3389/fphys.2022.994454)

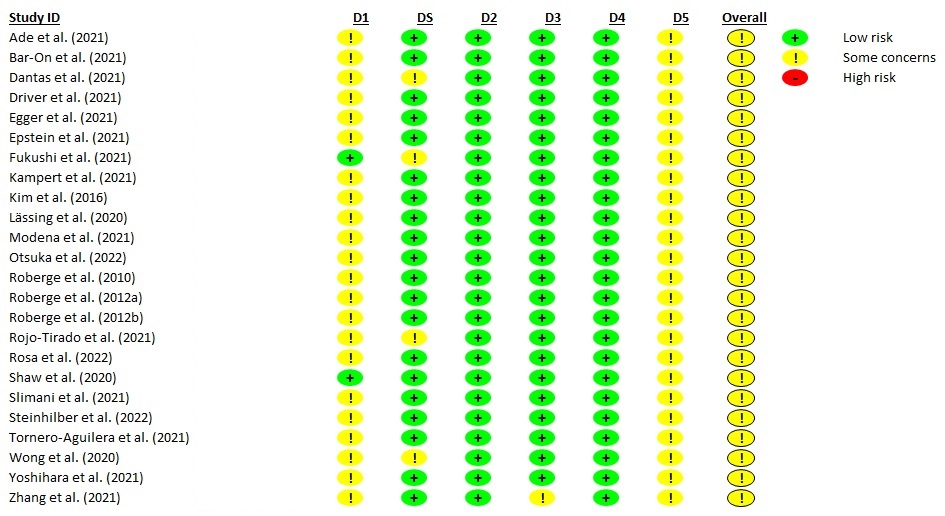

Supplement: Supplementary file 1 [file Image3.JPEG]

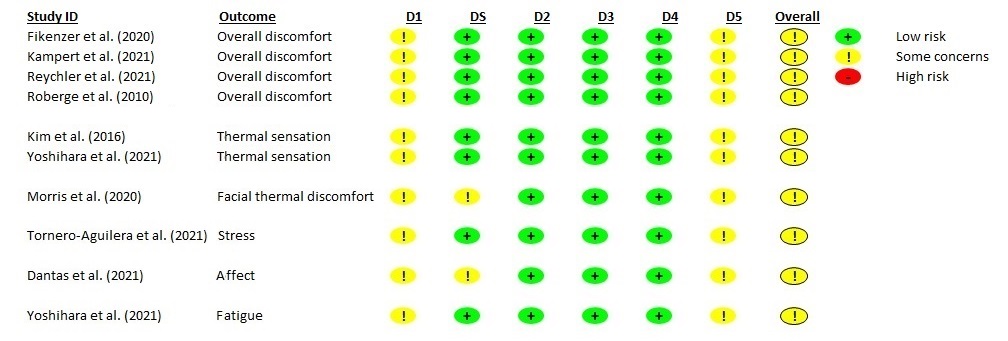

Supplement: Supplementary file 2 [file Image1.JPEG]

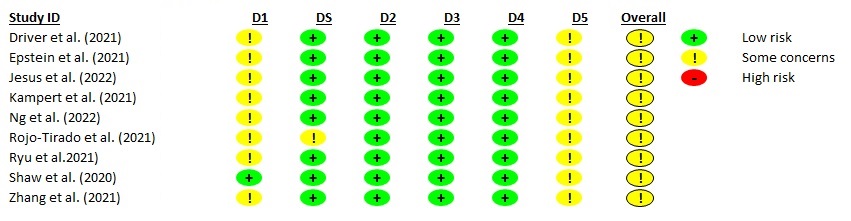

Supplement: Supplementary file 3 [file Image4.JPEG]

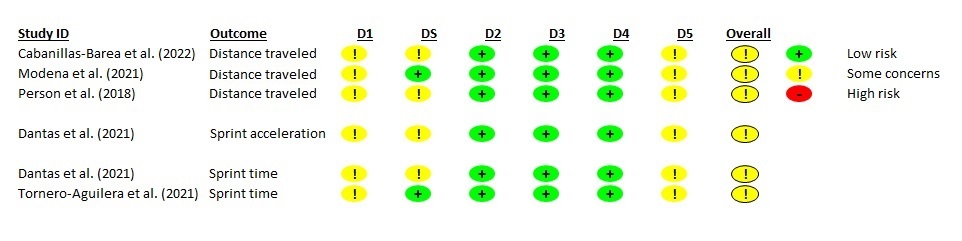

Supplement: Supplementary file 4 [file Image7.JPEG]

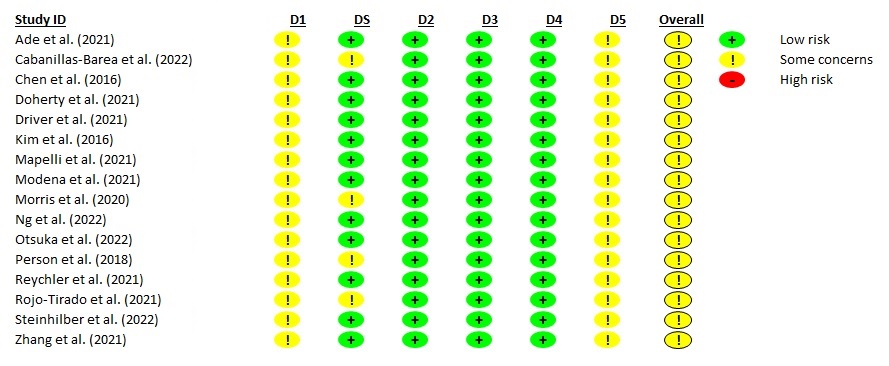

Supplement: Supplementary file 5 [file Image2.JPEG]

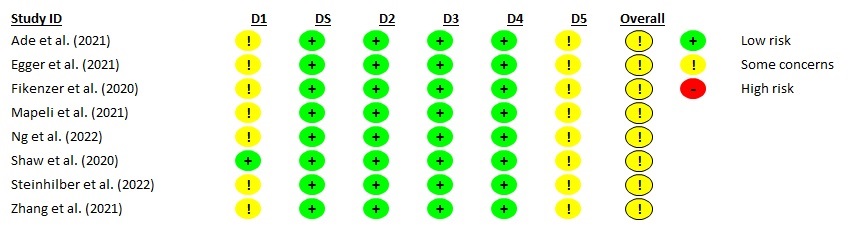

Supplement: Supplementary file 6 [file Image5.JPEG]

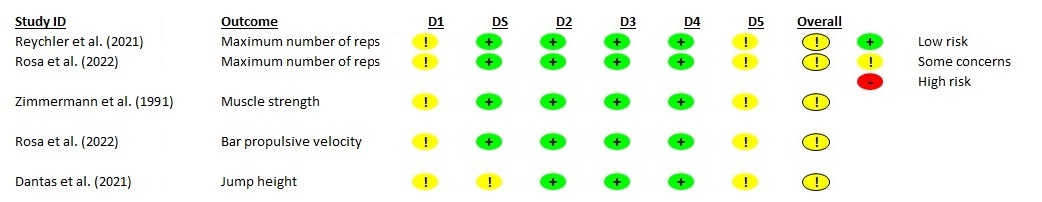

Supplement: Supplementary file 9 [file Image6.JPEG]
