# Supplementary material for: Facial mask acute effects on affective/psychological and exercise performance responses during exercise: A meta-analytical review
Source: Front Physiol. 2022 Nov 2;13:994454. doi: 10.3389/fphys.2022.994454 (PMC9667098; doi:10.3389/fphys.2022.994454)
Supplement: Supplementary file 8 [file DataSheet1.docx]

Electronic Supplementary Material Appendix

# **Table S1.** PRISMA checklist.

| **Section/topic** | **#** | **Checklist item** | **Reported on page #** |
| --- | --- | --- | --- |
| **TITLE** | | |  |
| Title | 1 | Identify the report as a systematic review, meta-analysis, or both. | 1 |
| **ABSTRACT** | | |  |
| Structured summary | 2 | Provide a structured summary including, as applicable: background; objectives; data sources; study eligibility criteria, participants, and interventions; study appraisal and synthesis methods; results; limitations; conclusions and implications of key findings; systematic review registration number. | 2 |
| **INTRODUCTION** | | |  |
| Rationale | 3 | Describe the rationale for the review in the context of what is already known. | 3-4 |
| Objectives | 4 | Provide an explicit statement of questions being addressed with reference to participants, interventions, comparisons, outcomes, and study design (PICOS). | 4 |
| **METHODS** | | |  |
| Protocol and registration | 5 | Indicate if a review protocol exists, if and where it can be accessed (e.g., Web address), and, if available, provide registration information including registration number. | 4 |
| Eligibility criteria | 6 | Specify study characteristics (e.g., PICOS, length of follow-up) and report characteristics (e.g., years considered, language, publication status) used as criteria for eligibility, giving rationale. | 4-5 |
| Information sources | 7 | Describe all information sources (e.g., databases with dates of coverage, contact with study authors to identify additional studies) in the search and date last searched. | 4-5 |
| Search | 8 | Present full electronic search strategy for at least one database, including any limits used, such that it could be repeated. | 4 and Table S2 |
| Study selection | 9 | State the process for selecting studies (i.e., screening, eligibility, included in systematic review, and, if applicable, included in the meta-analysis). | 4-5 |
| Data collection process | 10 | Describe method of data extraction from reports (e.g., piloted forms, independently, in duplicate) and any processes for obtaining and confirming data from investigators. | 5-6 |
| Data items | 11 | List and define all variables for which data were sought (e.g., PICOS, funding sources) and any assumptions and simplifications made. | 4-5 |
| Risk of bias in individual studies | 12 | Describe methods used for assessing risk of bias of individual studies (including specification of whether this was done at the study or outcome level), and how this information is to be used in any data synthesis. | 6 |
| Summary measures | 13 | State the principal summary measures (e.g., risk ratio, difference in means). | 5-6 |
| Synthesis of results | 14 | Describe the methods of handling data and combining results of studies, if done, including measures of consistency (e.g., I^2^) for each meta-analysis. | 5-6 |

| **Section/topic** | **#** | **Checklist item** | **Reported on page #** |
| --- | --- | --- | --- |
| Risk of bias across studies | 15 | Specify any assessment of risk of bias that may affect the cumulative evidence (e.g., publication bias, selective reporting within studies). | 6 |
| Additional analyses | 16 | Describe methods of additional analyses (e.g., sensitivity or subgroup analyses, meta-regression), if done, indicating which were pre-specified. | 5-6 |
| **RESULTS** | | |  |
| Study selection | 17 | Give numbers of studies screened, assessed for eligibility, and included in the review, with reasons for exclusions at each stage, ideally with a flow diagram. | 6 and Figure 1 |
| Study characteristics | 18 | For each study, present characteristics for which data were extracted (e.g., study size, PICOS, follow-up period) and provide the citations. | 6-8 |
| Risk of bias within studies | 19 | Present data on risk of bias of each study and, if available, any outcome level assessment (see item 12). | 12 and Figures S1-S7 |
| Results of individual studies | 20 | For all outcomes considered (benefits or harms), present, for each study: (a) simple summary data for each intervention group (b) effect estimates and confidence intervals, ideally with a forest plot. | Table 1 and Figures 2-7 |
| Synthesis of results | 21 | Present results of each meta-analysis done, including confidence intervals and measures of consistency. | 8-12 and Figures 2-7 |
| Risk of bias across studies | 22 | Present results of any assessment of risk of bias across studies (see Item 15). | 12-13 |
| Additional analysis | 23 | Give results of additional analyses, if done (e.g., sensitivity or subgroup analyses, meta-regression [see Item 16]). | 9-10 |
| **DISCUSSION** | | |  |
| Summary of evidence | 24 | Summarize the main findings including the strength of evidence for each main outcome; consider their relevance to key groups (e.g., healthcare providers, users, and policy makers). | 13 and 17 |
| Limitations | 25 | Discuss limitations at study and outcome level (e.g., risk of bias), and at review-level (e.g., incomplete retrieval of identified research, reporting bias). | 16-17 |
| Conclusions | 26 | Provide a general interpretation of the results in the context of other evidence, and implications for future research. | 17 |
| **FUNDING** | | |  |
| Funding | 27 | Describe sources of funding for the systematic review and other support (e.g., supply of data); role of funders for the systematic review. | 1 |

*From:*  Moher D, Liberati A, Tetzlaff J, Altman DG, The PRISMA Group (2009). Preferred Reporting Items for Systematic Reviews and Meta-Analyses: The PRISMA Statement. PLoS Med 6(7): e1000097. doi:10.1371/journal.pmed1000097

For more information, visit: **www.prisma-statement.org**.

# **Table S2.** Full search strategy.

| **ITEMS** | **SEARCH STRATEGY IN DATABASES** |
| --- | --- |
|  |  |
| **Population related** | “Adult” OR “Athlete” |
|  |  |
| **Face mask related** | AND “Mask*” OR “Respiratory Protective Devices” OR “Respiratory Protection” |
|  |  |
| **Exercise related** | AND “Exercise” OR “Sport*” OR “Physical Activity” OR “Physical Activit*” OR “Physical activity, Capacity and Performance” OR “Athletic Performance” OR “Sport” OR “Aerobic Exercise” OR “Resistance Training” |

**Table S3 Criteria of Newcastle–Ottawa Quality Assessment for cohort studies**

| **Selection** (Exercise intervention was in pregnancy) |
| --- |
| 1) Representativeness of the exposed cohort |
| a) Truly representative of common maternal population *(1 star)* |
| b) Somewhat representative *(1 star)* |
| c) Selected group |
| d) No description of derivation of cohort |
| 2) Selection of non-exposed cohort |
| a) Drawn from same community as exposed cohort *(1 star)* |
| b) Drawn from a different source |
| c) No description of the derivation of the non-exposed cohort |
| 3) Ascertainment of exposure |
| a) Secure record (e.g., exercise intervention during pregnancy) *(1 star)* |
| b) Structured interview *(1 star)* |
| c) Written self-report |
| d) No description |
| e) Other |
| 4) Demonstration that outcome of interest was not present at start of study |
| a) Yes *(1 star)* |
| b) No |
| **Comparability** |
| 1) Comparability of cohorts on the basis of the design or analysis controlled for confounders |
| a) The study controls for age, weight, parity, body mass index and healthy status *(1 star)* |
| b) Study controls for other factors *(1 star)* |
| c) Cohorts are not comparable on basis of design or analysis controlled for confounders |
| **Outcome** |
| 1) Assessment of outcome |
| a) Independent blind assessment *(1 star)* |
| b) Record linkage *(1 star)* |
| c) Self-report |
| d) No description |
| e) Other |
| 2) Was follow-up long enough for outcomes to occur |
| a) Yes *(1 star)* |
| b) No |
| 3) Adequacy of follow up of cohorts |
| a) Complete follow up - all subjects accounted *(1 star)* |
| b) Subjects lost to follow up unlikely to introduce bias - number lost less than or equal to 20% or description of those lost suggested no different from those followed *(1 star)* |
| c) Follow up rate less than 80% and no description of those lost |
| d) No statement |

**Table S4 Excluded full texts references in meta-analysis from eligible criteria screening**

| **Reason for exclusion** | **Reference** |
| --- | --- |
| **Review (18)** | (1)(2)(3)(4)(5)(6)(7)(8)(9)(10)(11)(12)(13)(14)(15)(16)(17)(18) |
| **Short of early life and child data (30)** | (19)(20)(21)(22)(23)(24)(25)(26)(27)(28)(29)(30)(31)(32)(33)(34)(35)(36)(37)(38)  (39)(40)(41)(42)(43)(44)(45)(46)(47)(48) |
| **Short of pregnancy exercise treatment (17)** | (49)(50)(51)(52)(53)(54)(55)(56)(57)(58)(59)(60)(61)(62)(63)(64)(65) |
| **No separation outcomes between treatments (34)** | (66)(67)(68)(69)(70)(71)(72)(73)(74)(75)(76)(77)(78)(79)(80)(81)(82)(83)(84) (84)(86)(87)(88)(89)(90)(91)(92)(93)(94)(95)(96)(97)(98)(99) |
| **Others (9)** | (100)(101)(102)(103)(104)(105)(106)(107)(108) |
|  |  |
| **Excluded references** | |
| 1. Gavard JA, Artal R. Effect of Exercise on Pregnancy Outcome. Clin Obstet Gynecol. 2008;51(2):467–80.  2. Penney DS. The Effect of Vigorous Exercise During Pregnancy. J Midwifery Womens Heal. 2008;53:155–9.  3. Melzer K, Schutz Y, Boulvain M, Kayser B. Physical Activity and Pregnancy. Sport Med. 2010;40(6):493–507.  4. Ferraro ZM, Gaudet L, Adamo KB. The potential impact of physical activity during pregnancy on maternal and neonatal outcomes. Obstet Gynecol Surv. 2012;67(2):99–110.  5. Sui Z, Dodd JM. Exercise in obese pregnant women: positive impacts and current perceptions. Int J Womens Health. 2013;5(July):389–98.  6. Muktabhant B, Ta L, Lumbiganon P, Laopaiboon M. Diet or exercise, or both, for preventing excessive weight gain in pregnancy. Cochrane Database Syst Rev. 2015;6(6):1–210.  7. Poyatos-león R, Sanabria-martínez G, García-prieto JC, Álvarez-bueno C, Pozuelo-carrascosa DP, Cavero-redondo I, et al. A follow-up study to assess the determinants and consequences of physical activity in pregnant women of Cuenca, Spain. BMC Public Health [Internet]. 2016;16:437–43. Available from: http://dx.doi.org/10.1186/s12889-016-3130-x  8. Garcı A, Poyatos-leo R. Effects of Exercise-Based Interventions on Neonatal Outcomes: A Meta-Analysis of Randomized Controlled Trials. Am J Heal Promot. 2016;30(4):214–23.  9. Barakat R, Perales M. Resistance Exercise in Pregnancy and Outcome. Clin Obstet Gynecol. 2016;59(3):591–9.  10. Rogozin E, Marlin N, Jackson L, Rayanagoudar G, Ruifrok AE, Dodds J, et al. Effects of antenatal diet and physical activity on maternal and fetal outcomes: individual patient data meta-analysis and health economic evaluation. Health Technol Assess (Rockv). 2017;21(41):1–194.  11. Perales M. Exercise During Pregnancy. Am Med Assoc. 2017;317(11):1113–4.  12. Dodd JM, Grivell RM, Louise J, Deussen AR, Giles L, Mol BW, et al. The effects of dietary and lifestyle interventions among pregnant women who are overweight or obese on longer-term maternal and early childhood outcomes: protocol for an individual participant data (IPD) meta-analysis. Syst Rev. 2017;6(51):1–12.  13. Mcdonald SM, Yeo S, Liu J, Wilcox S, Sui X, Pate RR. Associations between maternal physical activity and fitness during pregnancy and infant birthweight. Prev Med Reports. 2018;11(April):1–6.  14. Siega-Riz AM. Modification of lifestyle behavior during pregnancy for prevention of childhood obesity. www.thelancet.com/child-adolescent. 2018;2(9):771–2.  15. Dodd JM, Deussen AR, Louise J. Optimising gestational weight gain and improving maternal and infant health outcomes through antenatal dietary, lifestyle and physical activity advice: the OPTIMISE randomised controlled trial protocol. BMJ Open. 2018;8(2):1–5.  16. Matsuzaki M, Kusaka M, Sugimoto T, Shiraishi M, Kobayashi R, Watanabe S, et al. The effects of a Yoga exercise and nutritional guidance program on pregnancy outcomes among healthy pregnant Japanese Women: A study protocol for a randomized controlled trial. J Altern Complement Med. 2018;24(6):603–10.  17. Askie LM, Martin A, Daniels LA, Hesketh KD, Rissel C, Taylor B, et al. Interventions commenced by early infancy to prevent childhood obesity — The EPOCH Collaboration: An individual participant data prospective meta-analysis of four randomized controlled trials. Pediatr Obes. 2020;15(6):1–10.  18. Dixon L. Effects of physical exercise on pregnancy outcomes: a meta-analytic review. Med Sci Sport Exerc. 1991;23(11):1234–9.  19. Kulpa PJ, White BM, Visscher R. Aerobic exercise in pregnancy. Am J Obstet Gynecol. 1987;156(6):1395–403.  20. Alderman BW, Zhao HUI, Holt VL, Watts DH, Beresford SAA. Maternal Physical Activity in Pregnancy and Infant Size for Gestational Age. AEP. 1998;8(8):513–9.  21. Cavalli AS, Tanaka T. Maternal Leisure-time Physical Activities are not Determinant Risk Factors of Low Birthweight Babies : A Cross-sectional Study of 1 , 714 Pregnant Women. Environ Health Prev Med. 2000;5(7):72–80.  22. Niuewenhuijsen MJ, Northstone K, Golding J, Team AS. Swimming and birth weight. Epidemiology. 2002;13(4):Supplement.  23. Bick-sander A, Steiner B, Wolf SA, Babu H, Kempermann G. Running in pregnancy transiently increases postnatal hippocampal neurogenesis in the offspring. PNAS. 2006;103(10):3852–7.  24. Owe KM, Nystad W, Bø K. Correlates of regular exercise during pregnancy: the Norwegian Mother and Child Cohort Study. Scand J Med Sci Sport. 2009;19(5):637–45.  25. Mbada CE, Adebayo OE, Adeyemi AB, Arije OO, Dada OO, Akinwande OA, et al. Knowledge and Attitude of Nigerian Pregnant Women towards Antenatal Exercise: A Cross-Sectional Survey. ISRN Obstet Gynecol. 2014;2014:1–8.  26. Wijden CL Van Der, Waal HAD De, Mechelen W Van, Mireille NM. The relationship between moderate-to-vigorous intensity physical activity and insulin resistance, insulin-like growth factor (IGF-1)-system 1, leptin and weight change in healthy women during pregnancy and after delivery. Clin Endocrinol (Oxf). 2015;82(August):68–75.  27. Harris ST, Liu J, Wilcox S, Moran R, Gallagher A. Exercise During Pregnancy and its Association with Gestational Weight Gain. Matern Child Heal J. 2015;19:528–37.  28. Mohd-shukri NA, Duncan A, Denison FC, Forbes S, Walker BR, Norman JE, et al. Health Behaviours during Pregnancy in Women with Very Severe Obesity. Nutrients. 2015;7:8431–43.  29. Ehrlich SF, Sternfeld B, Krefman AE, Charles LC, Jr PQ, Ferrara A. Moderate and Vigorous Intensity Exercise During Pregnancy and Gestational Weight Gain in Women with Gestational Diabetes. Matern Child Health J. 2016;20(6):1254–64.  30. Egeland GM, Tell GS, Næss Ø, Igland J, Klungsøyr K. Association between pregravid physical activity and family history of stroke and risk of stillbirth: population-based cohort study. BMJ Open. 2017;7:1–8.  31. Qun Y, Liu Y, Hua Y, Li X. Effect of diet and exercise intervention in Chinese pregnant women on gestational weight gain and perinatal outcomes: A quasi-experimental study. Appl Nurs Res. 2017;36:50–6.  32. Moran LJ, Flynn AC, Louise J, Deussen AR. The Effect of a Lifestyle Intervention on Pregnancy and Postpartum Dietary Patterns Determined by Factor Analysis. Diabetes. 2017;25(6):1022–32.  33. Watelain E, Pinti A, Doya R, Garnier C, Toumi H. Benefits of physical activities centered on the trunk for pregnant women. Phys Sportsmed. 2017;45(3):293–302.  34. Pt GH, Eskild A, Owe KM, Pt KB, Bjelland EK. Exercise in pregnancy: an association with placental weight? Am J Obstet. 2017;216(February):1–9.  35. Lindsay AC, Wallington SF, Greaney ML, Machado MMT, Andrade GP De. Patient– Provider Communication and Counseling about Gestational Weight Gain and Physical Activity: A Qualitative Study of the Perceptions and Experiences of Latinas Pregnant with their First Child. Int J Environ Res Public Health. 2017;14:1–13.  36. Kuhrt K, Harmon M, Hezelgrave NL, Seed PT, Shennan AH. Is recreational running associated with earlier delivery and lower birth weight in women who continue to run during pregnancy? An international retrospective cohort study of running habits of 1293 female runners during pregnancy. BMJ Open Sport Exerc Med. 2018;4(1):1–6.  37. Badon SE, Miller RS, Qiu C, Sorensen TK, Williams MA, Enquobahrie DA, et al. Maternal healthy lifestyle during early pregnancy and offspring birthweight: differences by offspring sex. J Matern Neonatal Med. 2018;31(9):1111–7.  38. Vasapollo B, Presti DLO, Gagliardi G, Farsetti D, Tiralongo GM. Restricted physical activity in pregnancy reduces maternal vascular resistance and improves fetal growth. Ultrasound Obs Gynecol. 2018;51(5):672–6.  39. Acosta-manzano P, Coll-risco I, Poppel MNM Van, Femia P, Romero-gallardo L, Borges-cosic M, et al. Influence of a Concurrent Exercise Training Intervention during Pregnancy on Maternal and Arterial and Venous Cord Serum Cytokines: The GESTAFIT Project. J Clin Med. 2019;8(11):1–20.  40. Babili MG, Amerikanou C, Papada E, Christopoulos G, Tzavara C, Kaliora AC. The effect of prenatal maternal physical activity and lifestyle in perinatal outcome: results from a Greek study. Eur J Public Heal. 2019;30(2):328–32.  41. Zhen-duan J, Engebretsen B, Laroche HH, Zhen-duan J. Diet and physical activity changes among low- income families: perspectives of mothers and their children of mothers and their children. Int J Qual Stud Heal Well-being. 2019;14(1):1–14.  42. Daley A, Jolly K, Jebb SA, Roalfe A, Mackilllop L, Lewis A, et al. Effectiveness of a behavioural intervention involving regular weighing and feedback by community midwives within routine antenatal care to prevent excessive gestational weight gain: POPS2 randomised controlled trial. BMJ Open. 2019;9(9):1–10.  43. Haakstad LAH, Kissel I, Bø K. Long-term effects of participation in a prenatal exercise intervention on body weight, body mass index, and physical level: a 6-year follow up study of a randomized controlled trial. J Matern Fetal Neonatal Med. 2019;7(July):1–9.  44. Leppänen MH, Raitanen J, Husu P, Kujala UM, Tuominen PPA, Vähä-ypyä H, et al. Physical Activity and Body Composition in Children and Their Mothers According to Mother’ s Gestational Diabetes Risk: A Seven-Year Follow-Up Study. Med. 2019;55(10).  45. Lott ML, Power ML. Patient Attitudes toward Gestational Weight Gain and Exercise during Pregnancy. J Pregnancy. 2019;2019:1–8.  46. Livres T, Themes F, Werneck AO, Silva DR, Collings PJ. Prenatal, biological and environmental factors associated with physical activity maintenance from childhood to adolescence. Cien Saude Colet. 2019;24(3):1201–10.  47. Charkamyani F, Hosseinkhani A, Samani LN. Reducing the Adverse Maternal and Fetal Outcomes in IVF Women by Exercise Interventions During Pregnancy. Res Q Exerc Sport. 2019;90(4):589–99.  48. Xiang M, Konishi M, Hu H, Nishimaki M, Kim H. When and How Should Chinese Pregnant Women Exercise? A Longitudinal Study in China. Int J Env Res Public Heal. 2020;17(1):1–16.  49. Fulkerson JA, French SA, Story M, Hannan PJ, Neumark-sztainer D, Himes JH, et al. Weight-Bearing Physical Activity among Girls and Mothers: Relationships to Girls’ Weight Status. Obes Res. 2004;12(2):258–66.  50. Hallal PC, Wells JCK, Reichert FF, Anselmi L, Victora CG. Early determinants of physical activity in adolescence: prospective birth cohort study. BMJ. 2006;29(April):1002–7.  51. Neggers Y, Goldenberg R, Cliver S, Hauth J. The relationship between psychosocial profile, health practices, and pregnancy outcomes. Acta Obstet Gynecol. 2006;85(March 2005):277–85.  52. Chasan-taber L, Evenson KR, Sternfeld B, Chasan-taber L. Assessment of Recreational Physical Activity During Pregnancy in Epidemiologic Studies of Birthweight and Length of Gestation: Methodologic Aspects. Women&Health. 2008;45(4):85–107.  53. Moschonis G, Grammatikaki E, Manios Y. Perinatal predictors of overweight at infancy and preschool childhood: the GENESIS study. Int J Obes. 2008;32(December):39–47.  54. Voldner N, Frøslie KF, Anette L, Haakstad H, Bø K, Henriksen T. Birth complications, overweight, and physical inactivity. Acta Obstet Gynecol. 2009;88(February):550–5.  55. Brien MO, Nader PR, Houts RM, Bradley R, Friedman SL, Belsky J, et al. The ecology of childhood overweight: a 12-year longitudinal analysis. Int J Obes. 2007;31(November):1469–78.  56. Abeysena C, Jayawardana P. Effect of psychosocial stress and physical activity on preterm birth: A cohort study. J Obstet Gynaecol Res. 2010;36(2):260–7.  57. Oliver M, Schluter PJ, Paterson J, Kolt GS, Schofield GM. Pacific islands families: Child and parental physical activity and body size - design and methodology. J New Zeal Med Assoc. 2009;122(July):48–59.  58. Phelan S, Hart C, Phipps M, Abrams B, Schaffner A, Adams A, et al. Maternal Behaviors during Pregnancy Impact Offspring Obesity Risk. Exp Diabetes Res. 2011;2011(August):1–9.  59. Fan C, Huang T, Cui F, Gao M, Song L, Wang S. Paternal factors to the offspring birth weight: the 829 birth cohort study. Int J Clin Exp Med. 2015;8(7):11370–8.  60. Cederberg H, Jokelainen J, Mikkola I, Rajala U, Kein S. Association of maternal smoking during pregnancy with aerobic fitness of offspring in young adulthood: a prospective cohort study. An Int J Obstet Gynaecol. 2015;123:1789–95.  61. Tikanmäki M, Tammelin T, Vääräsmäki M, Sipola-leppänen M, Miettola S. Prenatal determinants of physical activity and cardiorespiratory fitness in adolescence – Northern Finland Birth Cohort 1986 study. BMC Public Health. 2017;17:1–12.  62. Friis J, Bohman B, Berglind D, Hansson LM, Frederiksen P, Lykke E, et al. Cross-sectional associations between maternal self-efficacy and dietary intake and physical activity in four-year-old children of first-time Swedish mothers. Appetite [Internet]. 2018;125:131–8. Available from: https://doi.org/10.1016/j.appet.2018.01.026  63. Taylor RW, Gray AR, Heath AM, Galland BC, Lawrence J, Sayers R, et al. Sleep, nutrition, and physical activity interventions to prevent obesity in infancy: follow-up of the Prevention of Overweight in Infancy (POI) randomized controlled trial at ages 3.5 and 5y. Am J Clin Nutr. 2018;108(2):228–36.  64. Murray-davis B, Grenier L, Atkinson SA, Mottola MF, Wahoush O, Thabane L, et al. Experiences regarding nutrition and exercise among women during early postpartum: a qualitative grounded theory study. BMC Pregnancy Childbirth. 2019;19:1–11.  65. Perreault M, Moore C, Fusch G, Teo K, Atkinson S. Factors Associated with Serum 25-Hydroxyvitamin D Concentration in Two Cohorts of Pregnant Women in Southern Ontario, Canada. Nutrients. 2019;11(1):pii:E123.  66. Rice PL, Fort IL. The relationship of maternal exercise on labor, delivery and health of the newborn. J Sports Med Phys Fitness. 1991;31(1):95–9.  67. Schramm WF, Stockbauer JW, Hoffman HJ. Exercise, Employment, other daily activities, and adverse pregnancy outcomes. Am J Epidemiol. 1996;143(3):211–8.  68. Campbell MK, Mottola MF. Recreational exercise and occupational activity during pregnancy and birth weight : A case-control study. AM J Obs Gynecol. 2001;184(June):403–8.  69. Davison KK, Birch LL. Child and parent characteristics as predictors of change in girls’ body mass index. Int J Obes. 2001;25(June):1834–42.  70. Runyan SM, Stadler DD, Bainbridge CN, Miller SC, Moyer-mileur LJ. Familial resemblance of bone mineralization, calcium intake, and physical activity in early-adolescent daughters, their mothers, and maternal grandmothers. J Am Diet Assoc. 2003;103(10):1320–5.  71. Watson PE, Mcdonald BW. Activity levels in pregnant New Zealand women: relationship with socioeconomic factors, well- being, anthropometric measures, and birth outcome. Appl Physiol Nutr Metab. 2007;32(July):733–42.  72. Juhl M, Olsen J, Andersen PK, Nøhr EA, Andersen AN. Physical exercise during pregnancy and fetal growth measures: a study within the Danish National Birth Cohort. AM J Obs Gynecol [Internet]. 2010;202(1):63.e1-63.e8. Available from: http://dx.doi.org/10.1016/j.ajog.2009.07.033  73. Bae HS. Lifestyle, nutrient intake, iron status, and pregnancy outcome in pregnant women of advanced maternal age. Nutr Res Pract. 2011;5(1):52–9.  74. Dumith SC, Domingues MR, Mendoza-Sassi RA, Cesar JA. Physical activity during pregnancy and its association with maternal and child health. Rev Saude Publica. 2012;46(2):1–6.  75. Mattran K, Mudd LM, Rudey RA, Kelly JSC. Leisure-Time Physical Activity During Pregnancy and Offspring Size at 18 to 24 Months. J Phys Act Heal. 2011;8(5):655–62.  76. Ortega FB, Ruiz JR, Hurtig-wennlöf A, Meirhaeghe A, González-gross M, Moreno LA, et al. Physical Activity Attenuates the Effect of Low Birth Weight on Insulin Resistance in Adolescents. Diabetes. 2011;60(September):2295–9.  77. Abeysena C, Jayawardana P. Sleep deprivation, physical activity and low income are risk factors for inadequate weight gain during pregnancy: A cohort study. J Obstet Gynaecol Res. 2011;37(7):734–40.  78. Mustila T, Raitanen J, Keskinen P, Saari A, Luoto R. Lifestyle counseling during pregnancy and offspring weight development until four years of age: follow-up study of a controlled trial. J Negat Results Biomed. 2012;11(11):1–8.  79. Bisson M, Alméras N, Plaisance J, Rhéaume C, Bujold E, Tremblay A, et al. Maternal fitness at the onset of the second trimester of pregnancy: correlates and relationship with infant birth weight. Pediatr Obes. 2012;8(6):464–74.  80. Montpetit AE, Plourde H, Cohen TR, Koski KG. Modeling the impact of prepregnancy BMI, physical activity and energy intake on gestational weight gain, infant birth weight, and postpartum weight retention. J Phys Act Health. 2012;9(7):1020–9.  81. Korpelainen R. Physical activity and fitness in 8-year-old overweight and normal weight children and their parents. Int J Circumpolar Health. 2012;71:1–10.  82. Olvera N, Leung P, Kellam SF, Smith DW, Liu J. Summer and Follow-Up Interventions to Affect Adiposity with Mothers and Daughters. Am J Prev Med [Internet]. 2013;44(3):S258–66. Available from: http://dx.doi.org/10.1016/j.amepre.2012.11.018  83. Flores-peña Y, Ortiz-félix RE, Ávila-alpirez H, Alba-alba CM, Hernández-carranco RG. Maternal Eating and Physical Activity Strategies and their Relation with Children’ s Nutritional Status. Rev Lat Am Enferm. 2014;22(2):286–92.  84. Drenowatz C, Erkelenz N, Wartha O, Brandstetter S. Parental Characteristics Have a Larger Effect on Children’ s Health Behaviour than Their Body Weight. Eur J Obes. 2014;7:388–98.  85. Mourtakos SP, Tambalis KD, Panagiotakos DB, Antonogeorgos G, Arnaoutis G, Karteroliotis K, et al. Maternal lifestyle characteristics during pregnancy, and the risk of obesity in the offspring: a study of 5125 children. BMC Pregnancy Childbirth. 2015;16(66):4–11.  86. Esteban-cornejo I, Martinez-gomez D, Tejero-gonzález CM, Izquierdo-gomez R, Carbonell-baeza A, Castro-piñero J, et al. Maternal physical activity before and during the prenatal period and the offspring’s academic performance in youth. The UP&DOWN study. J Matern Neonatal Med. 2016;29(9):1414–20.  87. Connor SGO, Koprowski C, Dzubur E, Leventhal AM, Huh J, Dunton GF. Differences in Mothers’ and Children’s Dietary Intake during Physical and Sedentary Activities: An Ecological Momentary Assessment Study. J Acad Nutr Diet. 2017;117:1265–71.  88. Croteau J, Guinhouya BC, Bujold E, Fraser WD, Marc I. Physical activity during pregnancy and infant’ s birth weight: results from the 3D Birth Cohort. BMJ Open Sport Exerc Med. 2017;3:1–9.  89. Zhang T, Wang P, Liu H, Wang L, Li W, Leng J, et al. Physical Activity, TV Watching Time, Sleeping, and Risk of Obesity and Hyperglycemia in the Offspring of Mothers with Gestational Diabetes Mellitus. Sci Rep. 2017;7(January):1–9.  90. Nguyen CL, Thi P, Nguyen H, Chu TK, Ha AV Van, Pham NM, et al. Cohort profile: maternal lifestyle and diet in relation to pregnancy, postpartum and infant health outcomes in Vietnam: A multicentre prospective cohort study. BMJ Open. 2017;7:1–9.  91. Wasenius NS, Grattan KP, Harvey ALJ, Barrowman N, Goldfield S, Adamo KB. Maternal gestational weight gain and objectively measured physical activity among offspring. Plus One [Internet]. 2017;12(6):1–14. Available from: http://dx.doi.org/10.1371/journal.pone.0180249  92. Watson ED, Brage S, White T, Westgate K, Norris S, Poppel M Van, et al. The Influence of Objectively Measured Physical Activity During pregnancy on maternal and birth outcomes in Urban black south african women. p. 22(8):1190-1199.  93. Dahly DL, Li X, Smith HA, Khashan AS, Murray DM, Kiely ME, et al. Prenatal and Early Life Exposures Associations between maternal lifestyle factors and neonatal body composition in the Screening for Pregnancy Endpoints (Cork) cohort study. Int J Epidemiol. 2018;47(1):131–45.  94. Pradeilles R, Allen E, Gazdar H, Mallah HB, Budhani A, Mehmood R, et al. Maternal BMI mediates the impact of crop- related agricultural work during pregnancy on infant length in rural Pakistan: a mediation analysis of cross-sectional data. BMC Pregnancy Childbirth. 2019;19:1–13.  95. Mcdonald SM, Liu J, Wilcox S, Sui X, Pate RR. Maternal physical activity prior to and during pregnancy does not moderate the relationship between maternal body mass index and infant macrosomia. J Sci Med Sport [Internet]. 2019;22(2):186–90. Available from: https://doi.org/10.1016/j.jsams.2018.07.013  96. Salas LA, Baker ER, Nieuwenhuijsen MJ, Marsit CJ. Maternal swimming pool exposure during pregnancy in relation to birth outcomes and cord blood DNA methylation among private well users. Environ Int [Internet]. 2019;123(Feb):459–66. Available from: https://doi.org/10.1016/j.envint.2018.12.017  97. Haakstad LAH. Pregnancy and advanced maternal age — The associations between regular exercise and maternal and newborn health variables. Acta Obs Gynecol Scand. 2020;99(2):240–8.  98. Ueno M, Takagi K, Tachibana Y, Morita Y, Nagano H, Muraoka M, et al. Quantitative analysis of exercise among pregnant women with impaired glucose tolerance using pedometer data: An observational study. J Obstet Gynaecol Res. 2019;46(3):396–404.  99. Nagpal TS, Prapavessis H, Campbell CG, Vrijer B de, Bgeginski R, Hosein KS, et al. Sequential Introduction of Exercise First Followed by Nutrition Improves Program Adherence During Pregnancy: a Randomized Controlled Trial. Int Soc Behav Medicien. 2019;12(12):1–11.  100. Lawani. Effect of antenatal gymnastics on childbirth: A study on 50 sedentary women in the Republic of Benin during the second and third quarters of pregnancy. Octobre-Novembre-Decembre. 2003;13(4):1–9.  101. Mottola MF. Exerc ise Prescription for Over weight a nd Ob es e Women: Pregnancy and Postpartum. Obs Gynecol Clin N Am. 2020;36(2009):301–16.  102. Kaminski SL, Harris EK, Berg EP, Vonnahme KA. Domestic Animal Endocrinology Impact of maternal physical activity during gestation on porcine fetal, neonatal, and adolescent ovarian development. Domest Anim Endocrinol [Internet]. 2014;48:56–61. Available from: http://dx.doi.org/10.1016/j.domaniend.2014.01.006  103. Fernández-castillo R, José M. Influence of physical exercise during pregnancy on newborn weight: a randomized clinical trial. Nutr Hosp. 2017;34(4):834–40.  104. Ferrari N, Bauer C, Janoschek R, Koxholt I, Mahabir E, Appel S, et al. Exercise during pregnancy and its impact on mothers and offspring in humans and mice. J Dev Orig Health Dis. 2020;9(2018):63–76.  105. Beeson JH, Blackmore HL, Carr SK, Dearden L, Duque-guimarães DE, Kusinski LC, et al. Maternal exercise intervention in obese pregnancy improves the cardiovascular health of the adult male offspring. Mol Metab [Internet]. 2018;16(June):35–44. Available from: https://doi.org/10.1016/j.molmet.2018.06.009  106. Mparmpakas D, Goumenou A, Zachariades E, Pados G, Gidron Y, Karteris E. Immune system function, stress, exercise and nutrition profile can affect pregnancy outcome: Lessons from a Mediterranean cohort. Exp Ther Med. 2013;5(2):411–8.  107. Pivarnik JM. Maternal Exercise During Pregnancy. Sport Med. 1994;18(4):215–7.  108. Garber CE, Blissmer B, Deschenes MR, Franklin BA, Lamonte MJ, Lee I-M, et al. Quantity and Quality of Exercise for Developing and Maintaining Cardiorespiratory, Musculoskeletal, and Neuromotor Fitness in Apparently Healthy Adults: Guidance for Prescribing Exercise. Med Sci Sports Exerc. 1995;5(5):634–40. | |

**Table S5 Characteristics of pregnant women in randomized controlled trials in meta-analysis**

| **Study** | **Country** | **Cohort recruited (n)** | **Cohort enrolled (n)** | **Study time** | **Gestation wk. at enrolled** | **Exercise duration** | **Age** | **BMI**  **(kg/m^2^)** | **Parity** | **Prep wt. (Maternal) C vs. E** | **Gestation wk.** | **Wt. gain** | **Height** | **outcome assessment** | **Exercise protocol** |
| --- | --- | --- | --- | --- | --- | --- | --- | --- | --- | --- | --- | --- | --- | --- | --- |
| Pomerance,1974 (1) | US | 54 | 39 | October 1, 1972 - Feb 28, 1973 | NA | NA | 17-30 | 18.5-24.9 | 0 or 1 | 57.8±3.4 vs. 58.9±6.4 or 66.9±1.1 vs. 58.7±6.2 | 38-41 | 15.6±0.8 vs.12.5±4 or 11.1±5.1vs. 13.3±4.1 | 175.6±1.3 vs. 169.9±7.3 or 169±6.6 vs. 175.4±5.7 | Researcher | Bicycle ergometer set at 450 k.p.m (1kpm = 9.8 joules); ); subject was asked to pedal 60 times /min; 3 section-1) 6 to 8 min - maternal pulse steady-record; else 2) pulse < 130; repeat at 600 k.p.m after 10 min rest- 8 min test - if pulse <= 180 beats/min-record; else 3) test at 300 k.p.m-record (pulse = average of last two min pulse) |
| Hall,1987(2) | US | 845 | 845 | NA | NA | Whole pregnancy | 30 vs. 26.2 or 24.5 or 25.2 | 18.5-24.9 | 0.9 vs. 0.6 or 0.7 or 0.8 | NA | 39.9 vs. 40.1 or 40.4 | NA | NA | Physician | warm-up-5min on treadmill (3 to 4 mph) or on exercise bicycle (300 kpm); workouts on exercise machines for 45 min; aerobic workout (300 kpm - 2 miles-85% of maximum (< 140 bpm); 3 days/ wk to labor or medical condition; each 10 wk schedule; 28 wk -1 session and another in 38 wk |
| Briend,1980(3) | Africa | 2307 | 2307 | NA | NA | Whole pregnancy | NA | 18.5-24.9 | NA | NA | NA | NA | NA | NA | mill workers |
| Dale,1982(4) | US | 115 | 115 | NA | NA | Whole pregnancy | 30 | 18.5-24.9 | 0.2 | 49.9 | 40.3 | 11 | 165.1 vs. 162.6 | NA | running |
| Clapp,1984(5) | US | 336 | 218 | January 1 and March 31, 1981 | Wk1 | Whole pregnancy | 28.4±0.8 vs. 28.4 ±0.8 or 28.2 ±0.6 | 18.5-24.9 | 0.67 | 61.9±1.1 vs. 59.7±1.2 or 59±2 | 40.1±0.2 vs. 40.3±0.3 or 39.1±0.3 | 14.6±0.4 vs. 16.8±0.8 or 12.2±0.6 | NA | Self-report | endurance-minimal-frequency of 3 times/wk & duration >30min & an intensity > 50% of max heart rate; high endurance - frequence of >6 times/wk & duration of > 1h & an intensity > 50% of max heart rate |
| Beckmann,1990(6) | US | 150 | 100 | NA | Wk1 | Whole pregnancy | 30.2±3.4 vs. 28.9±1.8 | 18.5-24.9 | 0 | NA | 40±1.1 vs. 39.8±0.8 | 13.1±2.7 vs.11.7±1.6 | NA | Self-report | Jogging and aerobic activity-for 1hr for twice /wk for minimum of 12 wks |
| Clapp,1990(7) | US | 132 | 100 | July 1983 to January 1990 | Wk1 | Whole pregnancy | 30±3 vs. 31±3 | 18.5-24.9 | 1 | 58±6 vs. 58±5 | 40±1 | 16±4 vs. 14±4 | NA | Obstetrician | > 30 min 3-4 times/wk before conception; delivered at term after exercise or above 50% of preconception level; |
| Klebanoff,1990(8) | US | 8287 | 7100 | Nov. 1984 to June 1987 | Wk1 | Whole pregnancy | 25-29 | 18.5-24.9 | NA | NA | >37(73-77%) | NA | NA | Self-report | Standing |
| Homer,1990(9) | US | 12686 | 773 | 1979 - 1983 | Wk1 | Whole pregnancy | 21.9 vs. 21.5 | 18.5-24.9 | 68.3%(0) | 57.1±0.6 | NA | 14.4 | 163.6±0.3 vs.  163.1±0.3 | Self-report | work related exertion jobs |
| Botkin,1991(10) | US | 62 | 46 | January 1,1989 and June 1, 1989 | Wk1 | Whole pregnancy | 27.2±5.5 vs. 28.1±5.1 | 25±4.8vs. 23±2.6 | Median 2 | NA | 40.1±1 vs. 40.5±0.9 | 14.5±3.5 vs. 14.4±4.1 | NA | Self-report | minimum 20 min for 3 times/wk & 20 wks of pregnancy- Guidelines for Exercise Testing and Prescription |
| Rose,1991(11) | Maine | 23091 | 21342 | June 1984 to June 1988 | Wk1 | Whole pregnancy | 25.7 vs. 25.5 or 26.6 | 18.5-24.9 | 39.9%(0) vs. 36.7% (0) or 37.9% (0) | 67.5±0.6 vs. 65.7±0.5 or 63±0.6 | NA | NA | 163.3±1.6 vs. 163.6±1.6 or 164.1±1.6 | Self-report | exercise level as light, vigorous, and moderate |
| Zeanah,1992(12) | US | 173 | 136 | NA | Wk1 | Whole pregnancy | 20-40 | 18.5-24.9 | 47% (0) | NA | NA | 14.9±1vs.  14.2±1 or 13.2±0.9 | NA | Self-report | ACOG-exercise through 3rd trimester |
| Hatch,1993(13) | US | 876 | 462 | January 1987 to June 1989 | Wk13 | 23 | 27.1±4.4 vs. 27.9±4.6 | 18.5-24.9 | 30.3 %(0);48.1%(1) vs. 44.8% (0);36.8%(1) | 61.9±12.9 vs. 61.6±13.9 | NA | NA | NA | Self-report | 6 - 12 kcal/minute-strenuous exercise; 3.5 - 5.5 kcal/min-less strenuous (low-<1000kcal/wk; heavy->1000 kcal/wk) |
| Johnson,1994(14) | US | 234 | 188 | NA | Wk1 | Whole pregnancy | 16 - 35 | 18.5-24.9 | 0 | NA | 40.1±0.1 vs. 40.3±0.1 | NA | NA | Self-report | Exercise |
| Sternreld,1995(15) | US | 529 | 338 | March 1989 to February 1991 | Wk1 | Whole pregnancy | 31.7 | 18.5-24.9 | 51.5%(0) | NA | NA | NA | NA | Self-report | level 1-aerobic exercise(no vigorous) 20min for > 3 times/wk; level 2-aerobic exercise -vigorous->20min for > 3 times/wk; level 3-aerobic exercise-20 min for < 3 times/wk; level 4-aerobic exercise <1 time/wk |
| Henriksen,1995(16) | Denmark | 8711 | 4249 | August 1989-september 1991 | Wk1 | Whole pregnancy | majorly (25-34); 12.4%(<25); 14.7%(>35) | 18.5-24.9 | 50% (0);34.7%(1); 15% (>=2) | 50-69 | NA | NA | 160-169 | Self-report | standing or walking or lifting |
| Bell,1995(17) | Victoria | 99 | 99 | NA | Wk1 | Whole pregnancy | 31.6±4.7 vs. 31.8±2.7 | 18.5-24.9 | 39%(0) vs. 54%(0) | 59.1±7.6 vs. 58.8±6.9 | NA | NA | 165±8 vs. 166±6 | Self-report | Exercise vigorously-aerobics, swimming, running; > 3 times/wk-(>30min each); |
| Clapp,2000(18) | US | 50 | 46 | NA | Wk1 | Whole pregnancy | 31±1 | 18.5-24.9 | 0.4 | 61.7±1.3vs. 62.1±1.1 | 39.7±0.3 vs. 39.6±0.3 | 16.3±0.7vs.  15.7±1 | NA | Objectively | weight -bearing exercise-treadmill; step aerobics; or stair stepper-20min (3-5 times/wk); intensity-55% to 60% of the preconception maximum aerobic capacity |
| Bell,2002(19) | Australia | 64 | 61 | NA | Wk25 | To the end of pregnancy | NA | 18.5-24.9 | NA | NA | NA | NA | NA | Self-report | exercise at least 5times/wk to 3 or fewer wk |
| Leiferman,2003(20) | US | 13417 | 9089 | 1988 | Wk1 | Whole pregnancy | 16.7% (<20); 73.5% (20-34 ); other > 34 | 67.8% (<24); 24 %(25-29); other (> 30) | NA | NA | NA | NA | NA | Self-report | Exercise or sport >3times/wk |
| Takito,2005(21) | Brasil | 220 | 152 | March 1997 to October 1998 | Wk16 | 19 | 24.4 | 24.4±5.1 vs. 23.8±4.6 | NA | 53.3%(1); 27.6%(2); 12.5%(3); 6.6% (>=4) | NA | NA | 158.7±5.6 | Self-report | walking; standing; washing clothes by hand |
| Suezanne,2006(22) | Georgia | 1163 | 922 | 1993- 1995 | Wk1 | Whole pregnancy | 75.6%(>20); 14.4% (18-19) | 18.5-24.9 | NA | NA | NA | NA | NA | Self-report | ACOG guidelines or Bell et al standards |
| Duncombe,2006(23) | Australia | 148 | 148 | NA | Wk16 | 22 | 31.7 | 23.8 | 45.1% (0); 35.8% (1);9.3% (2); other 2.5% | NA | 39±2.4 vs. 39.5±1.9  or40.1±1.1 or39.6±1.6 or39.7±1.4  or39.8±1.6 | NA | NA | Self-report | ACOG-1985: 3-4 vigorous sessions |
| Dwarkanath,2007(24) | India | 923 | 546 | NA | Wk12 | 22 | 24.4±4.1 | 22±3.9 | 59.9 % (0); 37.4% (1-2); 15% (>=3) | 52.6±9.6 | NA | NA | 155±6 | Self-report | occupational activity outside house; discretionary exercise; household chores; sedentary activity; hobbies and sleep |
| Snapp,2008(25) | Georgia | 105600 | 75160 | 1988 | Wk1 | Whole pregnancy | 2.4% (<20); 53.7% (20-29); 38.2% (30-39); 5.7%(>40) | 7.3%(<18.4); 50.6%(18.5 - 24.9); 23.9% (25 - 29.9);  18.3% (>30) | NA | NA | NA | 11.4±0.3 vs.  11.5±0.3 | NA | Self-report | ACOG-30 min- >3times/wk-for 6 or more months of pregnancy |
| Mileur,2008(26) | US | 86 | 22 | July 2000 to June 2004 | Wk1 | Whole pregnancy | NA | 18.5-24.9 | NA | NA | 29.6±1.5 vs. 28.7±1.6 | NA | NA | Self-report | Physical exercise |
| Elden,2008(27) | Sweden | 386 | 259 | August 2000 to May 2002 | Wk12 | 19 | 30.4±4.7 vs. 29.8±4.2 | 18.5-24.9 | 25.6% (0) vs. 27.7% (0) | NA | 39.5±1.6 vs. 39.7±1.6 | NA | NA | Self-report | Stabilizing exercise |
| Owe,2009(28) | Norway | 40049 | 36869 | June 1, 2001 and May 31, 2005 | Wk17 | 13 | 23.3% <25; 44.6% (25-29); 25.4% (30-34); 6.6% (>35) | 3.3% (<18.5); 63.6%(18.5-24.9); 20.6%(25-29); 6.5%(30-34); 2.3%(>35) | 0 | NA | NA | 9.5±4.7 | 168.2±6 | Self-report | strolling, brisk walking, running, bicycling, fitness training, swimming, aerobic classes, skiing, ball games, horseback, riding and other |
| Barakat,2009(29) | Sweden | 480 | 142 | January 2000 to March 2002 | Wk12-13 | 26 | 29.5±3.7 vs. 30.4±2.9 | 23.4±0.5 vs. 24.3±0.5 | 57.1% (0); 35.7% (1) vs. 72.2% (0); 22.2% (1) | 60.1±9.8 vs. 64.7±11.6 | 39.7±1.3 vs. 39.6±1.3 | NA | 160±5 vs. 163 ±5 | Self-report | light resistance and toning exercise(>3time/wk-35-40 min) |
| Vrijkotte,2009(30) | Netherlands | 12373 | 7730 | January 2003 to March 2004 | Wk13 | To the end of pregnancy | 30.7±4.8 | 23.3±3.7 | 50.7%(0); | NA | 40±1.2 | NA | 168±2 | Self-report | Working conditions-standing/walking; physical workload and other |
| Hegaard,2010(31) | Denmark | 58530 | 4458 | August 1989 to September 1991 | Wk16 | To the end of pregnancy | 1.4% (<20); 18% (20-24); 44.6% (25-29);27.3% (30-34);other | 7.7%(<18.5); 80.3%(18.5 - 24.99);8.8%(25-30);2.7(>30) | 55.5%(0); 33.7%(1); 10.7%(2);  2.24%(3) | NA | 3.5%(37wk);8.3%(38wk); 21.3%(39wk);32.3%(40wk); 25%(41wk);11.7%(42+) | NA | NA | Self-report | sports activities - non-weight carrying sport; weight carrying sport; and other |
| Fleten,2010(32) | Norway | 52547 | 43705 | 1999 to 2006 | Wk17 | 13 | 12.9%(15-24);36.3%(25-29);37.1%(30-34);12.4% (35-39);1.3% (<49) | 2.9%(<18.5);65% (18.5-24.9);22.3% (25-29.9);7.1%(30-34.9);2.7% (>35) | 44.8% (0) | NA | NA | NA | NA | Self-report | walking, running,  bicycling, fitness, weight training, prenatal aerobic classes, aerobic classes, dancing, skiing, ball games, swimming |
| Hopkins,2010(33) | New Zealand | 257 | 84 | December 2004 to May 2007 | Wk19-20 | >16 | 29±4 vs. 31±3 | 25.5±2.9 vs. 26.7±3.3 | 0 | NA | NA | NA | NA | Self-report | Exercise-moderate exercise intensity of 65% predicted aerobic capacity (VO2max)-max-5 session-40 min/wk; exercise was maintained >36 wk gestation |
| Mottola,2010(34) | Canada | 325 | 325 | NA | Wk16-20 | To the end of pregnancy | 32.1±3.2 vs. 31.8±3.6 or 31.8±3.6 vs. 32.8±4.3 | 27.4±1.7 vs. 27.4±1.6 or 36.2±4.8 vs. 36.4±5.2 | 55% (=>1) or 68%(>=1) | 74.3±7.4 vs. 75.8±6.7 or 96.8±14.2 vs. 99.8±16.8 | 39±1.4 vs. 39.6±1.1 or 39.1±2.2 vs. 39±1.8 | 13±4.5 or 11±6.5 | NA | Self-report | Physical activity |
| Price,2012(35) | US | 94 | 62 | July 2006 to March 2010 | Wk12-14 | >24 | 27.6±7.3 vs. 30.5±5 | 28.7±5.4 vs. 26.6±3.1 | 0.7 vs. 0.5 | NA | NA | NA | NA | Objectively measured | ACOG-Exercise-aerobic training of 45-60 min-4times/wk-moderate intensity (12-14 on borg scale of perceived exertion). |
| Haakstad,2011(36) | Norway | 105 | 105 | September 2007 to March 2008 | Wk12 | ~28 | 30.3±4.4 vs. 31.2±3.7 | 23.9±4.7 vs. 23.8±3.8 | NA | 68.4±14.6 vs. 67.9±11.4 | 39.6±1.2 vs. 39.9±1.2 | NA | 169±10 | Investigator measured | ACOG-> 2 out of 3 possible 1hr aerobic dance classes/wk(>12 wk)-[5min warm up; 35 min aerobic dance; + cool down.Last 5 min-stretching, relaxation and body awareness exercise. |
| Jahromi,2011(37) | Iran | 142 | 132 | April 2007 to April 2008 | Wk1 | Whole pregnancy | 23±4.8 vs. 23±5.2 | 24.2±4.7 vs. 22.4±3.6 | 0 | 59.5±9.2 vs. 63.5±12.4 | 38.2±2 vs. 39.2±1.3 | 14.1±2.2 or 13.8±4.3 | 162±6.8 vs. 163±6.6 | Self-report | Physical exercise-standing; walking or aerobics > 2times/wk >30min each |
| Salonen,2011(38) | Finland | 606 | 581 | 2001-2004 | NA | NA | NA | 26 | NA | NA | NA | NA | NA | Self-report | Walk test-UKK 2km walk test-maximal exercise stress test |
| Nascimento,2011(39) | Brazil | 93 | 80 | August 2008 to March 2010 | Wk1 | Whole pregnancy | 30.9±5.9 vs. 29.7±6.8 | 36.4±6.9 vs. 34.8±6.6 | NA | 94±19.2 vs. 92.6±18.9 | 38.5±1.5 vs. 38.5±2.6 | 11.5±7.4 or 10.3±5 | 160±6 vs. 163±7 | Objectively measured | ACOG- Exercise under supervision and received home exercise counselling |
| Jukic,2012(40) | US | 1647 | 1647 | 2004 - 2007 | Wk10 | To the end of pregnancy | 25-34 | 2% (<18.5); 60% (18.5 - 29.9); 22% (25-29.9); 16% (>=30) | NA | NA | 39.6±1.9 | NA | NA | Self-report | recreational-occupational-indoor/outdoor household and child/adult care -lifting or carrying boxes and lifting and transporting patients |
| Melo,2012(41) | Brazil | 281 | 187 | May 2008 to Sep. 2010 | Wk13-20 | To the end of pregnancy | 24±5.4 vs. 24±5.8 | 23.5±3.5 vs. 24.7±4.3 or 23.4±3.8 | 1 or 0 | NA | NA | NA | 157±6 vs. 156±6 | Objectively measured | initial duration of walking-15 min; increase over; intensity- heart rate between 60% and 80% of max-in open air-with temperature of 24 degree C; |
| Mudd,2012(42) | US | 3019 | 1014 | 1998 - 2004 and 2007 | Wk1 | Whole pregnancy | 5.5-10% (<20); 48.3 - 58.1 % (20-30); 31.9-46.2% (>30) | 48.5 - 55.5% (<25); 23.3 - 26.8% (25-30); 17.7 - 28.2 %(>30) | 41-44.5% (0) | NA | NA | NA | NA | Self-report | Leisure-time physical activity |
| Andersen,2012(43) | Denmark | 92274 | 40280 | 1996 - 2002 | Wk1 | Whole pregnancy | 30.8±4.2 vs. 30.4±4.1 | 23.4±4 vs. 23.2±3.8 | 48% (0) | NA | 40.2±1.3 | 15.4±5.5 vs. 15.1±5.0 | NA | Self-report | Recreational exercise-hr/wk 1- over 5 hr/wk |
| Oostdam,2012(44) | The Netherlands | 270 | 101 | January 2007 and January 2011 | Wk12 | To the end of pregnancy | 30.1±4.5 vs. 30.8±5.2 | 33.9±5.6 vs. 33±3.7 | 28% (0) vs. 38.3%(0) | NA | 39.4±1.7 vs. 39.6±1.0 | NA | NA | Objectively measured | Exercise program on 2 days of the wk during the remaining pregnancy; session-60 minutes; aerobic + strength exercises- |
| Krogsgaard,2013(45) | Norway | 3739 | 2026 | 1984 – 1986 (Hut 1); 1995 - 1997 (Hunt 2); 2006 - 2008 (Hunt 3) | Wk1 | Whole pregnancy | 26.8 vs. 27.2 or 26.7 or 26.6 | 22.4 vs. 22.3 or 22.4 or 22.5 | 29.4 % (0) vs. 28.2% (0) or 28.5% (0) or 40.6% (0) | NA | NA | NA | NA | Self-report | Exercise session - walking, skiing, swimming, or other/wk; 1-5 times/wk; duration-15 - 60 min; intensity-light, moderate; exhaustion |
| Rauh,2013(46) | Germany | 250 | 226 | February 2010 and August 2011 | NA | Whole pregnancy | 30.8±4.9 vs. 32.2±4.4 | 22.8±3 vs. 21.7±1.9 | 68.7 % (0) vs. 65.9% (0) | 63±5 vs. 62±3 | NA | 15.6±5.8 vs. 14.1±4.1 | 168±6 vs. 169±6 | Objectively measured | Physical activity |
| Tomić,2013(47) | Croatia | 334 | 334 | July 2008 and December 2009 | Wk6-8 | 31~33 | 29.2±3.2 vs. 28.9±0 | 22.8±4.5 vs. 23.1±4.1 | 35.7%(0);45.8%(1); 14.9%(2); 3.6%(>=3)or  45.2% (0); 40.4%(1);10.8%(2); 3.6%(>=3) | 64.4±11.1 vs. 64.8±13.4 | 39.1±0.6 vs. 38.8±0.4 | 15.7 vs. 13.4 | 168.2±6.3 vs. 167.5±6 | Objectively measured | ACOG- aerobic exercise-warm up-5 min; aerobic exercise-30 min; stretching 10 min; cool down period 5min; 3times/wk during whole pregnancy period |
| Barakat,2014(48) | Spain | 282 | 200 | NA | Wk10-12 | 27-29 | 31.5±3.9 vs. 31.6±3.9 | 24.1±4.3 vs. 23.8±4.4 | 53.9%(0);40.4% (1); 5.6%(>=2) vs. 60.7 % (0); 34.6%(1); 4.7%(>=2) | NA | 39.2±2.2 vs. 39.5±1.9 | 13.7±9.6 vs. 11.7±4.1 | NA | Objectively measured | ACOG- Exercise-3 times/wk-55 to 60 min sections; between wk 9 and wk 13 of gestation and to end of pregnancy |
| Przybytowicz,2014(49) | Poland | 607 | 510 | February 2010 and November 2012 | Wk1 | Whole pregnancy | 28.1 | 22.5±3.7 vs. 20.6±2 or 22.1±4.2 or 22.6±3.7 | 87.1% (0) | 61.8±10.3 vs. 58.2±6.9 or 62.7±14.3 or 62.1±10.9 | >37 | 15.3±4.9 vs. 14.5±4.6 or 15.7±6.1 or 15.9±6.2 | 166±5.8 vs. 167±4.9 or 167.9±5.3 or 167.3±5.5 | Self-report | active-daily physical activity>30 min; Moderate active- physical activity 2-3 times/wk and >30 min each time; light activity-physical activity 1/wk for 30 min |
| Kong, 2014(50) | US | 46 | 34 | NA | Wk15 | To the end of pregnancy | 18-48 | >25 | NA | NA | NA | NA | NA | Self-report | 2008 US physical activity guidelines for pregnant population- min 150 min/wk of moderate physical activity; |
| Reid,2014(51) | UK | 175 | 100 | July 2009 to December 2010 | Wk1 | Whole pregnancy | 30.1±4.9 vs. 31.8±4.3 | 25.2±5.2 vs. 27.6±4 | 35 %(>1) vs. 98% (>1) | 67.3±14.5 vs. 75.4±12.8 | 39.7±1.3 vs. 39.4±1.3 | 19.9±7.9 vs. 19.7±7.9 | 163±6.4 vs. 165±5.9 | Self-report | free living physical activity and sedentary behavior |
| Tanvig,2014(52) | Denmark | 1224 | 157 | October 2007 to October 2010 | Wk10-14 | To the end of pregnancy | 30±0.5 vs. 30.5±0.8 | 34.3±0.7 vs. 33.4±0.7 | 56 % (0) vs. 51.2% (0) | NA | 40.1±0.7 vs. 39.9±1.1 | 8.8±7.9 vs. 7.7±0.9 | NA | Self-report | encouragement to be moderately physical active for 30 -60 min/day |
| Li,2014(53) | China | 239 | 118 | July 2012 to June 2013 | Wk1 | Whole pregnancy | 26±3 vs. 26±4 | 18.5 ~ 23.9 | 0 | NA | NA | NA | 162.7±2.2 vs. 163.8±1.6 | Self-report | Walking - v-50 m/min; 4 timse/wk; >30 min per session; |
| Ghods,2014(54) | Pakistan | 80 | 80 | January to July 2011 | Wk20-26 | To the end of pregnancy | 23.3 vs. 23.4 | 22.7 | NA | NA | >38 | 10.7±3.4 vs. 9.4±2.1 | NA | Self-report | Bicycle ergometer for 15 min-3 times/wk- intensity of 50-60% of maximal heart rate |
| Tanvig,2014 (55) | Denmark | 1224 | 150 | 2007 to 2010 | Wk10-14 | To the end of pregnancy | 30.3±3.4 vs. 30.5±2.6 | 33.4±2.5 vs. 33.3±2.1 | 57.5 % (0) vs. 54.5% (0) | NA | 40.6±0.9 vs. 40.2±1.0 | 8.8±2.9 or or17±3 vs. 7±1.8 | NA | Objectively measured | physical activity - encouragement to be moderately physically active-30 to 60 min/d |
| Currie, 2014(56) | Canada | 2200 | 1749 | October 2002 to July 2005 | Wk20 | To the end of pregnancy | 9.3% (<25); 72.6% (25-35); 18.2 % (> 35) | 3.7% (<18.5); 55.7% (18.5 - 24.9); 22.2 (25- 29.9); 18.4 %(>=30) | 56.7% (0) vs. 46.6%(0) | NA | 39±1.9 | NA | NA | Self-report | Physical activity-active living; active transport; decrement for television watching; duration and intensity (up to 15 items). |
| Dodd,2014(57) | Australia | 5474 | 2142 | June 2008 and December 2011 | Wk14 | To the end of pregnancy | 29.6±5.6 vs. 29.3±5.4 | 31.1±4 vs. 31±3.9 | 40.2 % (0) vs. 41.4 % (0) | NA | 39.2±2.1 VS. 39.3±1.7 | NA | 164.8±16.5 vs. 164.9±16.4 | Self-report | physical activity advice primarily encouraged women to increase their amount of walking and incidental activity |
| Mudd,2015(58) | South Korea | 3032 | 940 | 1998 to 2004 | Wk25 | To the end of pregnancy | 57.1 % (20 - 30); 9.3% (<20); 33.6%(>30yr) | 49.4% (<25); 22%(25 - 30); 28.5% (>30) | NA | NA | NA | NA | NA | Self-report | Physical activity |
| Vamos,2015(59) | US | 2684 | 1713 | 1994-1995; 2001-2002; 2007-2008 | Wk1 | Whole pregnancy | 21.8±0.1 | NA | NA | NA | NA | NA | NA | Self-report | physical activity-roller blading, roller - skating, skate - boarding, or bicycling; play an active sport- baseball; exercise- jogging, walking and other; 5 or more in the past wk; sports- running; gymnastics-weight lifting and others |
| Wang,2015(60) | China | 14168 | 14168 | June 20th to November 30th, 2013 | Wk25.8 | To the end of pregnancy | 28.7±4.4 vs. 29.4±4.4 | 22.5±3.7 vs. 22.7±3.6 | 70.4% (0) | NA | NA | NA | NA | Self-report | physical activity-light- no work; sitting; walking<60 min/d; moderate-physical activity makes a pregnant women breather harder than normal (cooking, sweeping the floor, washing, >60 min-walking; high-require considerable physical effort-breathe much harder than normal-lifting, aerobics, bicycling, dancing, swimming). |
| Dodd,2016(61) | Australia | 5474 | 970 | June 2008 and December 2011 | Wk14 | To the end of pregnancy | 29.8±5.2 vs. 29.8±5.6 | 31.2±4.3 vs. 31.3±3.9 | 43.2 % (0) vs. 42.2 % (0) | NA | NA | NA | 164.6±6.5 vs. 164.6±6.4 | Objectively measured | exercise-focused on walking and incidental activity |
| Ronnberg,2016(62) | Sweden | 445 | 374 | June 2007 to Sep. 2009; follow up 2015 | Wk16 | To the end of pregnancy | 29.8±4.8 vs. 29.9±4.5 | 25.3±4.8 vs. 25.2±4.9 | 51%(0) vs. 44% (0) | NA | 39.4±1.2 vs. 39.7±1.2 | NA | NA | Objectively measured | Exercise |
| Lindqvist,2016(63) | Sweden | 3868 | 3762 | 2011 and 2012 | Wk1 | Whole pregnancy | 30±5 | 25±5 vs. 24±4 | 2 | NA | 40±1 | NA | 166±7 vs. 166±6 | Self-report | low-not breathless or sweaty; moderate - warm; vigorous-high pulse, breathless, and sweaty |
| Daly,2016(64) | Ireland | 166 | 155 | July 2009 to March 2010 | Wk1 | Whole pregnancy | 31.5±5.5 | 24.6±5 | 43% (0) | NA | NA | NA | NA | Self-report | Toning, weights, horse riding, dancing, sports, swimming, yoga, aerobics, spinning, bicycle, jogging, walking (80%) |
| Rego,2016(65) | Brasil | 1380 | 1380 | 2009 - 2011 | Wk1 | Whole pregnancy | 80.9 %(20-34); 6.9%(>=35); 12.3% (<20) | NA | 51.5%(1); 48.5 % (>=2) | NA | NA | NA | NA | Self-report | walking-1MET = 3.3 x time (min)*d; moderate activity-1MET = 4.0 x time(min)*d; high activity 1MET = 8.0 x time (min) x d |
| Silva,2017(66) | US | 2902 | 1561 | January 1st 2015 to March 2016 | Wk16-20 | >16 | 27.1±5.7 vs. 27.2±5.3 | 25.2±4.1 vs. 25.1±3.9 | 66.1 (0) vs. 64.9% (0) | NA | 38.7±1.8 vs. 38.8±2 | 8.4±3.5 vs. 7.8±3.5 | 161±6 vs. 161±6.6 | Objectively measured | structured -supervised- moderate intensity exercise- 1hr 3d/wk; warm up, aerobic activities, strength training, stretching exercise; a mean of 48 sessions were planed; wk1-4-5 min warm up-15min aerobic exercise-35 min strength training, and 5 min stretching; the second stage (wk 5-10)- 5 min warm up+20 min aerobic exercise+30 min strength training+5 min stretching; lastly- 5 min warm up+25 min aerobic exercise+25 min strength training+5 min stretching. |
| Norris,2017(67) | Ireland | 1200 | 1200 | February 2007 to August 2011 | Wk15 | To the end of pregnancy | 30.5±4.2 vs. 28.9±4.7 or 28.9±5.2 or 29.5±4.6 or 30.4±4.3 | 18.5-24.9 | NA | NA | 40±1.2 | NA | NA | Objectively measured | vigorous activity-(breathe harder or puff or pant); moderate activity (not breathe harder or puff or pant); walking for recreation or exercise |
| Díaz,2017(68) | Spain | 105 | 105 | April to June 2016 | Wk26-28 | 8 | 31.5±5 vs. 32.9±4.5 | 26.8±5 vs. 28.8±4.3 | 78 % (0) vs. 68 % (0) | 72.8±14.1 vs. 76.6±12.1 | NA | NA | 164±5 vs. 163±5 | Objectively measured | 8 wk program of physical exercise-Pilates method- 2 times/wk and 40 - 45 min per session |
| Huang,2018(69) | China | 1970 | 1970 | September 2009 to March 2011 | Wk1 | Whole pregnancy | 28±4.3 vs. 27.6±4.5 | 26.6% (<18.5);62.9 % (18.5 - 23.9); 8.7% (>=24); vs. 33.4% (<18.5); 54.3 %(18.5 - 23.9); 7.1% (>24) | 76 % (0); 23%(>=2) vs. 80% (0);19.6%(>=2) | NA | 39±1.2 vs. 37.9±1.1 | NA | NA | Self-report | Exercise |
| Daly,2017(70) | Ireland | 337 | 88 | November 2013 to April 2016 | Wk12 | To the end of pregnancy | 29.4±4.8 vs. 30±5.1 | 34.7±5.1 vs. 34.7±4.6 | 43.2% (0) vs. 38.6% (0) | NA | 39.6±1.8 vs. 39.2±1.6 | NA | 164.6±12.2 vs. 165±6 | Objectively measured | three medically supervised exercise classes/wk |
| Wang,2017(71) | China | 821 | 226 | December 2014 to July 2016 | Wk27 | To the end of pregnancy | 32.5±4.9 vs. 32.1±4.6 | 26.8±2.8 vs. 26.8±2.7 | 80.7 % (0) vs. 80% (0) | NA | 38.9±1.4 vs. 39.0±1.3 | 10.5±3.3 vs. 8.4±3.7 | 162±4.9 vs. 162.4±5.1 | Objectively measured | 30 min-(5 min warm up at low intensity (55-65% age-predicted heart rate maximum (Hrmax)); a rating perceived exertion (RPE)); + 5 min continuous moderate-intensity cycling (65 - 75% of HR max); 30s rapid pedaling (sprints, higher intensity efforts) at 75 - 85% HRmax; every 2 min for 3-5 interval; + 5 min cycling at low to moderate intensity (60 - 70% HRmax); + another cycling; moderate-intensity cycling at 65 - 75% of HRmax (RPE 12-14) was interspersed with 1 min periods of pedaling against increased resistance (hill climb) at 75 - 85% of HRmax (RPE13-15); alternated every 2 min for 3 repeats; each session ended 5 min cool-down of easy cycling. |
| Bacchi,2017(72) | Canada | 184 | 111 | March 2013 to May 2015 | Wk8-11 | 25.8 | 31±5 vs. 30.4 ±4 | 24±4 vs. 23.1±3.2 | 71 % (0); 24.2%(1); 4.8% (>=2) vs. 79.6% (0); 16.3% (1); 4.1% (>=2) | NA | NA | 13.9±4.3 vs. 12.7±2.6 | NA | Objectively measured | Aquatic activities |
| Garnas,2017(73) | Norway | 136 | 74 | September 2010 to March 2015 | Wk11-14 | To the end of pregnancy | >=18 | >=28 | NA | NA | 39.5±1.3 vs. 39.1±2.3 | NA | NA | Objectively measured | Exercise training |
| Patel,2017(74) | UK | 1555 | 698 | July 2010 to May 2015 | Wk15 | To the end of pregnancy | 31±5.6 vs. 31.3 ±5 | 18.5-24.9 | 51.1% (0) vs. 50.6% (0) | NA | 39.6±2.3 vs. 39.7±1.5 | 7.8±4.4 vs. 6.9±4.7 | NA | Objectively measured | Physical activity-MET-2109 min/wk; MVPA 120 min/wk; walking 420 min/wk |
| Barakat,2017(75) | Spain | 998 | 568 | NA | Wk9-11 | 28-31 | 31.9±4.5 vs. 31.5 ±3.8 or 30.9 ±4 or 32 ±3.5 | 4.2% (<18.5); 65 % (18.5-24.9 kg/m2); 23.2 (25-29.9); 7.7% (>30) vs. 0.9% (<18.5); 68.2 %(18.5-24.9 kg/m2); 23.4 (25-29.9); 7.5% (>30) or 2.0% (<18.5); 65.3 %(18.5-24.9 kg/m2); 26.5 (25-29.9); 6.1% (>30) or 5.9% (<18.5); 73.3 %(18.5-24.9 kg/m2); 16.8(25-29.9); 4% (>30) | 56.6% (0);37.3 % (1); 6.1% (>=2) vs. 60.7 % (0); 34.6% (1); 4.7% (>=2) or 77.6 % (0); 18.4% (1); 4.1% (>=2) or 62.4 % (0); 29.7% (1); 7.9% (>=2) | NA | 39.4±1.4 vs. 39.5±1.9 or 39.2±1.0 or 39.7±1.2 | 13.4±4.6 vs. 11.7±4.2 | NA | Objectively measured | land-based exercise; aquatic exercise |
| Hegaard,2017(76) | Denmark | 1827 | 1762 | November 1996 to October 1999 | Wk1 | Whole pregnancy | 18.7%(<25); 43.1%(26-30);30.4% (31-35); 7.9% (>=36) vs. 6.5%(<25); 64.5%(26-30);19.8% (31-35); 9.2% (>=36) or 11%(<25); 84.4%(26-30);8.7% (31-35); 2.9% (>=36) or 8.3%(<25); 75%(26-30);16.7% (31-35) | 22±2 vs. 22.2±2.6 or 22±1.5 or 21.9±1.8 | 52.9 % (0) vs. 51.6 % (0) or 71.3% (0) or 80.6% (0) | NA | NA | NA | NA | Self-report | Physical activity |
| Badon,2017(77) | US | 2983 | 1408 | 1994-2008 | Wk1 | Whole pregnancy | 24±0.2 | 26±0.3 | 56% (0) | NA | NA | NA | NA | Self-report | leisure-time physical activity-jogging, walking, karate, jumping rope, gymnastics, dancing, rollerblading, roller skating, skate-boarding, bicycling, baseball, softball, basketball, soccer, swimming, football. -frequency |
| Dhana,2017(78) | US | 24289 | 16945 | 1996 - 2013 | Wk1 | Whole pregnancy | 41.3±4.3 | 25.1±5.2 | NA | NA | NA | NA | NA | Self-report | Physical activity |
| McDonald,2018(79) | US | 210 | 124 | November 2001 to July 2006 | Wk14 | To the end of pregnancy | 32.1±4.9 vs. 32.5±21.5 | 30.3±6.8 vs. 29.7±7.5 | NA | NA | 38.8±1.7 vs. 38.5±2.0 | 7±4.2 vs. 6±2.9 | 164±8 vs. 164±7 | Objectively measured | Moderate-intensity exercise |
| Badon,2018(80) | US | 5073 | 4434 | 1996-2008 | Wk15 | To the end of pregnancy | 33±5 vs. 33±4 | 2% (<18.5); 72 % (18.5 - 24.9 kg/m2),17%(25-29.9);9%(>30) vs. 2%(<18.5); 75 % (18.5 - 24.9 kg/m2),17%(25-29.9);7%(>30)  or 2%(<18.5); 80 % (18.5 - 24.9 kg/m2),14%(25-29.9);4%(>30) | 59 %(0) vs. 70% (0) or 61 % (0) vs. 73% (0) | NA | 39±2.0 | 14±5 | NA | Self-report | walking and yoga-early pregnancy or prepargnancy |
| Barakat,2018(81) | Spain | 92 | 65 | January 2014 and January 2016 | Wk9-11 | 27-31 | 33.8±2 vs. 33.1±3 | 24.4±6.0 vs. 24.1±3.9 | 59.4 %(0) vs. 69.7% (0) | NA | NA | NA | NA | Objectively measured | Exercise- 55-60 min- 3 times/wk-from beginning to end of pregnancy(wk 8-11 to wk 38-39); average 85 training; |
| Chan,2018(82) | China | 1217 | 172 | April 2015 to April 2017 | Wk12 | To the end of pregnancy | 33.1±4.1 vs. 33.2±4.4 | 24.1±4.0 vs. 23.1±3.9 | 58.1 % (0) vs. 61.2%(0) | 58.1±9.6 vs. 56.6±10.5 | 39.2±1.0 vs. 39.1±1.2 | NA | 157.8±5.1 vs. 158.2±5.5 | Objectively measured | Physical activity |
| Mizgier,2018(83) | Poland | 71 | 57 | 2014-2015 | Wk15-16 | To the end of pregnancy | 29±2.9 vs. 29.7±3.9 | 22.2±2.4 vs. 21.3±2.7 | NA | 63.4±6.5 vs. 62.6±9.1 | 39.3±2.1 vs. 40.1±1.4 | NA | NA | Objectively measured | ACOG- shorter moderate physical activity or longer moderate physical activity |
| Myrex,2018(84) | US | 179 | 179 | ~May 2016 | Wk1 | Whole pregnancy | 27.8±5.8 vs. 26±5.9 | 33.7±7.1 vs. 35.7±9.3 | 0 | NA | 38±2.0 | NA | NA | Self-report | Exercise |
| Poppel,2019(85) | European | 2009 | 334 | 2012- 2015 | Wk24-28 | To the end of pregnancy | 31.9±5.6 vs. 31.7±4.9 or 32.4±5.6 vs. 32.5±5.3 | 33.7±3.7 vs. 33.8±3.9 or  34.2±3.7 vs. 33.6±3.6 | 56 % (0) vs. 62% (0) or 45% (0) vs. 47%(0) | NA | 39.7±1.4 vs. 39.6±1.4  or 39.8±1.4 vs. 39.6±1.4 | NA | NA | Objectively measured | Physical activity |
| Hoffmann,2019(86) | Germany | 2286 | 1994 | 2013 - 2015 | Wk12 | To the end of pregnancy | 30.3±4.4 | 24.4±4.5 | NA | 68.2±13.4 | NA | NA | NA | Self-report | Physical activity |
| Brik,2019(87) | Spain | 120 | 85 | November 2014 to June 2015 | Wk16 | To the end of pregnancy | 32.7±4.4 vs. 33.4±3.2 | 24.3±5.3 vs. 23.4±3.6 | 74.4% (0) vs. 78.6% (0) | 65.5±15 vs. 63.5±10 | 39.6±1.4 vs. 39.7±1.3 | 11.2±6.4 vs. 11.4±4.2 | 164.2±6 vs. 164.6±6 | Objectively measured | 60 min/session for 3 times/wk (10 min-warming up+ 25 min of cardiovascular exercise + 10 min of strengthening exercises + 5 min of coordination + balance exercise + 5 min of coordination and balance exercise+5 min pelvic floor + 5 min stretching and relaxation) |
| Mcmillan,2019(88) | US | 121 | 60 | July 2015 and January 2018 | Wk13-16 | To the end of pregnancy | 29.5±4.4 vs. 30.7±3.4 | 25.7±4.8 vs. 24.8±5.2 | NA | NA | 38.9±1.2 vs. 39.5±1.5 | 9.6±5.7 vs. 15.3±11.4 | NA | Objectively measured | Exercise |
| Barakat,2019(89) | Spain | 594 | 456 | NA | Wk8-10 | To the end of pregnancy | 31±3.8 vs. 31.8±4.7 | 23.7±3.8 vs. 23.5±3.8 | 73 % (0); 24.3%(1); 2.7% (>=2) vs. 60.7% (0); 32.9%(1); 6.4% (>=2) | NA | 39.6±1.4 vs. 39.6±1.8 | 13.3±4.1 vs. 12.2±3.7 | NA | Objectively measured | Exercise intervention- 3 days/wk (55 - 60 min / session) from 8-10 wk of pregnancy to the end of the third trimester (wk 38 - 39). |
| Clark,2019(90) | USA | 47 | 36 | NA | Wk16 | To the end of pregnancy | 30.4 ± 4 vs. 28.3 ±4.2 | 28.1±8 vs. 24±5.2 | 0 | NA | NA | 15.2±10 vs. 13.4±4.5 | NA | Objectively measured | Exercise-5 min warm-up; 50 min exercise maintained in moderate intensity based on heart rate and rating of perceived exertion+ 3-5 min cool down; |
| Jochumsen,2019(91) | Denmark | 4008 | 4008 | July 1989 to November 1991 | Wk1 | Whole pregnancy | 19.8% (<25); 43% (25-29); 37.2%(>=30) vs. 13.3% (<25); 42.7% (25-29); 44%(>=30) or  15.2% (<25); 41.4% (25-29); 43.3%(>=30) | 8.8% (<18.5); 71.6% (18.5-24.99); 19.6% (>25) vs. 7.3% (<18.5); 76.3% (18.5-24.99); 16.4% (>25) or 7.6% (<18.5); 80.5% (18.5-24.99); 11.9% (>25) | 46.3% -53.7% (0) vs. 48.2-51.8% (0) or 35.2 - 64.8% (0) | NA | NA | NA | NA | Self-report | Sedentary activity-reading + tv; light activity- light level of garden > 3 hrs /wk -table tennis; moderate activity- sports or heavy leisure-time activities > 3 hrs/wk-running, swimming, playing tennis; heavy acitity (competitive sports several times/wk) |
| Blanque,2019(92) | Spain | 364 | 129 | March and April 2016 to the end of pregnancy | Wk14 | To the end of pregnancy | 30.6 ± 4.8 vs. 32.1 ±4.4 | 24±2.4 vs. 23.9±3 | NA | NA | 40±1.3 vs. 40±1.2 | NA | 165.1±5 vs. 164.6±6 | Objectively measured | Three 60-min sessions/wk (45 min of activity + 15 min of relaxation) |
| Dodd,2019(93) | Australia | 2602 | 629 | June 2014 to April 2017 | Wk16 | To the end of pregnancy | 31.5 ± 4.6 vs. 31.6 ±4.6 | 22.2±1.3 vs. 22.2±1.4 | 58.68% (0) vs. 59.8%(0) | NA | 39.5±1.6 vs. 39.1±2.4 | NA | 164.7±7.2 vs. 165.2±7.2 | Objectively measured | Physical activity |
| Kunath,2019(94) | Germany | 2461 | 2018 | NA | Wk8-9 | To the end of pregnancy | 30.4± 4.7 vs. 30.2 ±4.4 | 24.3±4.6 vs. 24.4±4.4 | 53 % (0) vs. 62% (0) | 68±13.7 vs. 68.4±13.1 | NA | NA | 167.2±6 vs. 167.4±6 | Objectively measured | Physical activity |
| Borgen,2019(95) | Norway | 125 | 68 | 2015-2018 | Wk1 | Whole pregnancy | 31.5± 3.5 vs. 33.1 ±3.9 | 22.4±1.7 vs. 21.6±1.7 | NA | NA | NA | NA | NA | Self-report | Physically active > 150 min/wk; |
| Huang,2019(96) | Australia | 329 | 57 | NA | Wk9.2 | To the end of pregnancy | 34.2± 1.6 vs. 32.5 ±1.6 | 25.3±1.8 vs. 26±2.1 | NA | NA | 38±1.1 vs. 38.4±0.5 | 11.8±2 vs. 13.3±1.8 | 164±3 vs. 166±2 | Self-report | Life-activity |
| Huang,2019(97) | China | 2155 | 2155 | September 2009 to March 2011 | Wk1 | Whole Pregnancy | 28± 4.3 vs. 28.3 ± 5 | 27.6 % (<18.5); 61.9% (18.5-23.9); 9%(>24) vs. 28.2 % (<18.5); 56.37% (18.5-23.9);9.5%(>24) | 75.5%(0) vs. 66.3%(0) | NA | 39±1.2 | NA | NA | Self-report | Physical activity |
| Schutt,2019(98) | US | 131 | 47 | April 2014 and March 2017 | Wk8-14 | To the end of pregnancy | 31.2±3.6 vs. 31.6 ± 4.6 | 24.3±4.1 vs. 25.7±5 | 1 | 67.3±15 vs. 71.7±15.3 | 39.5±1.7 vs. 39.1±2.1 | 9.5±1.4 vs. 9.7±1.6 | NA | Objectively measured | > 6 session of 15 to 30 min one on one visits with and RDN/Licensed Dietitian from no later than gestation wk 14 to childbirth. |
| Blanque,2020(99) | Spain | 386 | 129 | April 2016 to Feb. 2017 | Wk20 | 17 | 33.5±5.2 vs. 34.7 ± 4.4 | 24.9±4.8 vs. 24.7±4.1 | NA | 67.9±12.6 vs. 67.1±12.2 | NA | 11.2±3.5 vs. 8.3±2.8 | 165±5 vs. 165±6 | Objectively measured | SWEP program-physical exercise in an aquatic environement-3 times/wk-duration 60min (45 min of activity +15 min of relaxation) (warm up+ main phase(aerobic exercise + strength-endurance exercise)+ stretching and relaxation |

**Table S6 Quality assessment of selected studies using Newcastle-Ottawa scale**

|  | **Selection** | | | | **Comparability** | **Outcome** | | | **Total^3^** | **Scales^4^** |
| --- | --- | --- | --- | --- | --- | --- | --- | --- | --- | --- |
| **Score of each item of study**^1^ | **1** | **2** | **3** | **4** | **1** | **1** | **2** | **3** |  |  |
| Pomerance,1974(1) | 1 | 1 | 1 | 1 | 1 | 1 | 0 | 0 | 6 | Fair |
| Hall,1987(2) | 1 | 1 | 1 | 1 | 1 | 1 | 1 | 1 | 8 | Good |
| Briend,1980(3) | 1 | 1 | 0 | 1 | 0 | 1 | 1 | 1 | 6 | Fair |
| Dale,1982(4) | 1 | 1 | 1 | 1 | 1 | 1 | 1 | 1 | 8 | Good |
| Clapp,1984(5) | 1 | 1 | 1 | 1 | 1 | 1 | 1 | 0 | 7 | Good |
| Beckmann,1990(6) | 1 | 1 | 1 | 1 | 1 | 1 | 1 | 0 | 7 | Good |
| Clapp,1990(7) | 1 | 1 | 1 | 1 | 1 | 1 | 1 | 0 | 7 | Good |
| Klebanoff,1990(8) | 1 | 1 | 1 | 1 | 0 | 1 | 1 | 1 | 7 | Fair |
| Homer,1990(9) | 1 | 1 | 1 | 1 | 1 | 1 | 1 | 0 | 7 | Good |
| Botkin,1991(10) | 1 | 1 | 1 | 1 | 1 | 1 | 1 | 0 | 6 | Good |
| Rose,1991(11) | 1 | 1 | 0 | 1 | 1 | 1 | 1 | 1 | 7 | Good |
| Zeanah,1992(12) | 1 | 1 | 1 | 1 | 0 | 1 | 1 | 0 | 6 | Fair |
| Hatch,1993(13) | 1 | 1 | 1 | 1 | 1 | 1 | 1 | 0 | 7 | Good |
| Johnson,1994(14) | 1 | 1 | 0 | 1 | 1 | 1 | 1 | 1 | 7 | Good |
| Sternreld,1995(15) | 1 | 1 | 1 | 1 | 1 | 1 | 1 | 0 | 7 | Good |
| Henriksen,1995(16) | 1 | 1 | 0 | 1 | 1 | 1 | 1 | 0 | 6 | Good |
| Bell,1995(17) | 1 | 1 | 1 | 1 | 1 | 1 | 1 | 1 | 8 | Good |
| Clapp,2000(18) | 1 | 1 | 1 | 1 | 1 | 1 | 1 | 1 | 8 | Good |
| Bell,2002(19) | 1 | 1 | 0 | 1 | 0 | 1 | 1 | 1 | 6 | Fair |
| Leiferman,2003(20) | 1 | 1 | 0 | 1 | 1 | 1 | 1 | 0 | 6 | Fair |
| Takito,2005(21) | 1 | 1 | 0 | 1 | 1 | 1 | 1 | 0 | 6 | Good |
| Suezanne,2006(22) | 1 | 1 | 0 | 1 | 0 | 1 | 1 | 0 | 5 | Fair |
| Duncombe,2006(23) | 1 | 1 | 0 | 1 | 1 | 1 | 1 | 1 | 7 | Good |
| Dwarkanath,2007(24) | 1 | 1 | 0 | 1 | 1 | 1 | 1 | 0 | 6 | Good |
| Snapp,2008(25) | 1 | 1 | 0 | 1 | 0 | 1 | 1 | 0 | 5 | Fair |
| Mileur,2008(26) | 1 | 1 | 0 | 1 | 0 | 1 | 1 | 0 | 5 | Fair |
| Elden,2008(27) | 1 | 1 | 0 | 1 | 1 | 1 | 1 | 0 | 6 | Good |
| Owe,2009(28) | 1 | 1 | 0 | 1 | 1 | 1 | 1 | 1 | 7 | Good |
| Barakat,2009(29) | 1 | 1 | 1 | 1 | 1 | 1 | 1 | 0 | 7 | Good |
| Vrijkotte,2009(30) | 1 | 1 | 0 | 1 | 1 | 1 | 1 | 0 | 6 | Good |
| Hegaard,2010(31) | 1 | 1 | 1 | 1 | 1 | 1 | 1 | 0 | 7 | Good |
| Fleten,2010(32) | 1 | 1 | 0 | 1 | 0 | 1 | 1 | 0 | 5 | Fair |
| Hopkins,2010(33) | 1 | 1 | 1 | 1 | 1 | 1 | 1 | 0 | 7 | Good |
| Mottola,2010(34) | 1 | 1 | 0 | 1 | 1 | 1 | 1 | 1 | 7 | Good |
| Price,2012(35) | 1 | 1 | 1 | 1 | 1 | 1 | 1 | 0 | 7 | Good |
| Haakstad,2011(36) | 1 | 1 | 1 | 1 | 1 | 1 | 1 | 1 | 8 | Good |
| Jahromi,2011(37) | 1 | 1 | 0 | 1 | 1 | 1 | 1 | 1 | 7 | Good |
| Salonen,2011(38) | 1 | 1 | 1 | 1 | 0 | 1 | 1 | 1 | 7 | Fair |
| Nascimento,2011(39) | 1 | 1 | 1 | 1 | 1 | 1 | 1 | 1 | 8 | Good |
| Jukic,2012(40) | 1 | 1 | 0 | 1 | 1 | 1 | 1 | 1 | 7 | Good |
| Melo,2012(41) | 1 | 1 | 1 | 1 | 1 | 1 | 1 | 1 | 8 | Good |
| Mudd,2012(42) | 1 | 1 | 0 | 1 | 0 | 1 | 1 | 0 | 5 | Fair |
| Andersen,2012(43) | 1 | 1 | 1 | 1 | 1 | 1 | 1 | 0 | 7 | Good |
| Oostdam,2012(44) | 1 | 1 | 1 | 1 | 1 | 1 | 1 | 0 | 7 | Good |
| Krogsgaard,2013(45) | 1 | 1 | 0 | 1 | 1 | 1 | 1 | 0 | 6 | Good |
| Rauh,2013(46) | 1 | 1 | 1 | 1 | 1 | 1 | 1 | 1 | 8 | Good |
| Tomić,2013(47) | 1 | 1 | 1 | 1 | 1 | 1 | 1 | 1 | 8 | Good |
| Barakat,2014(48) | 1 | 1 | 1 | 1 | 1 | 1 | 1 | 0 | 7 | Good |
| Przybytowicz,2014(49) | 1 | 1 | 0 | 1 | 1 | 1 | 1 | 1 | 7 | Good |
| Kong, 2014(50) | 1 | 1 | 0 | 1 | 0 | 1 | 1 | 0 | 5 | Good |
| Reid,2014(51) | 1 | 1 | 0 | 1 | 1 | 1 | 1 | 0 | 6 | Good |
| Tanvig,2014(52) | 1 | 1 | 1 | 1 | 1 | 1 | 1 | 0 | 7 | Good |
| Li,2014(53) | 1 | 1 | 0 | 1 | 1 | 1 | 1 | 1 | 7 | Good |
| Ghods,2014(54) | 1 | 1 | 0 | 1 | 0 | 1 | 1 | 1 | 6 | Fair |
| Tanvig,2014 (55) | 1 | 1 | 1 | 1 | 1 | 1 | 1 | 0 | 7 | Good |
| Currie, 2014(56) | 1 | 1 | 1 | 1 | 1 | 1 | 1 | 1 | 8 | Good |
| Dodd,2014(57) | 1 | 1 | 0 | 1 | 1 | 1 | 1 | 0 | 6 | Good |
| Mudd,2015(58) | 1 | 1 | 0 | 1 | 0 | 1 | 1 | 0 | 5 | Fair |
| Vamos,2015(59) | 1 | 1 | 0 | 1 | 0 | 1 | 1 | 0 | 5 | Fair |
| Wang,2015(60) | 1 | 1 | 0 | 1 | 1 | 1 | 1 | 1 | 7 | Good |
| Dodd,2016(61) | 1 | 1 | 1 | 1 | 1 | 1 | 1 | 0 | 7 | Good |
| Ronnberg,2016(62) | 1 | 1 | 1 | 1 | 1 | 1 | 1 | 1 | 8 | Good |
| Lindqvist,2016(63) | 1 | 1 | 0 | 1 | 1 | 1 | 1 | 1 | 7 | Good |
| Daly,2016(64) | 1 | 1 | 0 | 1 | 1 | 1 | 1 | 1 | 7 | Good |
| Rego,2016(65) | 1 | 1 | 0 | 1 | 1 | 1 | 1 | 1 | 7 | Good |
| Silva,2017(66) | 1 | 1 | 1 | 1 | 1 | 1 | 1 | 1 | 8 | Good |
| Norris,2017(67) | 1 | 1 | 0 | 1 | 1 | 1 | 1 | 1 | 7 | Good |
| Díaz,2017(68) | 1 | 1 | 1 | 1 | 1 | 1 | 1 | 1 | 8 | Good |
| Huang,2017(69) | 1 | 1 | 0 | 1 | 1 | 1 | 1 | 1 | 7 | Good |
| Daly,2017(70) | 1 | 1 | 1 | 1 | 1 | 1 | 1 | 0 | 7 | Good |
| Wang,2017(71) | 1 | 1 | 1 | 1 | 1 | 1 | 1 | 0 | 7 | Good |
| Bacchi,2017(72) | 1 | 1 | 1 | 1 | 1 | 1 | 1 | 0 | 7 | Good |
| Garnas,2017(73) | 1 | 1 | 1 | 1 | 0 | 1 | 1 | 0 | 6 | Fair |
| Patel,2017(74) | 1 | 1 | 1 | 1 | 1 | 1 | 1 | 0 | 7 | Good |
| Barakat,2017(75) | 1 | 1 | 1 | 1 | 0 | 1 | 1 | 0 | 6 | Fair |
| Hegaard,2017(76) | 1 | 1 | 0 | 1 | 1 | 1 | 1 | 1 | 6 | Good |
| Badon,2017(77) | 1 | 1 | 0 | 1 | 1 | 1 | 1 | 0 | 6 | Good |
| Dhana,2017(78) | 1 | 1 | 0 | 1 | 1 | 1 | 1 | 0 | 6 | Good |
| McDonald,2018(79) | 1 | 1 | 1 | 1 | 1 | 1 | 1 | 0 | 7 | Good |
| Badon,2018(80) | 1 | 1 | 0 | 1 | 1 | 1 | 1 | 1 | 7 | Good |
| Barakat,2018(81) | 1 | 1 | 1 | 1 | 1 | 1 | 1 | 0 | 7 | Good |
| Chan,2018(82) | 1 | 1 | 1 | 1 | 1 | 1 | 1 | 0 | 7 | Good |
| Mizgier,2018(83) | 1 | 1 | 1 | 1 | 1 | 1 | 1 | 1 | 8 | Good |
| Myrex,2018(84) | 1 | 1 | 0 | 1 | 1 | 1 | 1 | 1 | 7 | Good |
| Poppel,2019(85) | 1 | 1 | 1 | 1 | 1 | 1 | 1 | 0 | 7 | Good |
| Hoffmann,2019(86) | 1 | 1 | 0 | 1 | 1 | 1 | 1 | 0 | 6 | Good |
| Brik,2019(87) | 1 | 1 | 1 | 1 | 1 | 1 | 1 | 0 | 7 | Good |
| Mcmillan,2019(88) | 1 | 1 | 1 | 1 | 1 | 1 | 1 | 0 | 7 | Good |
| Barakat,2019(89) | 1 | 1 | 1 | 1 | 1 | 1 | 1 | 0 | 7 | Good |
| Clark,2019(90) | 1 | 1 | 1 | 1 | 1 | 1 | 1 | 0 | 7 | Good |
| Jochumsen,2019(91) | 1 | 1 | 0 | 1 | 1 | 1 | 1 | 1 | 7 | Good |
| Blanque,2019(92) | 1 | 1 | 1 | 1 | 1 | 1 | 1 | 0 | 7 | Good |
| Dodd,2019(93) | 1 | 1 | 1 | 1 | 1 | 1 | 1 | 0 | 7 | Good |
| Kunath,2019(94) | 1 | 1 | 1 | 1 | 1 | 1 | 1 | 1 | 8 | Good |
| Borgen,2019(95) | 1 | 1 | 0 | 1 | 1 | 1 | 1 | 0 | 6 | Good |
| Huang,2019(96) | 1 | 1 | 0 | 1 | 1 | 1 | 1 | 0 | 6 | Good |
| Huang,2019(97) | 1 | 1 | 0 | 1 | 1 | 1 | 1 | 1 | 7 | Good |
| Schutt,2019(98) | 1 | 1 | 1 | 1 | 1 | 1 | 1 | 0 | 7 | Good |
| Blanque,2020(99) | 1 | 1 | 1 | 1 | 1 | 1 | 1 | 0 | 7 | Good |

**Footnotes**

^1^Characteritic of included studies were presented in Table S4.

^2^Minimum number of possible stars to be awarded = 0, maximum number of possible stars to be awarded = 9.

^3^Poor = 0-2 stars, Fair = 5-7 stars, Good = 6-8 stars.

**Table S7 Sensitivity analysis of outcomes using trim and fill method in DerSimonian and Laird random model**

| **Item** | **Trim and Fill** | | | |  | **Funnel plot asymmetry** | |
| --- | --- | --- | --- | --- | --- | --- | --- |
|  | SMD (SE) ^1^ | 95% CI ^2^ | *I^2^* (%) | *P* | N | t | *P* |
| ***Birth weight*** |  |  |  |  |  |  |  |
| MNW^3^ |  |  |  |  |  |  |  |
| Exercise only^4^ | -0.33(0.06) | -0.45 -0.21 | 99.1 | <0.001 | 79 | -1.3 | 0.21 |
| Exercise only^5^ | -0.36 (0.07) | -0.51 -0.21 | 99.2 | <0.001 | 66 | -1.1 | 0.29 |
| Exercise + confounders^4^ | 0.19(0.10) | -0.01 0.39 | 88.3 | 0.07 | 9 | 2.1 | 0.07 |
| Exercise + confounders^5^ | 0.45(0.19) | -0.09 0.82 | 81.3 | 0.01 | 5 | -0.23 | 0.83 |
| MO^3^ |  |  |  |  |  |  |  |
| Exercise only | -0.15(0.09) | -0.33 0.02 | 81.7 | 0.09 | 12 | -0.95 | 0.36 |
| Exercise + confounders | 0.04(0.07) | -0.11 0.18 | 72.11 | 0.62 | 6 | -0.24 | 0.83 |
| ***Preterm odds ratio*** |  |  |  |  |  |  |  |
| MNW |  |  |  |  |  |  |  |
| Exercise only^4^ | -0.24(0.08) | -0.40 -0.07 | 83.0 | 0.005 | 31 | -1.55 | 0.13 |
| Exercise only^5^ | -0.28(0.10) | -0.47 -0.08 | 81.9 | 0.006 | 24 | -0.89 | 0.39 |
| Exercise + confounders | -0.47(0.23) | -0.93 -0.01 | 0.0 | 0.5 | 5 | 0.32 | 0.77 |
| ***SGA odds ratio*** |  |  |  |  |  |  |  |
| MNW |  |  |  |  |  |  |  |
| Exercise only^4^ | -0.18(0.08) | -0.34 -0.02 | 74.5 | 0.02 | 33 | 0.74 | 0.46 |
| Exercise only^5^ | -0.20(0.09) | -0.37 -0.03 | 75.7 | 0.02 | 31 | 0.54 | 0.59 |
| Exercise + confounders^4^ | 0.04(0.12) | -0.19 0.27 | 0.0 | 0.74 | 8 | -0.04 | 0.97 |
| Exercise + confounders^5^ | -0.20(0.47) | -1.12 0.71 | 0.0 | 0.67 | 3 | -14.3 | 0.045 |
| MO |  |  |  |  |  |  |  |
| Exercise only | -0.32(0.19) | -0.69 0.05 | 40.4 | 0.09 | 8 | 0.59 | 0.58 |
| Exercise + confounders | -0.04(0.31) | -0.65 0.57 | 3.2 | 0.90 | 8 | -0.33 | 0.75 |
| ***LGA odds ratio*** |  |  |  |  |  |  |  |
| MNW |  |  |  |  |  |  |  |
| Exercise only^4^ | -0.13(0.06) | -0.25 0.0008 | 60.5 | 0.05 | 36 | 0.84 | 0.41 |
| Exercise only^5^ | -0.06(0.07) | -0.19 0.07 | 62.5 | 0.40 | 28 | 1.34 | 0.19 |
| Exercise + confounders^4^ | 0.002(0.12) | -0.23 0.23 | 0.0 | 0.98 | 7 | 0.33 | 0.75 |
| Exercise + confounders^5^ | 0.17(0.21) | -0.24 0.59 | 0.0 | 0.41 | 5 | -0.42 | 0.70 |
| MO |  |  |  |  |  |  |  |
| Exercise only | -1.06(0.33) | -1.70 -0.41 | 97.7 | 0.001 | 12 | 1.94 | 0.08 |
| Exercise + confounders | 0.37(0.22) | -0.06 0.81 | 0.0 | 0.09 | 6 | 0.23 | 0.83 |
| ***Infant and child body weight***  MNW |  |  |  |  |  |  |  |
| Infant (< 2 years) | -0.004(0.23) | -0.47 0.46 | 72.1 | 0.99 | 5 | -0.46 | 0.68 |
| Child (2-15 years)^4^ | 0.01(0.09) | -0.16 0.18 | 88.9 | 0.88 | 7 | 3.00 | 0.03 |
| Child (2-15 years) ^5^ | 0.15(0.11) | -0.06 0.36 | 85.2 | 0.16 | 7 | 2.30 | 0.07 |
| ***Child obesity odds ratio***  MNW |  |  |  |  |  |  |  |
| Exercise only (2-15 years) | -0.75(0.14) | -1.02 -0.47 | 77.0 | 0.001 | 3 | -1.45 | 0.38 |

**Footnote**

^1^SMD (SE), standard mean difference.

^2^CI, 95% confidence interval.

^3^MNW (maternal normal weight; pre-pregnancy BMI 18.5-24.9 kg/m^2^) and MO (maternal overweight & obesity; pre-pregnancy BMI ≥ 25 kg/m^2^).

^4^Sensitivity analysis included studies with minor portion of diabetic and hypertensive participates during pregnancy.

^5^Sensitivity analysis excluded studies with minor portion of diabetic and hypertensive participates during pregnancy.

**Electronic Supplementary Material Appendix Figures**

**Fig. S1 Study bias assessed by funnel plots**

**(1)** Funnel plot: effects of maternal exercise in pre-pregnancy normal weight (MNW) and overweight or obesity (MO) on birth weight (SMD, standard mean difference).

| 1.MNW   - 1. Exercise only   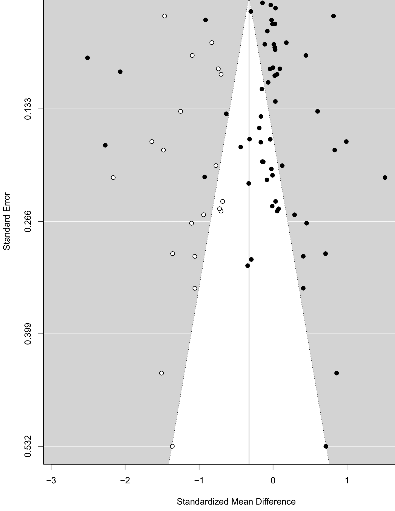  Asymmetry: t =-1.3, P = 0.21 | - 1. Exercise + confounders   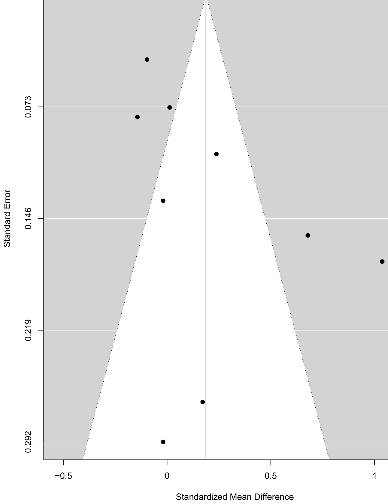  Asymmetry: t = 2.1, P =0.07 | 2.MO  2.1 Exercise only  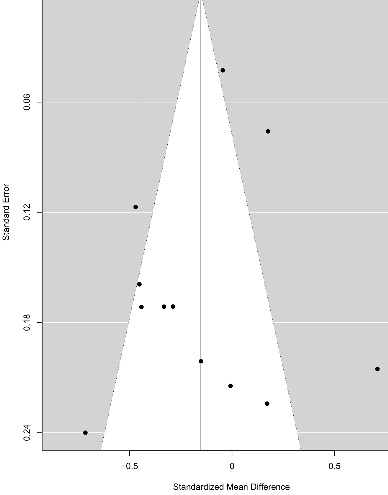Asymmetry: t =-0.95, P = 0.36 | 2.2 Exercise + confounders  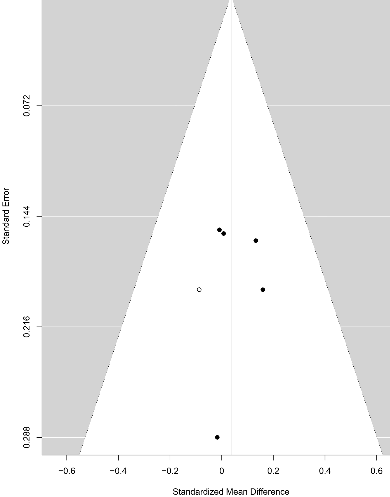  Asymmetry: t = -0.24, P = 0.83 |
| --- | --- | --- | --- |

**(2)** Funnel plot: effects of maternal exercise during pregnancy in pre-pregnancy normal weight (MNW) on odds ratio (OR) of preterm birth

| 1.MNW  1.1 Exercise only  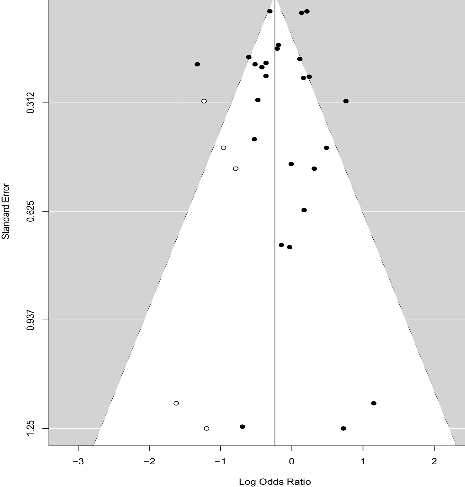  Asymmetry: t =-1.55, P = 0.13 | 1.2 Exercise + confounders  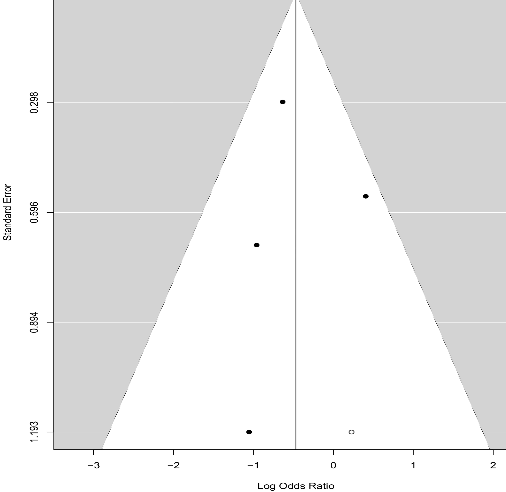  Asymmetry: t = 0.32, P = 0.77 |
| --- | --- |

**(3)** Funnel plot: effects of maternal exercise during pregnancy in women with pre-pregnancy normal weight (MNW) and overweight and obesity (MO) on odds ratio (OR) of small for gestational age (SGA).

| 1.MNW  1.1 Exercise only  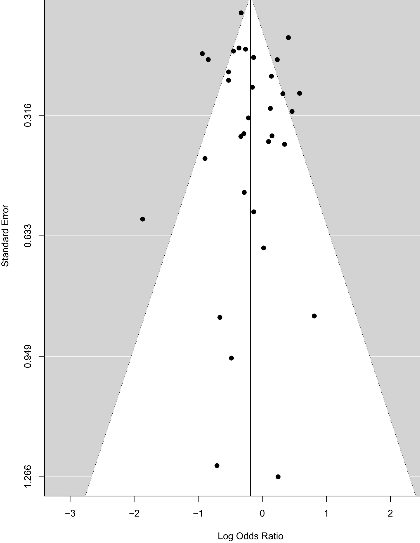  Asymmetry: t = 0.74, P = 0.46 | 1.2Exercise + confounders  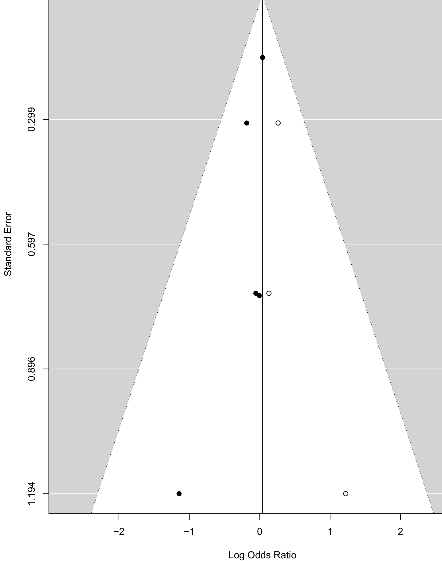  Asymmetry: t = -0.04, P = 0.97 | 2.MO  2.1 Exercise only  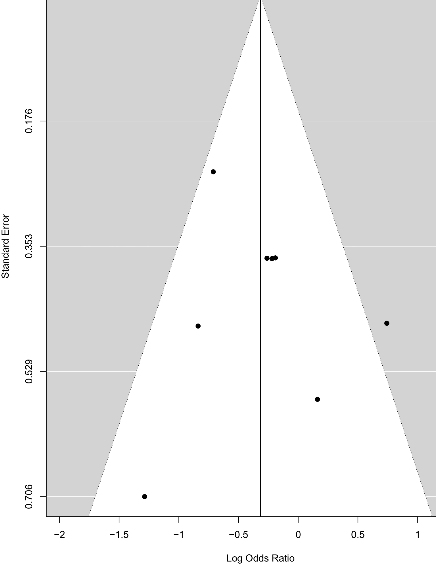  Asymmetry: t = 0.59, P = 0.58 | 2.2 Exercise + confounders  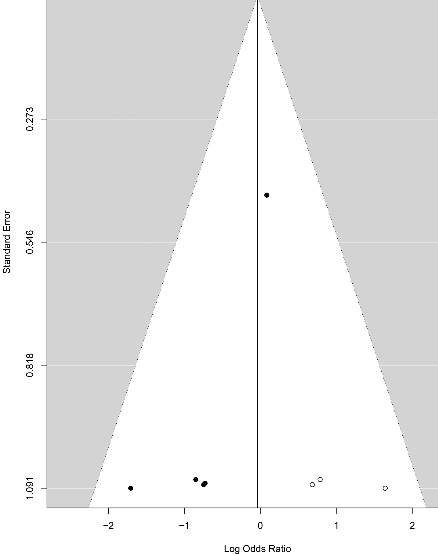  Asymmetry: t = -0.33, P = 0.75 |
| --- | --- | --- | --- |

**(4)** Funnel plot: effects of maternal exercise during pregnancy in women with pre-pregnancy normal body weight (MNW) and overweight and obesity (MO) on odds ratio (OR) of large for gestational age (LGA).

| 1.MNW  1.1 Exercise only  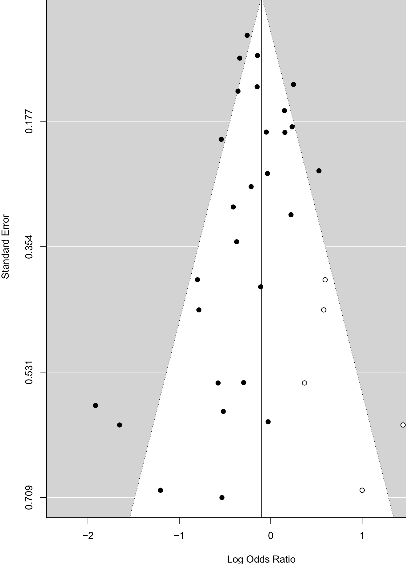  Asymmetry: t = 0.84, P = 0.41 | 1.2 Exercise + confounders  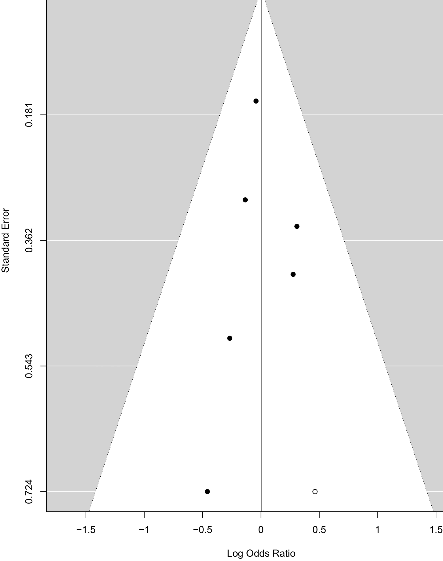  Asymmetry: t = 0.33, P = 0.75 | 2.MO  2.1 Exercise only  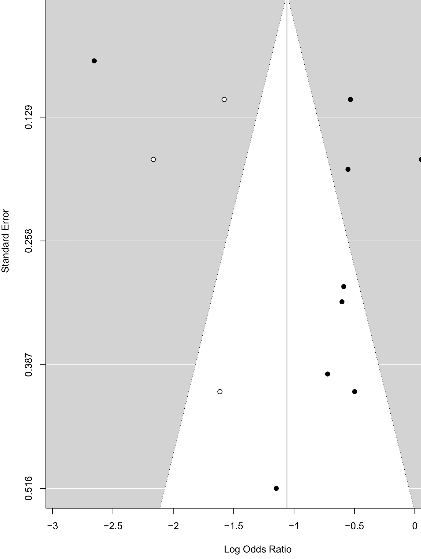  Asymmetry: t = 1.94, P = 0.08 | 2.2 Exercise + confounders  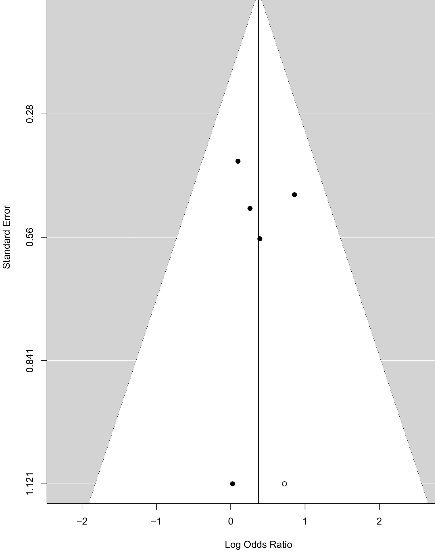  Asymmetry: t = 0.23, P = 0.83 |
| --- | --- | --- | --- |

**(5)** Funnel plot: effects of maternal exercise during pregnancy in women with pre-pregnancy normal (MNW) on infant and child body weight (SMD, standard mean difference).

| 1.1 Exercise only - Infant body mass  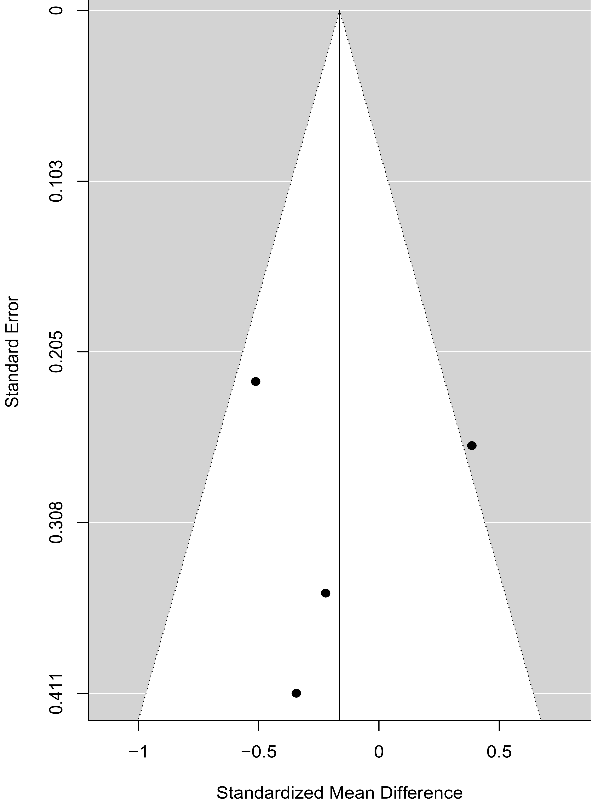  Asymmetry: t = -0.46, P = 0.68 | 1.2 Exercise only-Child body mass  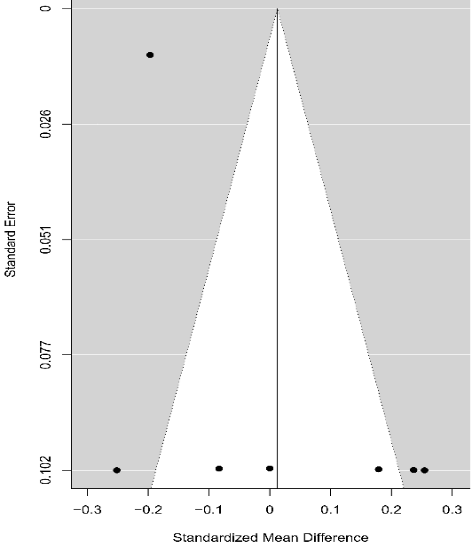  Asymmetry: t = 2.98, P = 0.03 |
| --- | --- |

**(6)** Funnel plot: effects of maternal exercise during pregnancy in women with pre-pregnancy normal weight (MNW) on odds ratio (OR) of child obesity


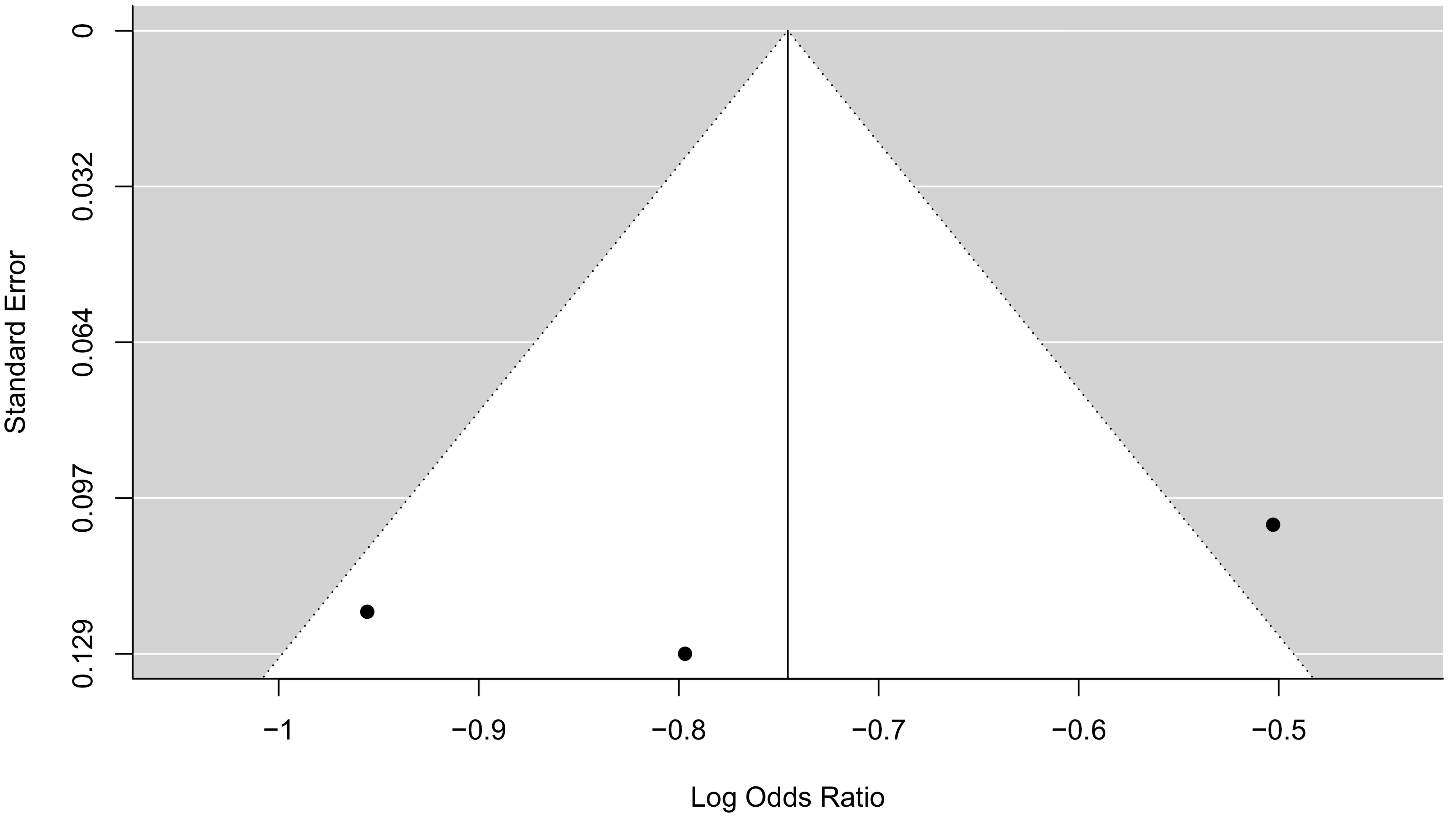
Asymmetry: t=-1.45, P = 0.38

**Fig. S2 Forest plots displaying effects of exercise-only interventions and exercise plus confounders of pregnant women with pre-pregnancy normal weight (MNW; BMI 18.5-24.9 kg/m^2^) and overweight and obesity (MO; BMI ≥ 25 kg/m^2^) on birth weight. SMD, standard mean difference. Error bars indicate 95% confidence interval. MET means metabolic equivalent.**


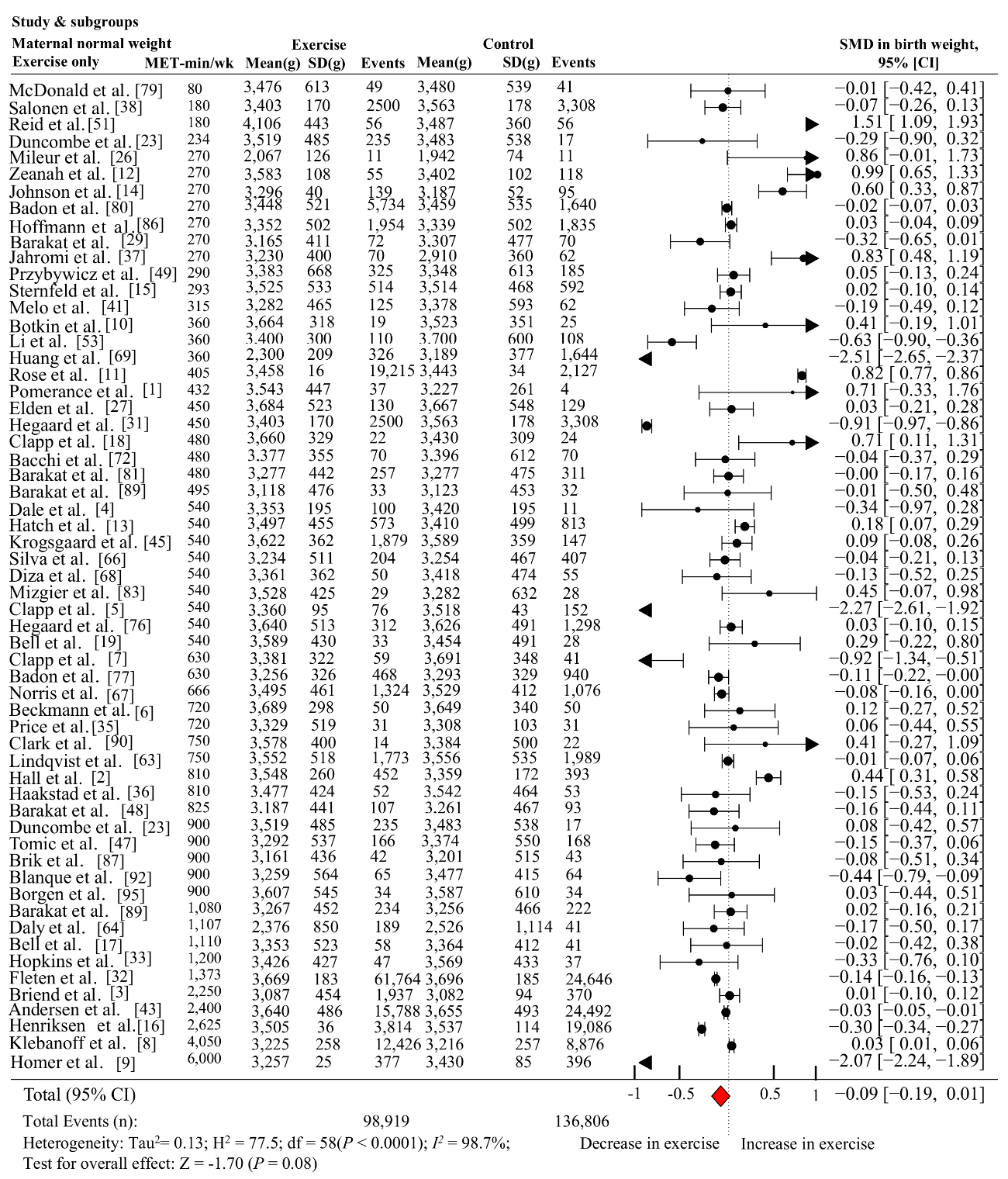


Continue Fig. S2


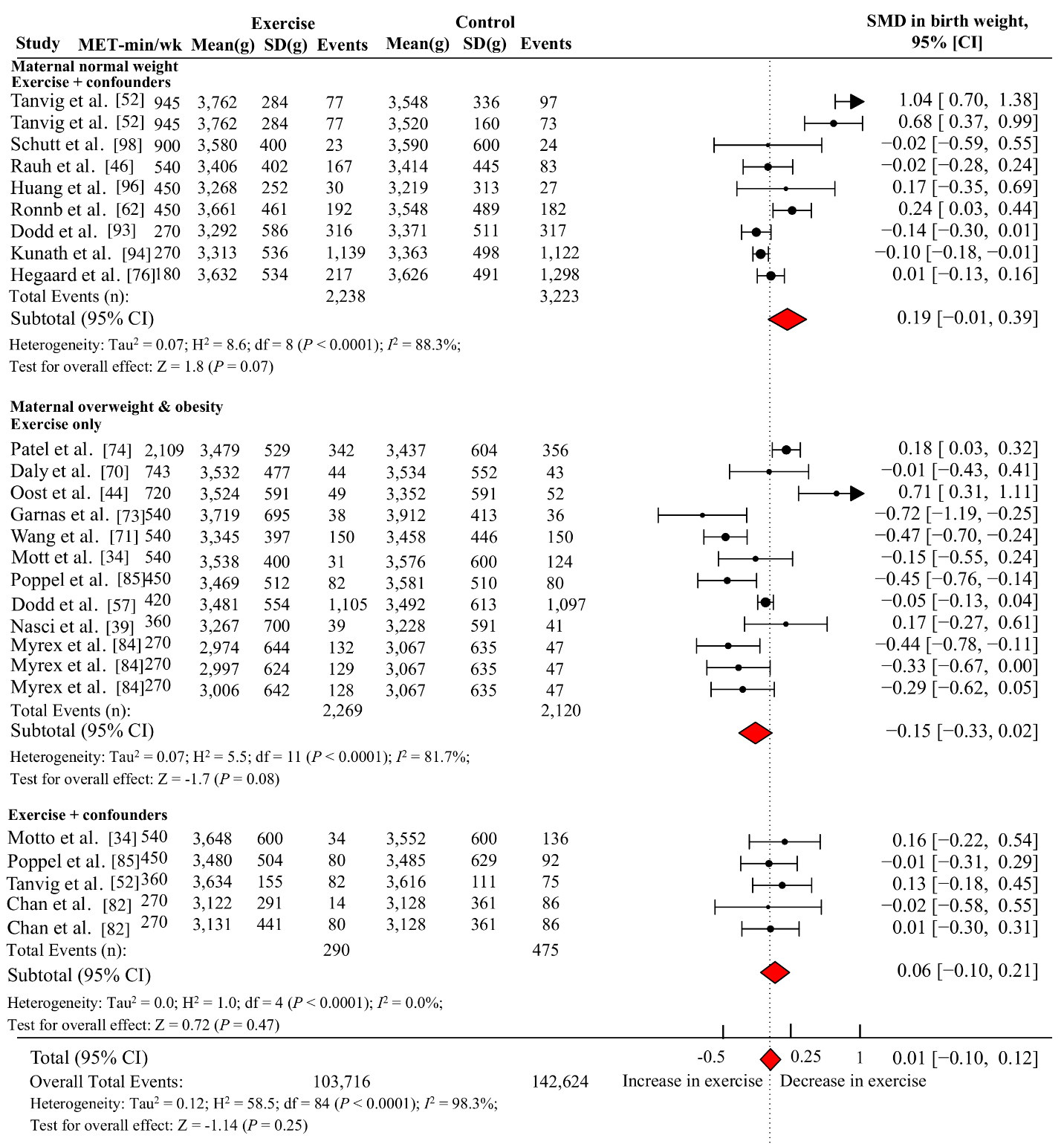


**Fig. S3 Forest plot displaying effects of exercise interventions in pregnant women with pre-pregnancy normal weight (MNW; BMI 18.5-24.9 kg/m^2^) on infant (≤ 2 years) and child (2-15 years) body mass. SMD, standard mean difference. Error bars indicate 95% confidence internal. MET means metabolic equivalent.**

**
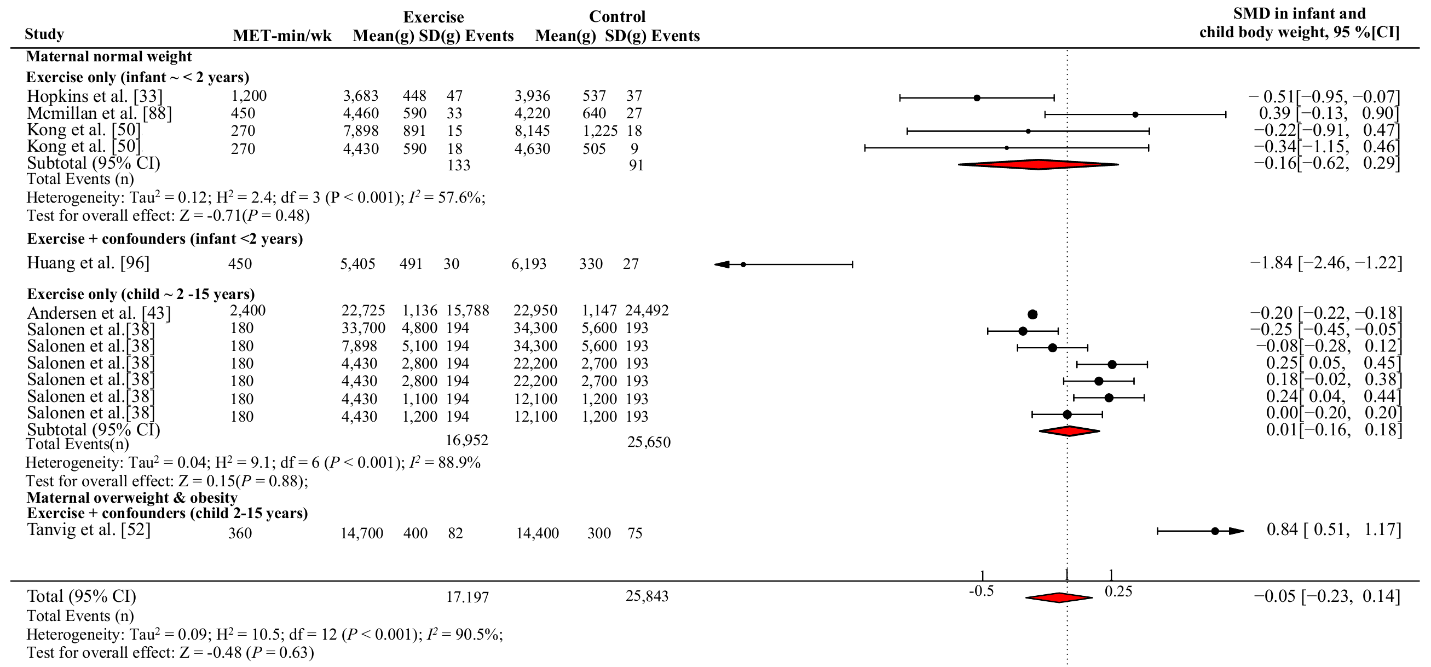
**

**Fig. S4 Forest plot displaying effects of exercise in pregnant women with pre-pregnancy normal body weight (MNW; BMI 18.5-24.9 kg/m2) on odds ratio (OR) of child obesity (2-15 years; BMI ≥ 95th percentile). Error bars indicate 95% CI. MET means metabolic equivalent.**

**
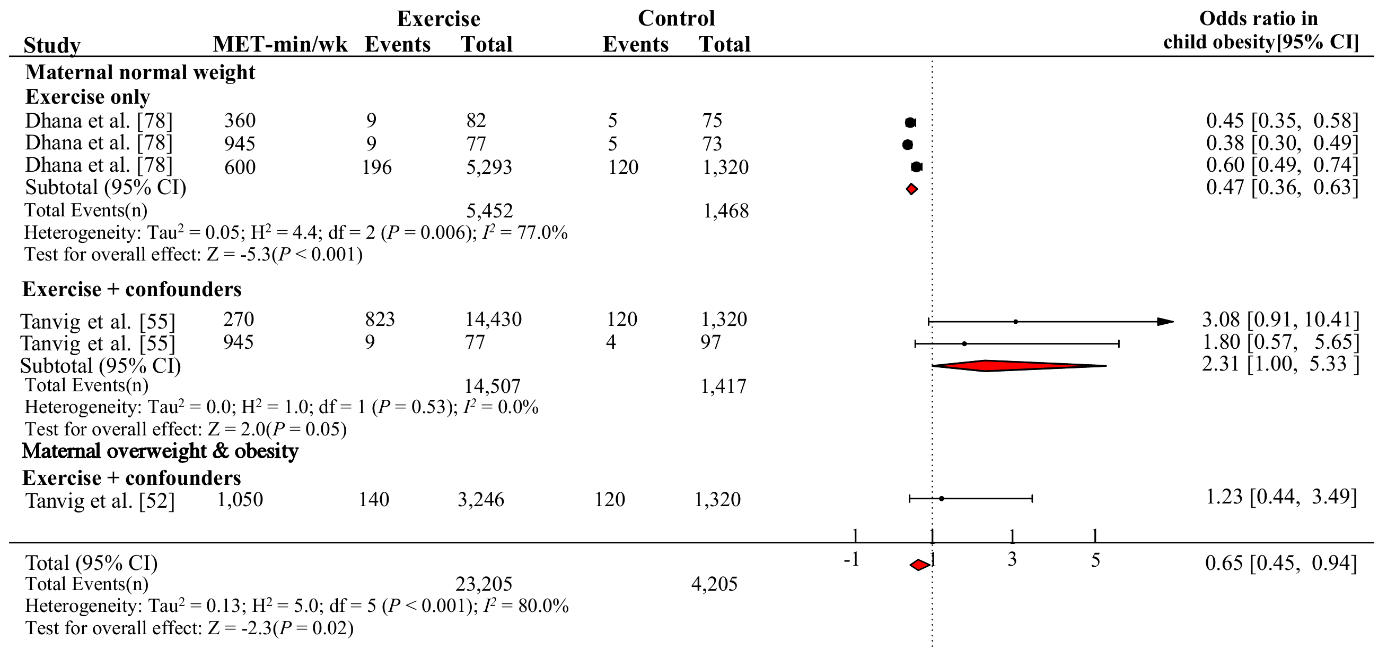
**

**Fig. S5 Association of volume of exercise-only interventions (A) or exercise plus confounders (B) with birth weight in maternal normal weight (MNW) mothers (pre-pregnancy body mass index 18.5-24.9 kg/m^2^). SMD standard mean difference, MET metabolic equivalent.**

**
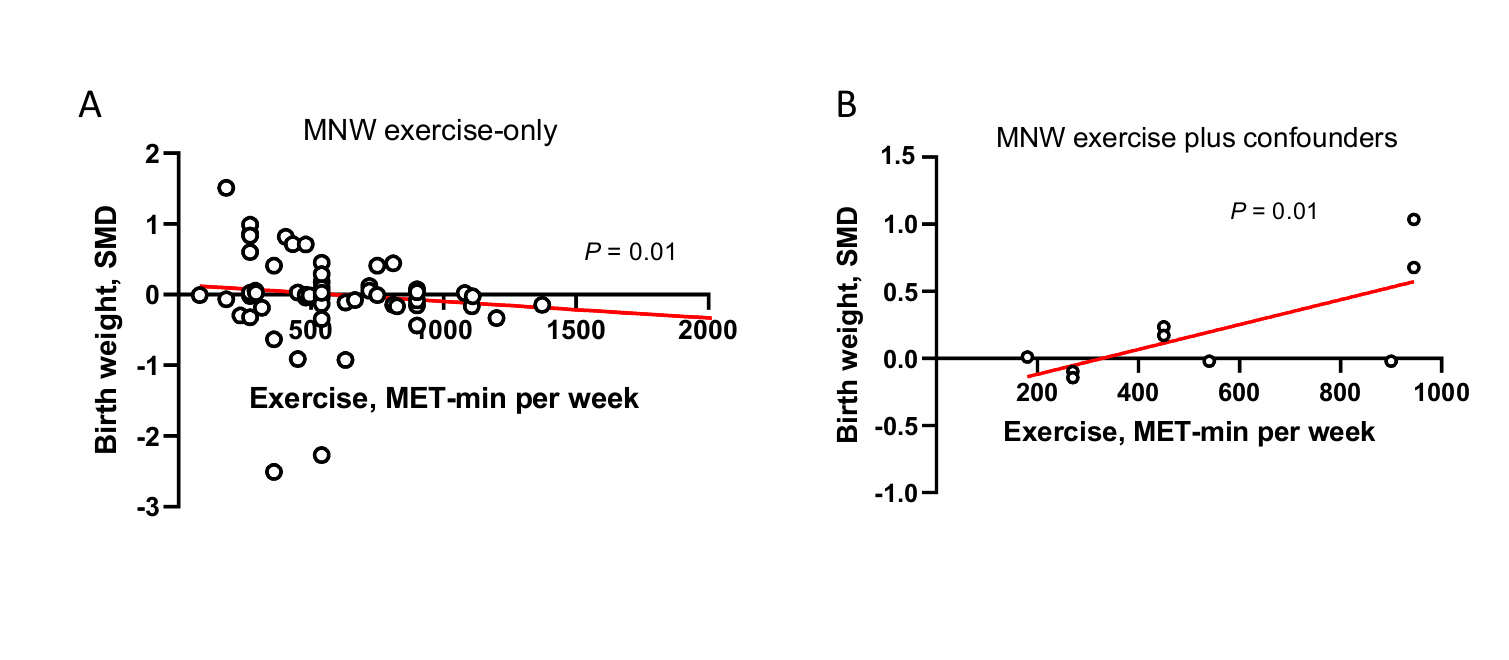
**

**References**

1. Pomerance JJ, M.D., Gluck MPHL, Lynch MDVA, M.D. Physical fitness in pregnancy: Its effect on pregnancy outcome. Am J Obstet Gynecol. 1974;119(7):867-876.

2. Hall DC, M.D., Kaufmann DA. Effects of aerobic and strength conditioning on pregnancy outcomes. Am J Obstet Gynecol. 1987;157(5):1199-1203.

3. Briend A. Maternal physical activity, birth weight and perinatal mortality. Med Hypotheses. 1980;6(11):1157-1170. https://www.sciencedirect.com/science/article/abs/pii/0306987780901383?via%3Dihub

4. Dale E, Mullinax KM, Bryan DH. Exercise during pregnancy: Effects on the Fetus. Can J Appl Sport Sci. 1982;7(2):98-103.

5. Clapp JF, Dickstein S. Endurance exercise and pregnancy outcome. Med Sci Sport Exerc. 1984;16(6):556-562.

6. Beckmann CRB, M.D., Beckmann MHPECA. Effect of a structured antepartum exercise program on pregnancy and labor outcome in primiparas. J Reprod Med. 1990;35(7):704-708.

7. Clapp JF, M.D., Capeless EL. Neonatal morphometrics after endurance exercise during pregnancy. Am J Obstet Gynecol. 1990;163(6):1805-1811.

8. Klebanoff MA, Shiono PH, Carey JC. The effect of physical activity during pregnancy on preterm delivery and birth weight. Am J Obstet Gynecol. 1990;163(5):1450-1456.

9. Homer CJ, Beresfordt SAA, Jarnest SA, Siege E, Wilcox S. Work-related physical exertion and risk of preterm, low birthweight delivery. Paediatr Perinat Epidemiol. 1990;4(2):161-174.

10. Botkin C, Driscoll CE. Maternal aerobic exercise: Newborn effects. Fam Pract Res J. 1991;11(4):387-393.

11. Rose NC, Haddow JE, Palomaki GE, Knight GJ. Self-Related physical activity level during the second trimester and pregnancy outcome. Obstet Gynecol. 1991;78(6):1078-1080.

12. Zeanah M, Schlosser SP. Adherence to ACOG guidelines on exercise during pregnancy : Effect on pregnancy outcome. Clin Stud. 1993;22(4):329-335.

13. Hatch MC, McLean DE, Levin B, Begg M, Reuss L, Susser M. Maternal exercise during pregnancy, physical fitness and fetal growth. Am J Epidemiol. 1993;137(10):1105-1114.

14. Johnson AA, Knight EM, Edwards CH, et al. Selected lifestyle practives in Urban African American women-Relationships to pregnancy outcome, dietary intakes and anthropometric measurements. J Nutr. 1994;124(6):S963-S972.

15. Sternreld B, Quesenberry CP, JR, Eskenazi B, Newman LA. Exercise during pregnancy and pregnancy outcome. Med Sci Sport Exerc. 1995;27(5):634-640.

16. Henriksen TB, Hedegaard M, Secher NJ. Standing and walking at work and birthweight. Acta Obs Gynecol ogica Scand. 1995;74(7):509-516.

17. Bell RJt effect of vigorous exercise during pregnancy on birth-weight, Palma SM, Lumley JM. The effect of vigorous exercise during pregnancy on birth-weight. Obstet Gynaecol. 1995;35(1):46-51.

18. Iii JFC, Kim H, Burciu B, Lopez B. Beginning regular exercise in early pregnancy : Effect on fetoplacental growth. Am J Obstet Gynecol. 2000;183(6):1484-1488. doi:10.1067/mob.2000.107096

19. Bell R. The Effects of Vigorous Exercise During Pregnancy on Birth Weight. J Sci Med Sport. 2002;5(1):32-36.

20. Leiferman JA, Evenson KR. The Effect of Regular Leisure Physical Activity on Birth Outcomes. Matern Child Health J. 2003;7(1):59-64.

21. Takito MY, Helena M, Benício DA, Dias R, Paulo S. Maternal posture and its influence on birthweight. Matern Phys Act birthweight. 2005;39(3):1-7.

22. Orr ST, James SA, Garry J, Prince CB, Newton ER. Exercise and pregnancy outcome among urban, low-income, black women. Autumn. 2006;16(4):933-937.

23. Duncombe D, Skouteris H, Wertheim EH, Kelly L, Fraser V, Paxton SJ. Vigorous exercise and birth outcomes in a sample of recreational exercisers : A prospective study across pregnancy. Aust New Zeal J Obstet Gynaecol. 2006;46(March):288-292. doi:10.1111/j.1479-828X.2006.00594.x

24. Dwarkanath P, Muthayya S, Vaz M. The relationship between maternal physical activity during pregnancy and birth weight. Asia Pac J Clin Nutr. 2007;16(4):704-710.

25. Snapp CA, Donaldson SK. Gestational Diabetes Mellitus: Physical Exercise and Health Outcomes. Biol Reseach Nurs. 2008;10(2):145-155.

26. Moyer-mileur LJ, Ball SD, Brunstetter VL, Chan GM. Maternal-administered physical activity enhances bone mineral acquisition in premature very low birth weight infants. J Perinatol. 2008;28(March):432-437. doi:10.1038/jp.2008.17

27. Elden H, Ostgaard H, Fagevik-olsen M, Ladfors L, Hagberg H. Treatments of pelvic girdle pain in pregnant women : adverse effects of standard treatment , acupuncture and stabilising exercises on the pregnancy , mother , delivery and the fetus/ neonate. BMC Complement Altern Med. 2008;8(34):1-13. doi:10.1186/1472-6882-8-34

28. Owe KM, Nystad W, Bø K. Association Between Regular Exercise and Excessive Newborn Birth Weight. Obstet Gynecol. 2009;114(4):770-776.

29. Barakat R, Lucia A, Ruiz JR. Resistance exercise training during pregnancy and newborn’s birth size: a randomised controlled trial. Int J Obes. 2009;33:1048-1057. doi:10.1038/ijo.2009.150

30. Vrijkotte TGM, Wal MF Van Der, Eijsden M Van, Bonsel GJ. First-Trimester Working Conditions and Birthweight: A Prospective Cohort Study. Res Pract. 2009;99(8):1409-1416. doi:10.2105/AJPH.2008.138412

31. Hegaard HK, Petersson K, Hedegaard M, et al. Sports and leisure-time physical activity in pregnancy and birth weight : a population-based study. Scand J Med Sci Sport. 2010;20:96-102. doi:10.1111/j.1600-0838.2009.00918.x

32. Fleten C, Stigum H, Magnus P, Nystad W. Exercise During Pregnancy , Maternal Prepregnancy Body Mass Index, and Birth Weight. Obstet &Gynecology. 2010;115(2):331-337.

33. Hopkins SA, Baldi JC, Cutfield WS, Mccowan L, Hofman PL. Exercise training in pregnancy reduces offspring size without changes in maternal insulin sensitivity. Endocr Care. 2010;95(May):2080-2088. doi:10.1210/jc.2009-2255

34. Mottola MF, Giroux I, Gratton R, et al. Nutrition and Exercise Prevent Excess Weight Gain in Overweight Pregnant Women. Med Sci Sport Exerc. 2010;42(2):265-272. doi:10.1249/MSS.0b013e3181b5419a.Nutrition

35. Price BB, Amini SB, Kappeler K. Exercise in Pregnancy: Effect on Fitness and Obstetric Outcomes—A Randomized Trial. Med Sci Sport Exerc. 2012;44(12):2263-2269. doi:10.1249/MSS.0b013e318267ad67

36. Haakstad LAH, Bø K. Exercise in pregnant women and birth weight : a randomized controlled trial. BMC pregnancy Childbirth. 2011;11:66-72.

37. Jahromi MK, Jahromi BN, Hojjati S. Relationship between Daily Physical Activity During Last Month of Pregnancy and Pregnancy Outcome. Iran Red Crescent Med J. 2011;13(1):15-20.

38. Salonen MK, Kajantie E, Osmond C, et al. Developmental Origins of Physical Fitness : The Helsinki Birth Cohort Study. PLoS One. 2011;6(7):1-7. doi:10.1371/journal.pone.0022302

39. SL N, FG S, MÂ P, S S, JL P e S. The effect of an antenatal physical exercise programme on maternal / perinatal outcomes and quality of life in overweight and obese pregnant women : a randomised clinical trial. Gen Obstet. 2011;118:1455-1463. doi:10.1111/j.1471-0528.2011.03084.x

40. Jukic AMZ, Evenson KR, Daniels JL, Amy H. Herring, Wilcox AJ, Hartmann KE. A Prospective Study of the Association Between Vigorous Physical Activity During Pregnancy and Length of Gestation and Birthweight. Matern Child Heal J. 2012;16:1031-1044. doi:10.1007/s10995-011-0831-8

41. Melo AS de O, JL S, JS T, VO B, DF L, MM A. Effect of a Physical Exercise Program During Pregnancy on Uteroplacental and Fetal Blood Flow and Fetal Growth. Obstet Gynecol. 2012;120(2):302-310. doi:10.1097/AOG.0b013e31825de592

42. Mudd LM, Pivarnik J, Holzman CB, Paneth N, Pfeiffer K, Chung H. Leisure-time physical activity in pregnancy and the birth weight distribution: Where is the effect? J Phys Act Health. 2012;9(8):1168-1177.

43. Andersen CS, Juhl M, Gamborg M, Sørensen TIA, Nohr EA. Maternal Recreational Exercise during Pregnancy in relation to Children ’ s BMI at 7 Years of Age. Int J Pediatr. 2012;2012:1-8. doi:10.1155/2012/920583

44. Oostdam N, Poppel MNM Van, Wouters M, Eekhoff EMW, Bekedam DJ. No effect of the FitFor2 exercise programme on blood glucose , insulin sensitivity , and birthweight in pregnant women who were overweight and at risk for gestational diabetes : results of a randomised controlled trial. Int J Obstet Gynaecol. 2012;119(9):1098-1107. doi:10.1111/j.1471-0528.2012.03366.x

45. Krogsgaard S, Gudmundsdottir SL, Nilsen TIL. Prepregnancy Physical Activity in relation to Offspring Birth Weight : A Prospective Population-Based Study in Norway — The HUNT Study. J Pregnancy. 2013;2013:1-7.

46. Rauh K, Gabriel E, Kerschbaum E, et al. Safety and efficacy of a lifestyle intervention for pregnant women to prevent excessive maternal weight gain : a cluster-randomized controlled trial. BMC Pregnancy Childbirth. 2013;13(151):1-11.

47. Tomić V, G S, J T, Z M, D Z-K, S P. The effect of maternal exercise during pregnancy on abnormal fetal growth. Croat Med J. 2013;54(4):362-369. doi:10.3325/cmj.2013.54.362

48. Barakat R, Perales M, Bacchi M, Coteron J, Refoyo I. A Program of Exercise Throughout Pregnancy. Is It Safe to Mother and Newborn? Sci Lifestyle Chang. 2014;29(1):2-9. doi:10.4278/ajhp.130131-QUAN-56

49. Przybyłowicz K, Przybyłowicz M, Grzybiak M, Janiszewska K. Effects of physical activity during pregnancy and gestational weight gain on the nutritional status of newborns in warminsko-mazurskie voivodeship. Acta Sci Pol, Technol Aliment. 2014;13(2):203-211.

50. Kong KL, Campbell C, Wagner K, Peterson A. Impact of a walking intervention during pregnancy on post-partum weight retention and infant anthropometric outcomes. J Dev Orig Health Dis. 2014;5(3):259-267. doi:10.1017/S2040174414000117

51. Reid EW, Registered RM, Mcneill JA, et al. Physical activity , sedentary behaviour and fetal macrosomia in uncomplicated pregnancies : A prospective cohort study. Midwifery. 2014;30(12):1202-1209. doi:10.1016/j.midw.2014.04.010

52. Tanvig M, Vinter CA, Jørgensen JS, et al. Anthropometrics and Body Composition by Dual Energy X-Ray in Children of Obese Women : A Follow-Up of a Randomized Controlled Trial (the Lifestyle in Pregnancy and Offspring [LiPO] Study). PLoS One. 2014;9(2):1-8. doi:10.1371/journal.pone.0089590

53. Qiuling L, Hong C, Dongming Z, Na L, Liang C, Caixia L. Effects of walking exercise during late trimester on pregnancy outcome of low-risk primipara. Natl Med J China. 2014;94(22):1722-1725.

54. Ghodsi Z, Asltoghiri M. Effects of aerobic exercise training on maternal - Journal of Pakistan Medical Association (Pakistan) - September 30 , 2014. J Pakistan Med Assoc. 2014;64(9):1-7.

55. Tanvig M, Vinter CA, Jørgensen JS, et al. Effects of Lifestyle Intervention in Pregnancy and Anthropometrics at Birth on Offspring Metabolic Profile at 2.8 Years : Results From the Lifestyle in Pregnancy and Offspring (LiPO) Study. J Clin Endocrinol Metab. 2015;100(January):175-183. doi:10.1210/jc.2014-2675

56. Currie LM, Woolcott CG, Fell DB, Armson BA, Dodds L. The Association Between Physical Activity and Maternal and Neonatal Outcomes : A Prospective Cohort. Matern Child Heal J. 2014;18(8):1823-1830. doi:10.1007/s10995-013-1426-3

57. Dodd JM, Mcphee AJ, Turnbull D, et al. The effects of antenatal dietary and lifestyle advice for women who are overweight or obese on neonatal health outcomes : the LIMIT randomised trial. BMC Med. 2014;12(163):1-9.

58. Mudd LM, Pivarnik JM, Pfeiffer KA, Paneth N, Chung H, Holzman C. Maternal Physical Activity During Pregnancy , Child Leisure-Time Activity , and Child Weight Status at 3 to 9 Years. J Phys Act Heal. 2015;12(4):506-514.

59. Vamos CA, Flory S, Sun H, et al. Do Physical Activity Patterns Across the Lifecourse Impact Birth Outcomes? Matern Child Health J. 2015;19(8):1775-1782. doi:10.1007/s10995-015-1691-4

60. Wang C, Zhu W, Wei Y, Feng H, Su R, Yang H. Exercise intervention during pregnancy can be used to manage weight gain and improve pregnancy outcomes in women with gestational diabetes mellitus. BMC Prenancy Childbirth. 2015;15(255):1-8. doi:10.1186/s12884-015-0682-1

61. Dodd JM, Deussen AR, Mohamad I, et al. The effect of antenatal lifestyle advice for women who are overweight or obese on secondary measures of neonatal body composition : the LIMIT randomised trial. An Int J Obstet Gynaecol. 2016;123(2):244-253. doi:10.1111/1471-0528.13796

62. Ronnberg A, Hanson ULF, Nilsson K. Effects of an antenatal lifestyle intervention on offspring obesity – a 5-year follow-up of a randomized controlled trial. Acta Obstet Gynecol Scand. 2017;96(9):1093-1099. doi:10.1111/aogs.13168

63. Lindqvist M, Lindkvist M, Eurenius E, Persson M, Ivarsson A, Mogren I. Leisure time physical activity among pregnant women and its associations with maternal characteristics and pregnancy outcomes. Sex Reprod Healthc. 2016;9(October):14-20. doi:10.1016/j.srhc.2016.03.006

64. Daly N, Mitchell C, Kennelly MM, Farren M, Hussey J, Turner MJ. Maternal obesity and physical activity and exercise levels as pregnancy advances: an observational study. Ir J Med Sci. 2016;185(2):357-370. doi:10.1007/s11845-015-1340-3

65. Barbieri MA, Augusto A. Physical activity in pregnancy and adverse birth outcomes. Cad Saude Publica. 2016;32(11):1-10. doi:10.1590/0102-311X00086915

66. Silva SG da, Hallal PC, Domingues MR, et al. A randomized controlled trial of exercise during pregnancy on maternal and neonatal outcomes : results from the PAMELA study. Int J Behav Nutr Phys Act. 2017;14(175):1-11. doi:10.1186/s12966-017-0632-6

67. Norris T, Mccarthy FP, Khashan AS, et al. Do changing levels of maternal exercise during pregnancy affect neonatal adiposity? Secondary analysis of the babies after SCOPE : evaluating the longitudinal impact using neurological and nutritional endpoints (BASELINE) birth cohort (Cork , Ireland). BMJ Open. 2017;7(11):1-9. doi:10.1136/bmjopen-2017-017987

68. Rodríguez-díaz L, Ruiz-frutos C, Vázquez-lara JM, Ramírez-rodrigo J, Villaverde-gutiérrez C, Torres-luque G. Effectiveness of a physical activity programme based on the Pilates method in pregnancy and labour. Enferm Clin. 2017;27(5):271-277.

69. Huang L, Fan L, Ding P, et al. The mediating role of placenta in the relationship between maternal exercise during pregnancy and full-term low birth weight. J Matern Fetal Neonatal Med. 2018;31(12):1561-1567.

70. Daly N, Farren M, Mckeating A, Stapleton M, Turner MJ, Kelly RO. A medically supervised pregnancy exercise intervention in obese women. Obstet Gynecol. 2017;130(5):1001-1010. doi:10.1097/AOG.0000000000002267

71. Wang C, Wei Y, Zhang X, et al. Reports of Major Impact A randomized clinical trial of exercise during pregnancy to prevent gestational diabetes mellitus. Am J Obstet Gynecol. 2017;216(4):340-351. doi:10.1016/j.ajog.2017.01.037

72. Bacchi M, Mottola MF, Perales M, Refoyo I, Barakat R. Aquatic Activities During Pregnancy Prevent Excessive Maternal Weight Gain and Preserve Birth Weight : A Randomized Clinical Trial. Am J Heal Promot. 2018;32(3):729-735. doi:10.1177/0890117117697520

73. Garnæs KK, Nyrnes SA, Salvesen KÅ, Salvesen Ø, Siv M, Moholdt T. Effect of supervised exercise training during pregnancy on neonatal and maternal outcomes among overweight and obese women. Secondary analyses of the ETIP trial: A randomised controlled trial. Plus One. 2017;12(3):1-15. doi:10.1371/journal.pone.0173937

74. Patel N, Godfrey KM, Pasupathy D, et al. Infant adiposity following a randomised controlled trial of a behavioural intervention in obese pregnancy. Int J Obes(Lond). 2018;41(7):1018-1026. doi:10.1038/ijo.2017.44.Infant

75. Barakat R, Perales M, Cordero Y, Bacchi M, Mottola MF. Influence of Land or Water Exercise in Pregnancy on Outcomes: A cross-sectional study. Med Sci Sport Exerc. 2017;49(7):1397-1403. doi:10.1249/MSS.0000000000001234

76. Hegaard HK, Rode L, Katballe MK, Langberg H, Ottesen B, Damm P. Influence of pre-pregnancy leisure time physical activity on gestational and postpartum weight- a cohort study. J Obstet Gynaecol (Lahore). 2017;37(6):736-741. doi:10.1080/01443615.2017.1292227

77. Badon SE, Mph AJL, Chuen K, Chan G, Williams MA, Enquobahrie DA. Trajectories of maternal leisure-time physical activity and sedentary behavior during adolescence to young adulthood and offspring birthweight. Ann Epidemiol. 2017;27(11):701-707. doi:10.1016/j.annepidem.2017.09.013

78. Dhana K, Haines J, Liu G, et al. Association between maternal adherence to healthy lifestyle practices and risk of obesity in offspring : results from two pro- spective cohort studies of mother-child pairs in the United States. Br Med J. 2018;362(7):1-12. doi:10.1136/bmj.k2486

79. Mcdonald SM, Yeo S, Liu J, Wilcox S, Sui X, Pate RR. Associations between maternal physical activity and fi tness during pregnancy and infant birthweight. Prev Med Rports. 2018;11(December 2017):1-6. doi:10.1016/j.pmedr.2018.04.019

80. Badon SE, Littman AJ, Chan KCG, Williams MA, Enquobahrie DA. Associations of Maternal light/Moderate leisure-time walking and yoga with offspring birth size. J Phys Act Heal. 2018;15(6):430-439.

81. Barakat R, Vargas M, Brik M, Fernandez I, Gil J, Coteron J. Does Exercise During Pregnancy Affect Placental Weight?: A Randomized Clinical Trial. Eval Health Prof. 2018;41(3):400-414. doi:10.1177/0163278717706235

82. Chan RS, Tam W, Ho IC, et al. Randomized trial examining effectiveness of lifestyle intervention in reducing gestational diabetes in high risk Chinese pregnant women in Hong Kong. Sci Rep. 2018;8(February):1-11. doi:10.1038/s41598-018-32285-6

83. Mizgier M, Mruczyk K, Jarząbek-bielecka G, Jeszka J. The impact of physical activity during pregnancy on maternal weight and obstetric outcomes. Ginekol Pol. 2018;89(2):80-88. doi:10.5603/GP.a2018.0014

84. Myrex P, Harper L, Gould S. An evaluation of birth outcomes in overweight and obese pregnant women who exercised during pregnancy. Sports. 2018;6(138):1-7. doi:10.3390/sports6040138

85. Poppel MNM Van, Simmons D, Devlieger R, Assche FA Van, Jans G. A reduction in sedentary behaviour in obese women during pregnancy reduces neonatal adiposity: the DALI randomised controlled trial. Diabetologia. 2019;62:915-925.

86. Hoffmann J, Günther J, Geyer K, et al. Associations between Prenatal Physical Activity and Neonatal and Obstetric Outcomes — A Secondary Analysis of the Cluster-Randomized GeliS Trial. J Clin Med. 2019;8(10):1-14.

87. Brik M, Andez-buhigas IF, Barakat R, Santacruz B. Does exercise during pregnancy impact on maternal weight gain and fetal cardiac function? A randomized controlled trial. Ultrasound Obs Gynecol. 2019;53(April):583-589. doi:10.1002/uog.20147

88. Mcmillan AMYG, May LE, Gaines GG, Isler C, Kuehn D. Effects of Aerobic Exercise during Pregnancy on 1-Month Infant Neuromotor Skills. Med Sci Sport Exerc. 2019;51(8):1671-1676. doi:10.1249/MSS.0000000000001958

89. Barakat R, Refoyo I, Coteron J, Franco E. Exercise during pregnancy has a preventative effect on excessive maternal weight gain and gestational diabetes. A randomized controlled trial. Brazilian J Phys Ther. 2019;23(2):148-155. doi:10.1016/j.bjpt.2018.11.005

90. Clark E, Isler C, Strickland D, et al. Influence of aerobic exercise on maternal lipid levels and offspring morphometrics. Pediatrics. 2019;43:594-602. doi:10.1038/s41366-018-0258-z

91. Jochumsen S, Hegaard HK. Physical activity during pregnancy and intelligence in sons; A cohort study. Scand J Med Sci Sport. 2019;29(March):1988-1995. doi:10.1111/sms.13542

92. Rodríguez-blanque R, Sanchez-garcia JC, Sanchez-lopez AM, Expósito-ruiz M, Aguilar-cordero MJ. Randomized Clinical Trial of an Aquatic Physical Exercise Program During Pregnancy. J Obs Gynecol Neonatal Nurs. 2019;48(3):321-331. doi:10.1016/j.jogn.2019.02.003

93. Dodd JM, Deussen AR, Louise J. A Randomised Trial to Optimise Gestational Weight Outcomes through Antenatal Dietary, Lifestyle and Exercise Advice: The OPTIMISE Randomised Trial. Nutrients. 2019;11(12):2911-2926.

94. Kunath J, Günther J, Rauh K, et al. Effects of a lifestyle intervention during pregnancy to prevent excessive gestational weight gain in routine care – the cluster- randomised GeliS trial. BMC Med. 2019;17(5):1-13.

95. Sundgot-Borgen J, Sundgot-Borgen C, Myklebust G, Solvberg N, Torstveit MK. Elite athletes get pregnant , have healthy babies and return to sport early postpartum. BMJ Open Sport Exerc Med. 2019;5:1-9. doi:10.1136/bmjsem-2019-000652

96. Huang R, Silva D, Beilin L, et al. Feasibility of conducting an early pregnancy diet and lifestyle e-health intervention : the Pregnancy Lifestyle Activity Nutrition (PLAN) project. J Dev Orig Health Dis. 2020;11(May):58-70.

97. Huang L, Fan L, Ding P, et al. Maternal exercise during pregnancy reduces the risk of preterm birth through the mediating role of placenta. J Matern Neonatal Med. 2019;32(1):109-116. doi:10.1080/14767058.2017.1372415

98. Buckingham-schutt LM, Ellingson LD, Vazou S, Campbell CG. The Behavioral Wellness in Pregnancy study : a randomized controlled trial of a multi-component intervention to promote appropriate weight gain. Am J Cli Nutr. 2019;109:1071-1079. doi:10.1093/ajcn/nqy359

99. Rodr R, Eugenia A. Influence of a Water-Based Exercise Program in the Rate of Spontaneous Birth: A Randomized Clinical Trial. Int J Environ Res Public Health. 2020;17(3):795-806.

**Declarations**

**Funding** U.S. National Institute of Health R01-HD067449 and R21-AG049976.

**Conflict of interests** Yanting Chen, Guiling Ma, Qiyuan Yang, Yun Hu, Jeanene Deavila, Meijun Zhu and Min Du declare that they have no conflicts of interest relevant to the content of this review.

**Data Availability Statement** All data are available in submitted manuscript or as electronic supplementary material.

**Code availability** Not applicable.

**Authors’ contributions** All authors contributed to the study design, data interpretation, and revising of the Article. YC, GM, YH, QY, JD screened the studies and extracted and analyzed data. YC, GM, YH, QY, JD, MZ and MD contributed to the literature search, data extraction, and analysis. YT and GM led the quantitative analysis. MZ and MD were expert advisers. All authors read and approved the final manuscript.
